# Articulated specimens provide new insights into the iconic Mesozoic shark genus *Sphenodus*

**DOI:** 10.1080/14772019.2025.2507014

**Published:** 2025-06-20

**Authors:** Eduardo Villalobos-Segura, Manuel Amadori, Sebastian Stumpf, Patrick L. Jambura, Arnaud Begat, Faviel A. Lopez-Romero, Günter Schweigert, Erin E. Maxwell, Jürgen Kriwet

**Affiliations:** a Department of Palaeontology, Faculty of Earth Sciences, Geography and Astronomy, University of Vienna, Josef-Holaubek-Platz 2, 1090 Vienna, Austria; b Natural History Museum Vienna Burgring 7 1010 Vienna, Austria; c Vienna Doctoral School of Ecology and Evolution (VDSEE), University of Vienna, Djerassiplatz 1, 1030 Vienna, Austria; d Unidad Académica de Sistemas Arrecifales, Instituto de Ciencias del Mar y Limnología, Universidad Nacional Autónoma de México, Puerto 7, México; e State Museum of Natural History Stuttgart, Rosenstein 1, 70191 Stuttgart, Germany

**Keywords:** cladistic analysis, *Sphenodus*, Jurassic, Solnhofen Archipelago, fossil shark, cartilaginous skeleton

## Abstract

The fossil record of sharks and rays (Elasmobranchii) consists predominantly of isolated teeth, complicating their taxonomic and phylogenetic inferences. Consequently, the study of holomorphic specimens provides crucial information to reassess existing phylogenetic and taxonomic interpretations. The extinct Mesozoic shark genus †*Sphenodus* exemplifies these challenges. Initially classified based on dental traits, the genus has undergone multiple revisions, reflecting its taxonomic instability. However, the presence of skeletal remains assigned to †’*Sphenodus*’ *macer* and †’*S*.’ *nitidus* provides an opportunity to re-evaluate the phylogenetic and taxonomic relations of this genus. In this study, we critically reviewed the systematics and taxonomic relationships of †*Sphenodus*, through an exhaustive literature review and comparison with previous phylogenetic hypotheses. Our analysis of skeletal characters suggests the synonymy of †*S. macer* and †*S. nitidus* and supports previous Hexanchiformes classifications for the genus, placing it in a close relation to †*Notidanoides* forming a clade in sister relation to Hexanchidae. However, the teeth originally used to define †*Sphenodus* lack diagnostic traits for taxonomic identification, rendering†’*Sphenodus*’ a *nomen dubium*. Consequently, we introduce a new name, †*Archaeogracilidens*, to include the species based on skeletal material and that present teeth with a well-preserved root and a crown, establishing a more stable systematic framework for this group.

## Introduction

Neoselachians *sensu* Compagno (1977) (crown Elasmobranchii *sensu* Maisey et al., 2019) are one of the oldest lineages of vertebrates, dating back to the late Palaeozoic (Ivanov, 2005). However, their major diversification occurred during the Jurassic and Cretaceous periods, shaping their modern diversity (Benton, 2015; Guinot & Cavin, 2016; Guinot et al., 2012; Kriwet et al., 2009; Maisey et al., 2004; Underwood, 2006; Stein et al., 2018).

Despite the relatively continuous fossil record of neoselachians (Maisey, 2012; Maisey et al., 2004; Underwood, 2006), most findings consist of isolated teeth, as sharks shed thousands over their lifetimes and their skeleton is composed predominantly of cartilage, complicating its fossilization. This reliance on dental elements for classification presents challenges, as it assumes that all apomorphic characters evolve simultaneously (Maisey, 2012) and that the preserved characters are diagnostic at the relevant taxonomic level. As a result, many fossil neoselachians, like the shark genus †*Sphenodus* Agassiz, 1843, face taxonomic and phylogenetic uncertainties.

The fossil shark genus †*Sphenodus*, ranges from the Early Jurassic to the Early Cenozoic (Cappetta, 2012; Duffin & Ward, 1993; Kanno et al., 2017). Commonly found in Jurassic deposits across Europe, this genus has traditionally been classified based on dental traits (Cappetta, 2012; Duffin & Ward, 1993). However, the presence of associated cranial and postcranial material (Böttcher & Duffin, 2000; Kriwet & Klug, 2004; Schweigert & Roth, 2021; Thies & Leidner, 2011; Villalobos-Segura et al., 2023) offers the opportunity to refine its classification and provide a new perspective on an ongoing debate regarding its phylogenetic relations within neoselachians (Cappetta, 2012; Cuny & Tabouelle, 2014; Guinot & Cappetta, 2011; Klug, 2010).

Currently there are three contrasting hypotheses regarding the phylogenetic relations of †*Sphenodus*:

1. In early and contemporaneous literature, †*Sphenodus* was associated with lamniform sharks, due to similarities in tooth crown morphology (e.g. Carroll, 1988; de Beaumont, 1960; Glickman, 1957; Maisey et al., 2004; Woodward, 1889).
2. Thies (1983) proposed a sister relation between Hexanchiformes and two fossil families, †Palaeospinacidae and †Orthacodontidae (the later including †*Sphenodus*), forming an unnamed group within neoselachians. Duffin and Ward (1993) later named this group Synechodontiformes, placing it within Compagno’s (1973) Squalomorphii, a classification further supported by Leidner and Thies (1999) and Böttcher and Duffin (2000). Klug (2010) later suggested that Synechodontiformes was a monophyletic group in a sister group relation to all neoselachians. These classifications are mainly based on dental features (e.g. the root vascularization pattern).
3. Guinot and Cappetta (2011) proposed an affiliation within Hexanchiformes, suggesting that both Synechodontiformes and Hexanchiformes form part of neoselachians. This classification is based on similarities in enameloid microstructure.

To clarify the taxonomic and phylogenetic relationships of †*Sphenodus*, we conducted a comprehensive specimen and literature review, comparing previous classifications and phylogenetic hypotheses. Our revision provides additional information for the classification of †*Sphenodus* within the neoselachians, incorporating both known and newly analysed skeletal characters.

### Geological and environmental context

All holomorphic specimens were recovered from the Late Jurassic lithographical limestones of the so-called Solnhofen Archipelago in Bavaria and the Nusplingen locality in Baden-Württemberg. Both of these German sites represent complex lagoonal environments situated at the north-western edge of the Tethys Ocean (Pan et al., 2019; Viohl, 2015a, 2015b).

The fossil deposits of the Solnhofen Archipelago are mostly found in the southern part of the Franconian Alb, part of the Franconian-South Bavarian carbonate shelf, formed by siliceous sponge-microbial bioherms (Schmid et al., 2008; Viohl, 1996). The laminated lime-stone deposits suggest the presence of hostile conditions in the basins due to the restriction of water exchange between the basins and the open ocean, leading to high evaporation rates for the stagnant waters, resulting in anoxic and hypersaline water conditions (Pan et al., 2019; Viohl, 2015a, 2015b). These conditions, combined with rapid burial during periodic storm events, contributed to the exceptional preservation of marine and terrestrial fossils remains of plants and animals that characterized the Southern Franconian Alb by preventing decomposition and scavenging activities (Mäuser, 2015; Schweigert, 2015). Spanning about 3.5 million years (late Kimmeridgian–early Tithonian), these lime-stones encompass diverse depositional and stratigraphical settings, classified into different formal geological formations (Niebuhr & Pürner, 2014). Most of the historically collected fossils come from deposits from the early Tithonian Altmühltal Formation (formerly ‘Solnhofener Plattenkalke’), quarried for practical uses since Roman times (Neumeyer, 2015).

The fossil site of Nusplingen (Nusplingen and Egesheim quarries), in the south-western part of the Swabian Alb (Baden-Württemberg), is a small isolated outlier of the Solnhofen Archipelago, covering an area of about 2.5 km^2^, in which a 10–15-m-thick section of finely laminated limestones is exposed (Dietl et al., 1998; Schweigert & Roth, 2021). This sedimentary succession dates to the late Kimmeridgian (Schweigert, 2015) and was deposited in a locally restricted shallow (less than 100-m-deep) lagoonal depocentre with stagnant seafloor conditions (Dietl & Schweigert, 2004; Hättig et al., 2019). Systematic excavations since 1993 have revealed a highly diverse assemblage of plants and animals, making it one of the world’s most productive Jurassic fossil sites (Schweigert & Roth, 2021).

## Material and methods

### Fossil material

Four more or less complete skeletal remains (SMNS 96844/7, SMNS 80142/44, SNSB-BSPG AS VII 647, MCZ 133896) as well as three isolated teeth (associated to the genus based on dental resemblance; see below) (SMNS 80142/6, SMNS 86244, EMRG-Chond-T-78) form the basis of this study. Specimens SMNS 96844/7 and SMNS 80142/44 were collected from the upper Kimmeridgian deposits of Nusplingen and Egesheim, respectively. SNSB-BSPG AS VII 647 was collected from the lower Tithonian deposits of the Solnhofen Archipelago. MCZ 133896 was recovered from the Upper Jurassic (most likely also lower Tithonian) of Articulated specimens of the Mesozoic shark genus *Sphenodus* 3 the Solnhofen Archipelago. SMNS 80142/6 comes from the upper Kimmeridgian deposits of Nusplingen. SMNS 86244 was collected from the Oxfordian (Upper Jurassic) deposits near the village of Houlgate and Villers–sur–Mer, Normandy (France). EMRG-Chond-T-78 comes from the lower Tithonian (Upper Jurassic) ‘Mörnsheimer Formation’ of Schaudiberg near Mühlheim of the Solnhofen Archipelago.

### Terminology

The nomenclatural terminology used here follows the standards proposed by Matthews (1973), Bengtson (1988) and Sigovini et al. (2016). The description and nomenclature of cranial and postcranial structures follows Maisey (1985, 1986, 2004, 2007, 2008), Shirai (1992a), de Carvalho (1996), Maisey et al. (2004), da Silva and de Carvalho (2015) and da Silva and Datovo (2020). The tooth terminology follows that of Cappetta (1987, 2012).

### Imaging methods

All specimens reviewed in the present work were studied in their corresponding museum collections. Photographs documenting morphological traits were prepared using a Nikon camera with a mounted 44 mm macro lens and a Canon Camera with a mounted 105 mm macro lens. One isolated tooth identified as †*Sphenodus* (EMRG-Chond-T-78) was analysed using a SkyScan1173 micro-CT (Bruker/Skyscan, Kontich, Belgium) at the Department of Palaeontology of the University of Vienna, Austria to study its histology non-invasively. The scan was performed at 5 μm resolution with the following settings: 100 kV source voltage, 80 μA source current, 750 ms exposure, 0.2° rotation step, and using a 1.0 mm aluminium filter provided with the device. The resulting stack file was loaded into the software system Amira v. 5.4.5 to create the isosurface and virtual sections through different planes of the examined tooth (see http://morphobank.org/permalink/?P5764 for 3D reconstruction of the teeth). Following this procedure, the specimen was sectioned, to provide additional information and to confirm the first observations made with the CT scans. The illustrative drawings and images of the fossils were prepared using the software packages Illustrator v27.6.1 and Photoshop CS6 v.23.5.5.

### Phylogenetic analyses

Considering that previous phylogenetic analyses of the relations of †*Sphenodus* were conducted under parsimony criteria (e.g. Klug, 2010), the present study employs the same framework to analyse the two holomorphic specimens (SMNS 96844/7 and SMNS 80142/44). This was done to facilitate the comparison between our results with prior studies. To revise our results, an additional analysis was conducted under the Mkv model (Lewis, 2001) in Mr. Bayes v.3.2.7a (Ronquist et al., 2012) (see Supplemental material).

The character matrix utilized in this study is adapted from Vullo et al. (2024), which provides an extensive sampling of neoselachian and other elasmobranch groups, along with an extensive bibliographical review. When needed, modifications were made to some characters and terminal taxa in this matrix using Mesquite v.3.61 (Maddison & Maddison, 2023) (see Supplemental material). The parsimony analysis was conducted in TNT v.1.6 (Goloboff & Morales, 2023), following the protocol outlined by Goloboff et al. (2021) for handling characters with logical dependences. Implied weighted approaches were considered and tested for using the scripts ‘moprhotestall’ and ‘morphodoaic’ (Goloboff & Arias, 2019).

The search for the most parsimonious trees (MPTs) was conducted with the deactivation of invariable characters and under the collapse rule 3 (default for TNT). The search-parameters included a combination of 100 iterations of ratchet parsimony and five rounds of tree fusing, with 10 replications with the search terminating upon the retrieval of 10 hits of the minimum score. To ensure a comprehensive exploration of the tree space, this search was repeated 10 times using different random seed numbers. Given that all searches resulted in variable numbers of most parsimonious trees (MPT’s) of identical length and identical strict consensus, an additional search was conducted applying the command ‘bbreak’ to estimate the additional trees. The trees recovered from this search were kept and used to estimate the strict consensus tree.

Clade support was calculated via a regular jackknife analysis (using the ‘resample’ and ‘jak’ commands), with 1000 replications to calculate the absolute clade frequencies within the strict consensus tree. Default settings were maintained for all other parameters. Bremer support values were also estimated (see Supplemental material).

The Bayesian inference analysis was conducted under a Mkv model with gamma-distributed rate variation (approximated using eight categories), with a model selection guided by IQ–tree (Trifinopoulos et al., 2016). Following the recommendations of Clarke and Middleton (2008), unlinked partitions were implemented to account for across characters rate variation, with the partition scheme inferred in R (R Core Team, 2024) using the R package EvoPhylo (Simões et al., 2023).

The Bayesian analysis incorporated four independent runs, with four chains. The following search parameters were applied: ngen = 7,800,00; samplefreq = 1000; printfreq = 1000; stopval = 0.01; stoprule = yes; temp 0.05; relburnin = yes; nperts = 2; savebrlens = yes; append = yes; with a burn-in fraction of 30% (see Supplemental material).

The consensus trees resulted from all analyses (strict consensus trees for parsimony and majority-rule consensus tree for Bayesian inference) were compared in TNT estimating the number of SPR moves separating each topology and their similarity score.

### Institutional abbreviations

**EMRG-Chond-T**, Evolutionary and Morphology Research Group of the University of Vienna chondrichthyan collection, teeth, Vienna, Austria; **GPIT**, Paleontological Research, Teaching and Display Collection, Institute of Geosciences of the University of Tübingen, Tübingen, Germany; **MCZ**, Museum for Comparative Zoology, Cambridge, USA; NHMUK **PVP**, Natural History Museum United Kingdom, London, UK, Palaeontology Vertebrate, Pisces; **SMNS**, State Museum of Natural History Stuttgart, Stuttgart, Germany**; SNSB-BSPG**, State Natural Science Collections of Bavaria – Bavarian State Collection for Palaeontology and Geology, Munich, Germany.

### Systematic palaeontology

Class **Chondrichthyes** Huxley, 1880

Subclass **Elasmobranchii** Bonaparte, 1838

Order **Hexanchiformes** de Buen, 1926

Family †**Orthacodidae** Glickman, 1957

#### Type genus

†’*Orthacodus*’ Woodward, 1889 (*nomen dubium*), which is junior synonym of †’*Sphenodus*’ Agassiz, 1843 (*nomen dubium*). We propose here †*Archaeogracilidens* (nom. nov.) as replacement for †’*Sphenodus*’ (*sensu* Quenstedt, 1865). See below for more details.

#### Amended diagnosis

Based on dental traits from de Beaumont (1960, p. 29). A clade of extinct hexanchiform sharks characterized by teeth with osteodont histology; labial crown face of teeth convex transversally; crown with a high main cusp and generally with a single pair of lateral cusplets adjacent to the main cusp; main cusp with well-developed cutting edges; tooth root not divided into two root lobes; root shape rounded to oval with flat or slightly convex basal surface, which is perpendicular to oblique in relation to main cusp; labial portion of root with short, parallel nutritive grooves, sometimes also present lingually.

#### Nomenclatural notes

According to the ICZN (1999, art.13.1), any scientific name (family-group names included) published after 1930 is available and officially established only if the designation is associated with a description or definition that clearly states the characters that differentiate the taxon (diagnosis) or to a bibliographical reference of a such published definition, or when proposed as replacement name. In addition, family names have to be established based on an available genus and, subsequently, used as valid (ICZN, 1999, art.13.2). However, family-group names published between 1930 and 1961 that do not meet the requirements of art. 13.1 (see above) are to be considered as established and available only if they were used as valid before 2000 and not rejected by applying art. 13 between 1960 and 2000 (see ICZN, 1999, art.13.2.1).

Glickman (1957, p. 117) introduced the family name †Orthacodidae without providing any definition of the group, and assigned the genera †*Orthacodus* Woodward, 1889, †*Paraorthacodus* Glickman, 1957 and †*Eychlaodus* Glickman, 1957 to it (see also Popov, 2016, p. 31). Glickman (1957) based the name of his new family on †*Orthacodus* representing thus the type genus, which was regarded and used as valid (although erroneously) at that time. Even if the type genus is subsequently found to be invalid (e.g. junior synonym or a *nomen dubium*; see below), the family name is not to be replaced (see ICZN, 1999, art. 40.1). In addition, †Orthacodidae was used later as a valid family in two publications (e.g. Glickman, 1958a, 1958b) and never rejected applying art. 13 of any ICZN code available in the past (see also Van der Laan, 2018). Consequently, †Orthacodidae Glickman, 1957 has to be considered as correctly established according to the ICZN (1999).

The family name †Orthacodontidae was established by de Beaumont (1960, pp. 29, 38) based on the genus †’*Orthacodus*’, which was available and valid at the time, together with a definition useful to differentiate it (†’*Orthacodus*’). †Orthacodontidae de Beaumont, 1960 is therefore also to be regarded as correctly established, but as a junior synonym of †Orthacodidae Glickman, 1957 according to the ICZN (1999, art. 23). Later, Cappetta (1987, p. 49) replaced †Orthacodidae (senior synonym) with †Orthacodontidae (junior synonym) according to the ICZN code available at the time (see ICZN, 1985, art. 23 and Appendix D ‘VII’; see also Böttcher & Duffin, 2000, p. 5). According to the ‘Principle of Priority’ stated in art. 23 of the ICZN (1985), the valid name of a taxon (family-group names included) is the oldest available name – that is †Orthacodidae – if not previously invalidated (see also Articulated specimens of the Mesozoic shark genus *Sphenodus* 5 ICZN, 1999). Section ‘VII’ of the ICZN (1985, Appendix D) included a series of ‘Tables and explanatory notes compiled as an aid to zoologists’ that are useful for scientific name composition. The original composition of the name †Orthacodidae neither contradicts the ICZN of 1985 (Appendix D, ‘VII’) nor the most recent ICZN (1999, art. 29) rules. Consequently, there is no reason for accepting the name replacement proposed by Cappetta (1987). The family †Orthacodidae is thus to be considered valid with the original spelling according to the ICZN (1999, art. 11.7, 13.2, 29.2, 29.5, 35, 40 and 63; see also Van der Laan, 2018).

#### Genera included

†*Archaeogracilidens* gen. nov. (type genus) and †*Occitanodus* Guinot et al., 2014. In contrast to †*Archaeogracilidens* gen. nov., the type species of †*Eychlaodus* Glickman, 1957 clearly has teeth with bilobate roots (see also below). Teeth of †*Paraorthacodus* Glickman, 1957 possess a pseudoos-teodont tooth histology (see Jambura et al., 2020). Thus, the affiliation of both †*Paraorthacodus* and †*Eychlaodus* to this family is rejected here. Conversely, †*Occitanodus* Guinot et al., 2014 is here considered as belonging to this family based on the similar tooth morphology with †*Archaeogracilidens*, pending a revision of the tooth histology for the genus.

Genus †***Archaeogracilidens*** gen. nov.

#### Type species

†*Archaeogracilidens macer* (originally †*Oxyrhina macer* Quenstedt, 1851); see nomenclatural remarks (below) for more details.

#### Type locality and horizon

Schnaitheim Heidenheim a.d. Brenz, Baden-Württemberg, Brenztaltrümmerkalk Subformation (Late Kimmeridgian, Beckeri Zone, Ulmense Subzone).

#### Derivation of name

The generic name is derived from ‘Archaeo’ [*αρχαíος*] (m.), the Greek word for ‘ancient’, ‘antique’, and a combination of the Latin words ‘gracilis’, meaning ‘slender’ in allusion to the very slender crowns in anterior teeth, and ‘dentis’, which is the genitive singular of ‘dens’ (m.), meaning tooth, in allusion to the high number of species based on teeth alone.

#### Diagnosis

As type species (by monotypy) (see below).

#### Nomenclatural remarks

In the past, teeth characterized by a narrow, laterally and lingually curved cusp and non-lobed root, also observed in specimens SMNS 80142/44, SMNS 96844/7, MCZ 13389 and SNSB-BSPG AS VII 647 examined here, generally were assigned to the genus †’*Sphenodus*’ Agassiz, 1843 (e.g. Böttcher & Duffin, 2000; Cusumano et al., 2021; Fricke, 1875; Guinot et al., 2014; Quenstedt, 1865; Stumpf & Kriwet, 2019; Trautschold, 1877). Agassiz (1843, p. 288) originally introduced the name †’*Sphenodus*’ as a subgenus of *Lamna* Cuvier, 1816 and referred nine isolated teeth from Jurassic–Early Cretaceous sediments of Europe (see Agassiz, 1844, pl. 37, figs 24–32). Six of these teeth were assigned to *Lamna* (†’*Sphenodus*’) *longidens* (Agassiz, 1843, pp. 298–299; see also Agassiz, 1844, pl. 37, figs 24–29), while the remaining three were assigned to *Lamna* (†’*Sphenodus*’) *plana* (vol. 3, pl. 37, figs 30–32). Although Agassiz considered the morphologies of these teeth peculiar, the lack of the root in the six teeth assigned to *L*. (†’*Sphenodus*’) *longidens* (see Agassiz, 1844, pl. 37, figs 24–29) prevented him from establishing a new genus. The three teeth assigned to *L*. (†’*Sphenodus*’) *plana* by Agassiz (1843) present a morphology almost indistinguishable from that typically associated with *Lamna sensu lato* (see Agassiz, 1844, pl. 37, figs 30–32). According to the International Code of Zoological Nomenclature (ICZN, 1999: art. 6.1), the name †’*Sphenodus*’ placed in parentheses by Agassiz (1843) serves the function of a subgenus rather than a genus, which would be in accordance with Agassiz’s use.

Giebel (1848, p. 364) was the first to use †’*Sphenodus*’ Agassiz, 1843 at the genus level (official ‘elevation in rank’) to include the two species established by Agassiz (1843). According to the ICZN (1999, art. 12), new names published before 1931 are available and officially established, when they meet the requirements of art. 11 and are accompanied by a description or a definition of the taxon, or by another indication (e.g. illustrations or bibliographical reference, see arts. 12.2.5 and 12.2.7) that refers to the type species. Agassiz (1843) in fact was the first to provide a description associated with the name †’*Sphenodus*’, even though he used it as a subgenus, and the species in it (*Lamna* (†’*Sphenodus*’) *longidens* and *L*. (†’*Sphenodus*’) *plana*). Consequently, the official name for the genus is †*Sphenodus* (see also Principle of Priority in ICZN, 1999, art. 23.3.1). However, major doubts remain as to the validity of the genus †’*Sphenodus*’ for taxonomic purposes due to the dubious and very fragmentary nature of the type material (see above) used by Agassiz (1843). According to the Principle of Typification (ICZN, 1999, art. 61), the name-bearing type material of a nominal taxon has to provide the objective standard of reference for the application of the scientific name linked to it. The types used for the original establishment of *Lamna* (†*Sphenodus*) *longidens* Agassiz, 1843 and *L*. (†*Sphenodus*) *plana* Agassiz, 1843, and consequently †’*Sphenodus*’ (see Principle of Coordination in ICZN, 1999, art. 43), do not provide clear characters for an accurate and unambiguous application of the genus name. A name that is not applicable for taxonomic purposes must be considered a ‘*nomen dubium*’ and consequently is invalid (see Glossary section of ICZN, 1999). Although available under the rules of the ICZN (1999, art. 12 and art. 10.6), the original species (†’*S*.’ *longidens* and †’*S*.’ *plana*) and, consequently, the genus name †’*Sphenodus*’ Agassiz, 1843 are thus *nomina dubia* and we strongly discourage their future use for taxonomic purposes.

According to the ‘Principle of Priority’ (see ICZN, 1999, art. 23.3.5), a taxon regarded as invalid has to be replaced with the oldest available synonymous name once the validity of the chosen substitute name is verified. Quenstedt (1851, p. 172) transferred the species †’*Sphenodus*’ *longidens* Agassiz, 1843 to the genus †*Oxyrhina* Agassiz, 1838, which also was established by Agassiz (1838, p. 86, pls 33, 34) to include teeth with *Lamna*-like morphology but lacking lateral cusplets. Moreover, all species originally included in †*Oxyrhina* (†*O. hastalis*, †*O. mantelli*, †*O. retroflexa*, †*O. xiphodon*) have large, triangular and blade-like cusps with distinctly bilobate roots and a narrow-to-wide nutritive groove on the lingual root protuberance (see Agassiz, 1838, pls 33, 34). The tooth morphology assigned to †’*Sphenodus*’ by Agassiz (1843) clearly differs from those of †*Oxyrhina* in having a much narrower and more slender cusp (see Agassiz, 1843, 1844). Therefore, †*Oxyrhina* cannot be considered a junior synonym of †’*Sphenodus*’ and thus is not a suitable candidate name to replace †’*Sphenodus*’. Woodward (1889, p. 349) introduced the name †’*Orthacodus*’, as junior synonym of †’*Sphenodus*’ and used †’*Orthacodus longidens*’ (previously †’*Sphenodus longidens*’ Agassiz, 1843) as the type species of his new genus. Due to the inapplicability of the type species (†’*S. longidens*’, see above), †’*Orthacodus*’ also has to be regarded as a *nomen dubium* (see also Principle of Coordination in ICZN, 1999, art. 43).

Glickman (1957, p. 116, 117) established the genus †*Eychlaodus* based on the species †*Eychlaodus lundgreni* (originally †*Oxyrhina lundgreni* Davis, 1890) from the Late Cretaceous and Danian of Denmark and Sweden. Davis (1890, pl. 39, figs 8–13) initially assigned six teeth, most of which are fragmentary and lacking their root, to this species. Among them, only one specimen still embedded in matrix has the root preserved (see Davis, 1890, pl. 39, fig. 9) and clearly shows basally extended root lobes as it is typical for teeth of many lamniforms, such as odontaspidids and carcharinids. In addition, Glickman (1957, fig. 1, 16) erroneously assigned an isolated tooth from the Late Cretaceous of Russia to †*E. lundgreni*. This isolated specimen clearly exhibits a low and laterally extended root; the latter is nowadays commonly considered typical of †’*Sphenodus*’. Since †*Eychlaodus lundgreni* is the only species ever included in the genus †*Eychlaodus*, this taxon bears the objective standard of reference (type species) for the application of Glickman’s genus. Based on the type material (six teeth, see above), †*E. lundgreni*, and consequently the whole genus, has a bilobate root. Because of the aforementioned character difference, †*Eychlaodus* is rejected here as a possible replacement name for †’*Sphenodus*’. Glickman’s isolated tooth is to be reassigned to the species †*Oxyrhina macer* Quenstedt, 1851 (now †*Archaeogracilidens macer*, see below). In the absence of valid synonyms or substitute names for a rejected taxon (here †’*Sphenodus*’ Agassiz, 1843), a new name has to be established in its place (see ICNZ, 1999, art. 23.3.5 and below). We therefore use inverted commas, i.e. †’*Sphenodus*’ to comply with this requirement.

Quenstedt (1851, pp. 172–173) established two new species, †*Oxyrhina macer* and †*Oxyrhina ornati* based on several isolated teeth. Quenstedt (1851, pl. 13, fig. 18) also figured one of the types of †*O. macer* having a narrow, curved main cusp and a ‘bar-like’ root with no lobes (see also the sections ‘type material, below). The figured tooth of †*O. macer* gives unambiguous, species-specific characters useful for a proper taxonomic identification (see also Quenstedt, 1882, pl. 20, figs 39, 40). Thus, the species †*O. macer* has to be considered as valid (see ICZN, 1999, art. 12). The type (isolated tooth) of †*O. ornati* figured by Quenstedt (1851, pl. 13, fig. 13) shows tooth features almost identical to those of †*O. macer* but with a higher central cusp (see also Quenstedt, 1882, pl. 20, fig. 42). Both morphologies (high- and low-cusped) are also clearly recognizable in the type specimen of †’*Sphenodus*’ *nitidus* Wagner, 1862 holomorphic specimen SNSB-BSPG AS VII 647; see also below), providing both tooth and skeletal traits (Wagner, 1862, p. 290, pl. 1, fig. 4). de Beaumont (1960, p. 21) and Schweizer (1964, p. 83) considered †*Oxyrhina macer* (later †’*Sphenodus*’ *macer*) and †’*S*.’ *nitidus* as synonyms based on the strong similarities in tooth morphology. Following the ICZN (1999, art. 23), both †*Oxyrhina ornati* and †’*Sphenodus*’ *nitidus* are considered here junior synonyms of †*O. macer* (senior synonym). However, the dental features of †*O. macer* do not match those of the genus †*Oxyrhina* Agassiz, 1838 (see above). Consequently, the species must be reassigned to another genus. The species-specific features of †*O. macer* are identical to those commonly attributed to †’*Sphenodus*’ and are well recognizable in all specimens examined here (SMNS 80142/44, SMNS 96844/7, MCZ 13389, SNSB-BSPG AS VII 647, SMNS 80142/6, SMNS 86244 and EMRG-Chond-T-78). Therefore, †*O. macer* is selected here as type species of a new genus (†*Archaeogracilidens*) to replace †’*Sphenodus*’. However, it must be emphasized that †’*Sphenodus*’ and †*Archaeogracilidens* are not to be considered synonymous. †’*Sphenodus*’ continues to exist, but refers exclusively to the dubious and poorly preserved material (e.g. broken cusps) originally assigned to the type species of the genus by Agassiz (1843, 1844). Following the ICZN (1999, art. 13 and 61), †*Archaeogracilidens* is instead established here to include only well-preserved specimens (e.g. complete teeth and skeletal material) conforming to the generic diagnosis (see below).

†***Archaeogracilidens macer*** (Quenstedt, 1851) (Figs 1–9)

Selected synonymy list:

*1851 *Oxyrhina macer*; Quenstedt: 172, pl. 13, fig. 18.

1851 *Oxyrhina ornati*; Quenstedt: 172, pl. 13, fig. 13.

1857 *Oxyrhina ornati*; Quenstedt: 467, pl. 63, fig. 5.

v1861 *Sph. nitidus* Wagn. Wagner: 14, pl. 1, fig. 4.

v1862 *Sph. nitidus* Wagn. Wagner: 290, pl. 1, fig. 4.

1865 *Sphenodus macer*; Quenstedt: 211.

1865 *Sphenodus ornati*; Quenstedt: 211.

1866 *Sphenodus macer*; Quenstedt: pl. 13, fig. 18.

1866 *Sphenodus ornati*; Quenstedt: pl. 13, fig. 13.

1875 *Sphenodus macer*, Quenstedt; Fricke: 394, pl. 4, figs 21, 21a.

1882 *Oxyrhina macer*; Quenstedt: 271, pl. 20, figs 39, 40.

1882 *Oxyrhina ornati*; Quenstedt: 271, pl. 20, fig. 42.

p.1960 *Orthacodus nitidus* Wagn.; de Beaumont: 11, figs 8–15 (*non* 19).

1960 *Orthacodus* (*Cladodus*) *Stschurowskii* (sic.) Kiprijanoff; de Beaumont: 22, fig. 20.

1964 *Sphenodus macer* (Quenst.); Schweizer; 83.

1987 *Sphenodus* Agassiz, 1843B; Cappetta: 50, fig. 49 (non syn.).

1987 *S. nitidus* (Wagner, 1862A); Cappetta: 50.

v2000 *Sphenodus longidens* Agassiz, 1843; Böttcher and Duffin, 2000: 5, figs 16B, 17C, 18A.

v2000 *Sphenodus macer* (Quenstedt, 1851); Böttcher and Duffin, 2000: 5, figs 3–12, 17B, 18C, pl. 1 (non syn.).

v2000 *Sphenodus nitidus* Wagner, 1862; Böttcher and Duffin, 2000: 16, figs 13–15, 16A, 17A, 18B, pl. 2. (non syn.)

v2004 *Sphenodus macer*; Kriwet and Klug: 75, fig. 6a, b.

v2004 *Sphenodus nitidus* Wagner, 1861; Kriwet and Klug: 68, fig. 6c.

v2010 *Sphenodus macer*; Klug: 39, fig. 1E.

2011 *Sphenodus sp*.; Guinot & Cappetta: 197, fig. 2. v2011 *Sphenodus nitidus* Wagner, 1862; Thies and Leidner: 80, pl. 69.

v2011 *Sphenodus macer* (Quenstedt, 1851); Thies and Leidner: 79, pls 67,68.

2014 *Sphenodus nitidus* (Wagner, 1862); Guinot et al.,: 61, figs 3N’-S’ and 4A–J (non syn.).

2012 *Sphenodus* Agassiz, 1843B; Cappetta: 100, fig. 88 (non syn.).

2012 *S. macer* (Quenstedt, 1852); Cappetta: 100. 2012 *S. nitidus* (Wagner, 1862A); Cappetta: 100. 2015 *Sphenodus nitidus*; Schweigert et al., 243, pl. 7

v2021 *Sphenodus nitidus*; Schweigert and Roth, 351, fig. 4

v2023 *S. nitidus* (Quenstedt, 1851A); Villalobos-Segura et al.: 22, figs 13^a^, B.

#### Type material

Quenstedt (1851, p. 172) originally established the species under the name †*Oxyrhina macer* based on several isolated teeth from the ‘Weißjura e’ (upper Kimmeridgian) of Schnaitheim, Heidenheim a.d. Brenz (Baden-Württemberg, southern Germany) and figured one of them in his fig. 18a, b of pl. 13 (see also Böttcher & Duffin, 2000, p. 5). The latter tooth has a slender, sharply pointed central cusp and a shallow root with a flat basal surface. The type series used by Quenstedt (1851) to establish the species is supposed to be housed in the collection of the University of Tübingen. Unfortunately, no tooth labelled or identified as the original of Quenstedt’s figure could be found in the Tubingen collections (see Böttcher & Duffin, 2000, pp. 5–6, fig. 11).

Nonetheless, Böttcher and Duffin (2000, pp. 5–6, fig. 11) selected an isolated tooth (GPIT-PV-65780 (GPIT 1205/15) to serve as a lectotype according to ICZN (1999, art. 74). This specimen was previously figured by de Beaumont, (1960, pl. 2, fig. 16), superficially resembles the illustration of Quenstedt (1851, pl. 13 fig. 18a, b), and comes from the same type locality. Specimen GPIT-PV-65780 is here considered as the lectotype following Böttcher and Duffin (2000), until the status of the specimen with regard of the type series is clarified (see ICZN, 1999, art. 74.2). However, it must be stated that the designation of a lectotype carries the effect that this specimen becomes the sole name-bearing type of that nominal taxon, depriving all other specimens that were formerly syntypes of that nominal taxon of the status of syntype; those specimens become paralectotypes (ICZN, 1999, arts. 73.2.2 and 74.1).

#### Additional material (examined by the authors)

**Figure 1:**
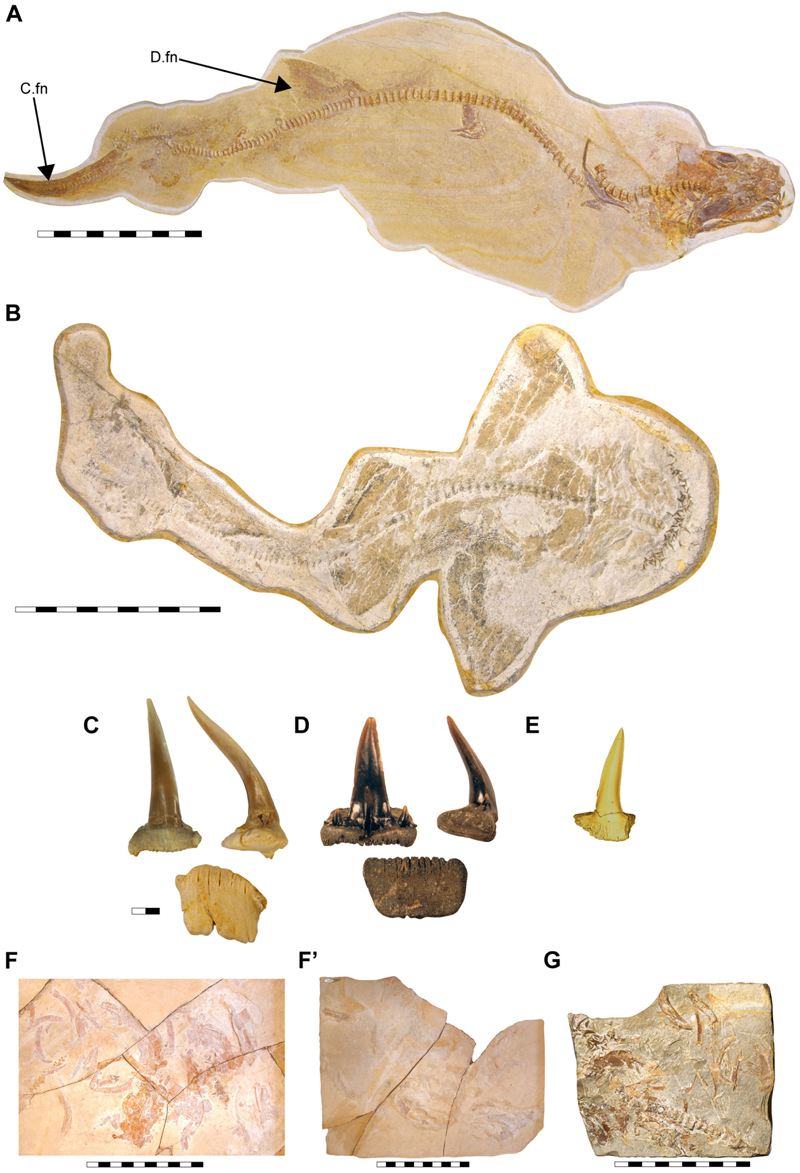
Specimens of †*Archaeogracilidens macer* included in the present study. **A**, SMNS 96844/7 (dorsal surface); **B**, SMNS 80142/44 (ventral surface); **C**, SMNS 80142/6 Isolated tooth in labial, lateral and basal views; **D**, SMNS 86244 Isolated tooth in labial, lateral and basal views; **E**, EMRG-Chond-T-78 volumetric reconstruction of an isolated tooth, in labial view; F, MCZ 133896 (Part); **F’**, MCZ 133896 (Counterpart); **G**, SNSB-BSPG AS VII 647. **Abbreviations: C.fn**, upper lobe of caudal fin; **D.fn**, dorsal fin. Scale bars = 20 cm in A, B, F, G; 2.5mm in C–E.

Specimen SMNS 96844/7 (Fig. 1A) originates from the Upper Jurassic (upper Kimmeridgian, Beckeri Zone, Ulmense Subzone) of Nusplingen Baden-Württemberg, SW Germany. It represents an almost complete female skeleton of approximately 106.3 cm in length, measured along the vertebral column. The dorsal side of the specimen underwent complete mechanical preparation at the State Museum of Natural History Stuttgart, with additional preparation on the ventral surface of the cephalic region. This specimen retains an almost complete vertebral column, dorsal and ventral portions of the jaw cartilages, parts of the pectoral and pelvic girdles, the dorsal fin, and the distal portion of the caudal fin. The majority of the preserved teeth are articulated to the lower jaw and can be classified into anterior, lateral, posterior and commissural positions.

Specimen SMNS 80142/44 comes from the Upper Jurassic (upper Kimmeridgian, Beckeri Zone, Ulmense Subzone) of Nusplingen, Baden-Württemberg, SW Germany. It represents an almost complete female skeleton of approximately 80 cm in length, measured along the vertebral column. The ventral side of the specimen was fully mechanically prepared at the State Museum of Natural History Stuttgart, with additional preparation of the dorsal surface of the cephalic region. The specimen exhibits a disarticulated and mixed dentition, as well as dorsal and ventral portions of the Meckel’s and palatoquadrate cartilages and neurocranium, the ventral surface of the branchial skeleton, pectoral girdle and pectoral fins, vertebral column, pelvic fins, parts of the caudal fin and large areas of skin with articulated scales (Fig. 1B).

Specimen SMNS 80142/6 is an isolated tooth from the Upper Jurassic (upper Kimmeridgian) of Nusplingen. This specimen likely originates from an anterior or anterolateral position and preserves a high and slender crown and shallow root (Fig. 1C).

Specimen SMNS 86244 is an isolated tooth from the Oxfordian (Late Jurassic), from marine–littoral deposits near the village of Houlgate and Villers-sur-Mer, Normandy (France). It probably originates from a posterolateral tooth file and preserves a high and slender crown with lateral cusplets and a shallow root (Fig. 1D).

Specimen EMRG-Chond-T-78 is an isolated tooth from the lower Tithonian of Mühlheim (Mörnsheim, Bavaria, Germany). It probably comes from a lateral position and preserves a slender crown and shallow root (Fig. 1E).

Specimen MCZ 133896 is a fragmentary and disarticulated skeleton from the lower Tithonian Altmühltal Formation of the Solnhofen Archipelago (exact provenance unknown). It represents part and counterpart of a specimen, with preserved parts of the Meckel’s and palatoquadrate cartilages, neurocranium, teeth and various disarticulated cartilaginous remains as well as vertebral centra (Figs 1F, F’).

Specimen SNSB-BSPG AS VII 647 is a fragmentary and disarticulated skeleton from the lower Tithonian Altmühltal Formation (Obere Solnhofen Subformation) of the Solnhofen Archipelago (exact provenance unknown). It is highly incomplete, preserving only anterior parts of the skeleton, including fragments of the neurocranium, jaw cartilages, branchial elements and pectoral girdles and fins, along with several teeth and vertebral centra (Fig. 1G).

#### Amended diagnosis

An orthacodid shark characterized by the following combination of plesiomorphic and autapomorphic characters (autapomorphic characters are indicated by an asterisk): Disjunct monognathic dental heterodontic arrangement in lower jaw, with anterior and anterolateral teeth presenting high, slender, and sharply accentuated central cusps and lateral heels without any lateral cusplets. Tooth crowns in anterior teeth sigmoidal in profile view, becoming less pronounced in lateroposterior teeth. Tooth crown height decreasing gradually from mesial to distal. Lateral-posterior teeth displaying a single pair of lateral cusplets adjacent to main cusp *. Posterior and commissural teeth with a strongly reduced central cusp. Tooth ornamentation restricted to fine short vertical striations along basal margin of lingual crown face*. Root positioned perpendicular to the crown forming a right basal angle. Root becoming progressively lower distally and expanding more lingually. Basal root surface flat*. Labial portion of the root presenting nutritive grooves, which occasionally also are developed lingually. Root vascularization of non-compact pseudopolyaulacorhize type. Quadrate process of palatoquadrate well developed. Adductor fossa on palatoquadrate deep, dorsally outlined by a rounded ridge. Single dorsal fin, placed posteriorly, between pelvic girdle and anal fin.

#### Material

Two holomorphic specimens (SMNS 80142/44 and SMNS 96844/7), two partially articulated specimens (MCZ 133896 and SNSB-BSPG AS VII 647) and three isolated teeth (SMNS 80142/6, SMNS 86244 and EMRG-Chond-T-78).

#### Remarks

Measurements and body proportions are difficult to establish, due to the fragmentary nature of the remains. All specimens show different degrees of disarticulation, suggesting different seafloor exposure times. Nevertheless, they unambiguously can be referred to †*Archaeogracilidens* based on their identical tooth morphologies.

#### Description

**Figure 2:**
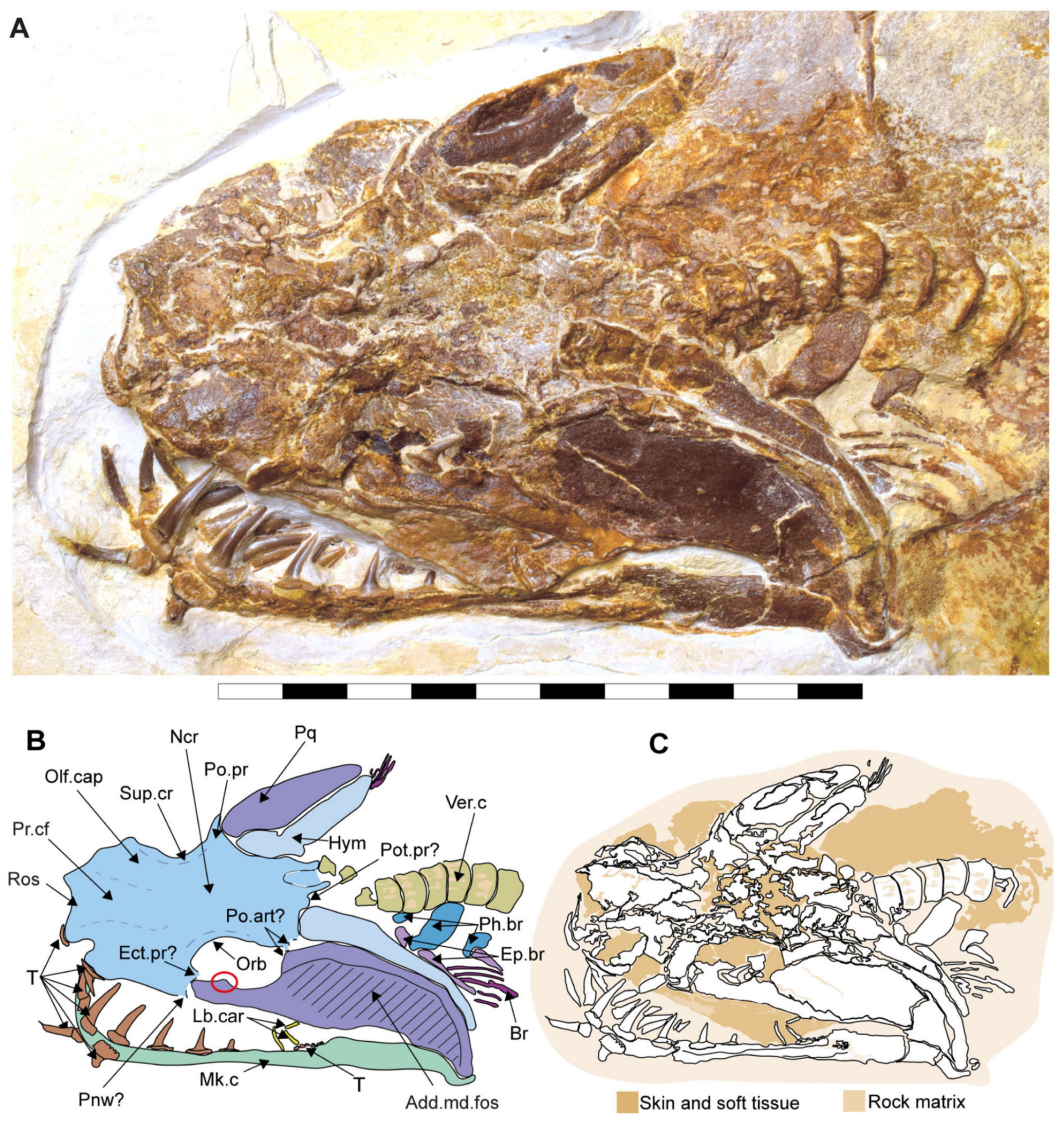
Dorsal surface of the cephalic region of †*Archaeogracilidens macer*, specimen SMNS 96844/7. **A**, Region under normal light; **B**, Interpretative drawing of the remains in the cephalic region; C, Line drawing of the remains in the cephalic region. **Abbreviations: Add.md.fos**, adductor mandibular muscle fossa; **Br**, branchial rays; **Ect. pr?**, ectethmoid process; **Ep.br**, epibranchials; **Hym**, hyomandibula; **Lb.car**, labial cartilages; Mk.c, Meckel’s cartilage; **Ncr**, neurocranium; Olf.cap, olfactory capsule; **Orb**, orbit; **Ph.br**, pharyngobranchials; Pnw?, posterior nasal wall; **Po.art?**, postorbital articulation; **Po.pr**, postorbital process; Pot.pr, postotic process; **Pq**, palatoquadrate; **Pr.cf**, precerebral fenestra; Ros, rostrum; **Sup.cr**, supraorbital crest; **Ver.c**, vertebral centra; **T**, teeth. Scale bar = 10 cm.

**Figure 3:**
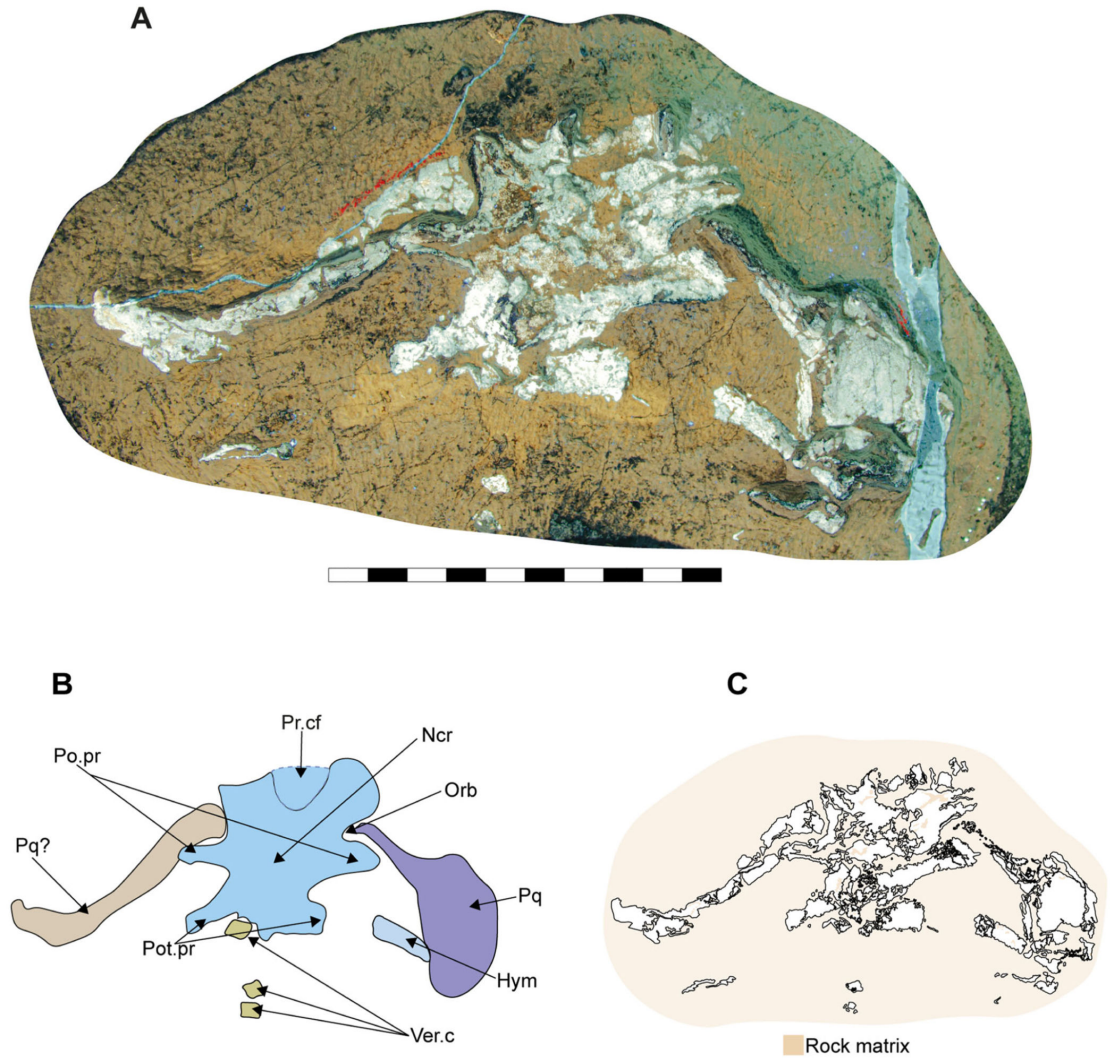
Dorsal surface of the cephalic region of †*Archaeogracilidens macer*, specimen SMNS 80142/44. **A**, Region under UV light; **B**, Interpretative drawing of the remains in the cephalic region; **C**, Line drawing of the remains in the cephalic region. **Abbreviations: Hym**, hyomandibula; **Nc**r, neurocranium; **Orb**, orbit; **Po.pr**, postorbital process; **Pot.pr**, postotic process; **Pq & Pq?**, palatoquadrate; **Pr.cf**, precerebral fenestra; **Ver.c**, vertebral centra. Scale bar = 10 cm.

The neurocranium is a box-shaped structure, with a short and narrow rostrum. Morphologically, it has wide nasal capsules, a narrow interorbital space, a broad postorbital region and a narrow occipital region (Figs 2 and 3). However, the exact extent of the nasal capsules is not apparent due to their poor preservation and deformation.

In specimen SMNS 96844/7, a protuberance resembling the ectethmoid process is observed on the left side of the neurocranium, below the nasal capsules. The palatoquadrate appears to pass through this process, supporting this interpretation (Fig. 2B, Ect. pr?). However, given the preservation state and deformation of the remains, this cannot be asserted with absolute certainty. As a result, this character is scored as missing (due to preservation loss) for these specimens (ch. 23 [?]).

The precerebral fontanelle (Figs 2B and 3B, Pr.cf) is partially preserved, with its margins suggesting it was originally located between the nasal capsules. This implies an anteriorly open neurocranium. The orbits are well separated, suggesting a morphological platybasic neurocranium, similar to the condition observed in *Notorynchus* Ayres, 1855 by Maisey (2007). However, this cannot be confirmed without direct observation of the interorbital septum (ch. 71 [?]).

The supraorbital crests are well developed, connecting anteriorly to the posterior wall of the nasal capsules and posteriorly to the postorbital process. No preorbital process was observed. The postorbital processes are located anteriorly within the otic region and project laterally. Notably, the postorbital processes in specimen SMNS 80142/44 appear larger (projecting more laterally) than those in SMNS 96844/7 (Figs 2B, and 3B, Po.pr). However, damage to these processes in SMNS 96844/7 hinders an accurate comparison.

The otico-occipital region comprises nearly half of the preserved neurocranium’s length, with the otic region being slightly larger than the orbits. The presence of foramina or structures such as the endolymphatic (parietal) fossae could not be confirmed, as they are covered by other structures or are too poorly preserved (Figs 2 and 3).

Böttcher and Duffin (2000) identified lateral otic processes in the posterior part of the otic capsules of SMNS 80142/44 (Fig. 3). The presence of lateral otic processes has been suggested for various elasmobranch taxa (Coates & Sequeira, 2001; Maisey, 1985, 1986, 2001). Schaeffer (1981, p. 54) noted their possible presence in some neoselachians (e.g. *Notorynchus* Ayres, 1855 and *Squatina* Duméril, 1805), based on figures provided by Holmgren (1941, text-figs 5 and 33). Additionally, *Squalus* Linné, 1758 presents a small otic extension in a similar position to the postotic processes (Holmgren, 1941, text-fig. 22).

Maisey and Lane (2010) noted that the lateral otic processes in neoselachians are located posteriorly in the otic capsules and contain part of the glossopharyngeal nerve. They referred to them as post-otic processes based on this association following Holmgren (1940; 1941). In contrast, the glossopharyngeal nerve passes mesially and ventrally to the lateral otic process in xenacanthiforms and †*Tamiobatis* Eastman, 1897. This structural distinction has led various researchers to consider the lateral otic and the postorbital processes as non-homologous structures (e.g. Bronson et al., 2024; Coates et al., 2017, 2018; Frey et al., 2020; Pradel et al., 2011).

Böttcher and Duffin (2000) likely used the term ‘lateral otic processes’ following the terminology used in the literature of that time (e.g. Maisey, 1985). Considering the presence of several neoselachian features in †*Archaeogracilidens*, we use the term postotic processes for these structures (Figs 2B and 3B, Pot.pr). However, due to the crushed state of the specimens, their relationship with other cranial structures, such as the glossopharyngeal canal, remains undetermined. As a result, the characters lateral otic processes (ch. 42) and postotic processes (ch. 43) were coded as missing (?) due to preservation loss in the phylogenetic analysis.

The presence of ventrally oriented teeth at the level of the rostrum, possibly belonging to the palatoquadrate, along with the extension of the Meckel’s cartilage, suggest that the symphysis of the jaw cartilages was closely below the rostrum (Figs 2B).

The palatoquadrate is cleaver-shaped, with a narrower palatine portion and a broad quadrate region. In specimen SMNS 96844/7, the palatoquadrate appears displaced to the side of the neurocranium, resting on its medial face, with some areas covered by skin and others like the central part covered by dental remains and left unprepared, limiting further observations. A small articulation process surrounded by bits of cartilage is visible in the palatine region (Fig. 2B, marked with a red circle). This may indicate a palatine-cranial articulation, though this remains speculative. In the remaining specimens, this area also was too poorly preserved to make accurate observations and comparisons.

The most prominent feature of the palatoquadrate is its large adductor fossa, which extends across most of the quadrate portion (Fig. 2B, Add.md.fos). This fossa is dorsally delimitated by a well-defined quadrate ridge, which broadens and become relatively concave near the post-orbital process. This variation in width and depth suggests the presence of a synovial cavity at this position.

Meckel’s cartilages are notably broad at their middle portion, featuring a deep symphyseal edge aligned to the central axis of the neurocranium. The posterior margin of the Meckel cartilages, forming the jaw joint, extends externally (Figs 2B, 5B).

The hyomandibula is elongated, positioned posteriorly to the palatoquadrate. Its proximal end reaches the otic capsule, while its distal end extends to the posterior end of the palatoquadrate and Meckel’s cartilages (Fig. 2B, Hym).

**Figure 4:**
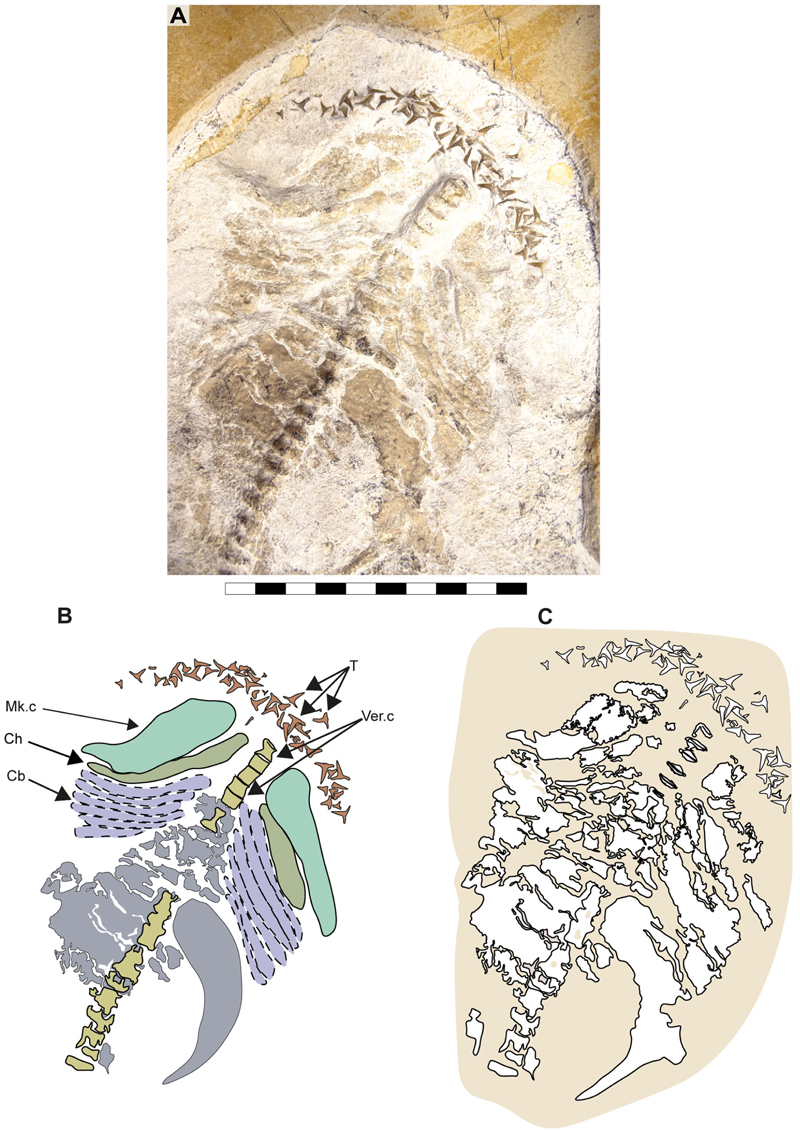
Ventral surface of the cephalic region of †*Archaeogracilidens macer*, specimen SMNS 80142/44. **A**, Region under normal light; **B**, Interpretative drawing of the remains in the cephalic region; **C**,. Line drawing of the remains in the cephalic region. **Abbreviations: Cb**, ceratobranchials; **Ch**, ceratohyal; **Mk.c**, Meckel’s cartilage; **T**, teeth; **Ver.c**, vertebral centra. Scale bar = 5 cm.

**Figure 5:**
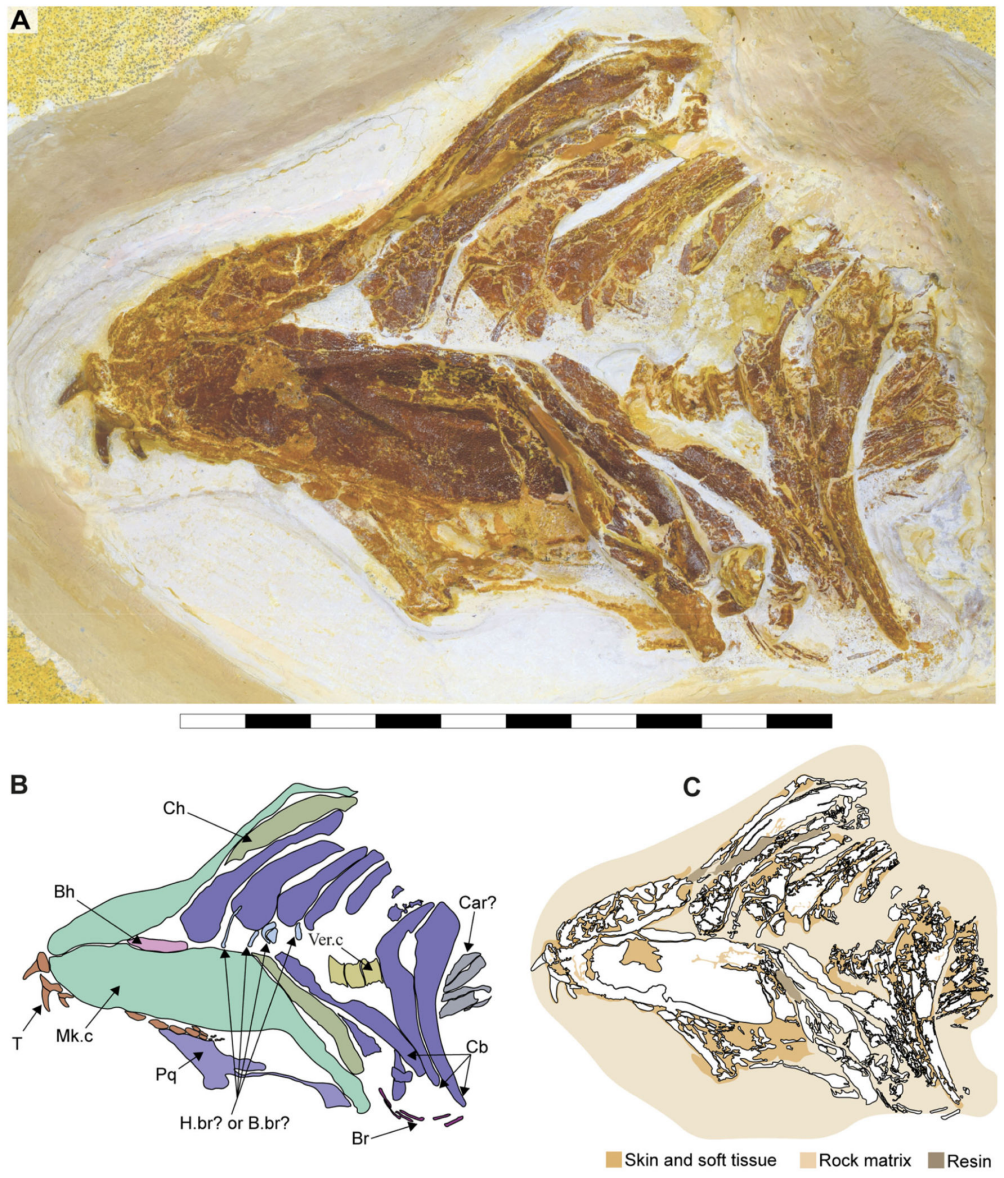
Ventral surface of the cephalic region of †*Archaeogracilidens macer*, specimen SMNS 96844/7. **A**, Region under normal light; **B**, Interpretative drawing of the remains in the cephalic region; **C**, Line drawing of the remains in the cephalic region. **Abbreviations: B.br?**, basibranchial; **Bh**, basihyal; **Br**, branchial rays; **Car**, unidentified cartilage; **Cb**, ceratobranchials; **Ch**, ceratohyal**; H.br**?, hypobranchials; **Mk.c**, Meckel’s cartilage; **Pq**, palatoquadrate; **T**, teeth; **Ver.c**, vertebral centra. Scale bar = 5 cm.

**Figure 6:**
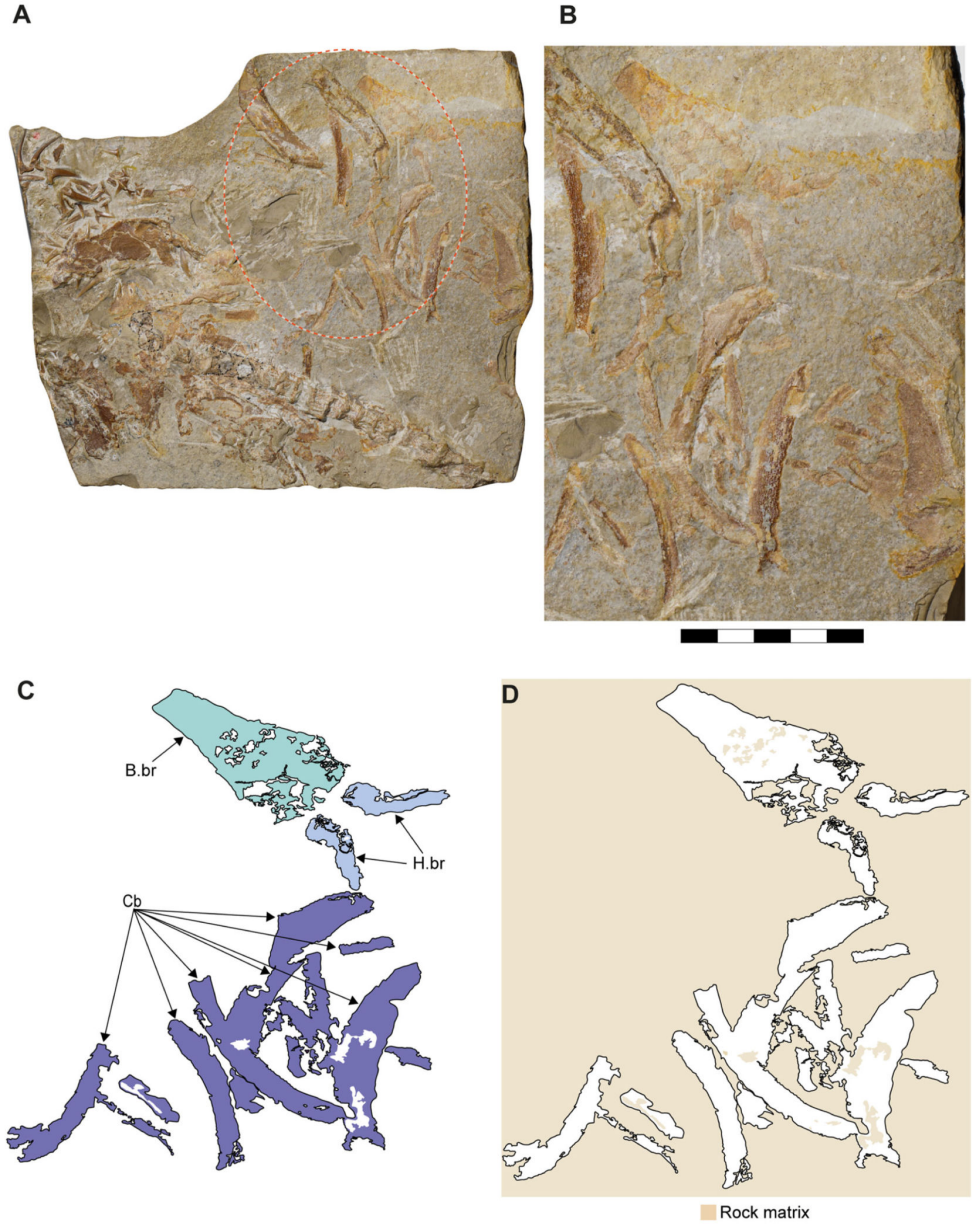
Disarticulated specimen of †*Archaeogracilidens macer* SNSB-BSPG AS VII 647. **A**, Under normal light; **B**, Close up of the region marked by a red ellipse in A; **C**, Interpretative drawing of the branchial remains; **D**, Line drawing of the branchial remains. **Abbreviations: B.br**, basibranchial; **Cb**, ceratobranchials; **H.br**, hypobranchials. Scale bar = 5 cm.

The ceratobranchials have a broad proximal portion but taper towards their distal end (Fig. 4B, Cb). Several ceratobranchials are preserved in the examined specimens (Figs 4B, 5B, 6B, Cb). However, their precise number cannot be estimated with certainty. In SMNS 80142/44, these elements are covered by soft tissue, complicating the identification of their edges (Fig. 4B). In SNSB-BSPG AS VII 647 and SMNS 96844/7, disarticulation and fragmentation hinder the estimation their number (Figs 5B, 6B). The ceratobranchials present a simple morphology, without any significant processes, notches or crests.

A series of small cartilages closely associated with the ceratobranchials were observed in SMNS 96844/7. Their position suggests that they likely represent parts of the hypobranchials and anterior basibranchial series. However, these elements were not identified in other specimens included in this study, and consequently no further comparative observations were made (Fig. 5B, H.br?, B.Br?).

Two hypobranchials, probably the last pair of the series, are present in SNSB-BSPG AS VII 647 (Fig. 6B, H.br). These structures project medially and articulate with the basibranchial, which exhibits a wide anterior region as an articulation point for them. The basibranchial distal portion is narrow and terminates abruptly, with no additional cartilages near it.

The revision and comparison of the dental arrangement in SMNS 96844/7, alongside teeth preserved in SMNS 80142/44 and SNSB-BSPG AS VII 647, high-lighted key taxonomic considerations. Böttcher and Duffin (2000) suggested that the degree of torsion in the central cusp could serve as a diagnostic feature at the species level. However, their observations rely heavily on isolated teeth (SMNS 3695/6, SMNS 80431/36, SMNS 80144/6–12), which may limit the applicability of this feature when assessing articulated specimens. In articulated specimens such as the holotype of †’*Sphenodus nitidus*’ Wagner, 1862 and SMNS 80142/44 and SMNS 96844/7, direct observations of this feature are challenging, as either only one crown face (labial or lingual) is accessible with the other being embedded in the matrix, or obscured by other skeletal elements. Additionally, as previously noted by Böttcher and Duffin (2000), cusp torsion appears to vary depending on the tooth position within the jaw. Some lateral and posterior teeth of †’*Sphenodus nitidus*’ (Wagner, 1862, p. 290, pl. 1, fig. 4) present little or no torsion as seen in specimen SNSB-BSPG AS VII 647. These teeth bear strong resemblance with those described as †’*Sphenodus*’ *macer* by Quenstedt (1851) with reduced torsion, smaller cusps and well-developed cutting edges on both sides of the cusp. This variation presents a significant taxonomic challenge, as the dentition of †’*Sphenodus nitidus*’ encompasses diagnostic features of †’*Sphenodus*’ *macer*. Consequently, cusp torsion may not be a reliable criterion for species delimitation, as it likely reflects positional variation within the jaws rather than fundamental differences between species.

**Figure 7:**
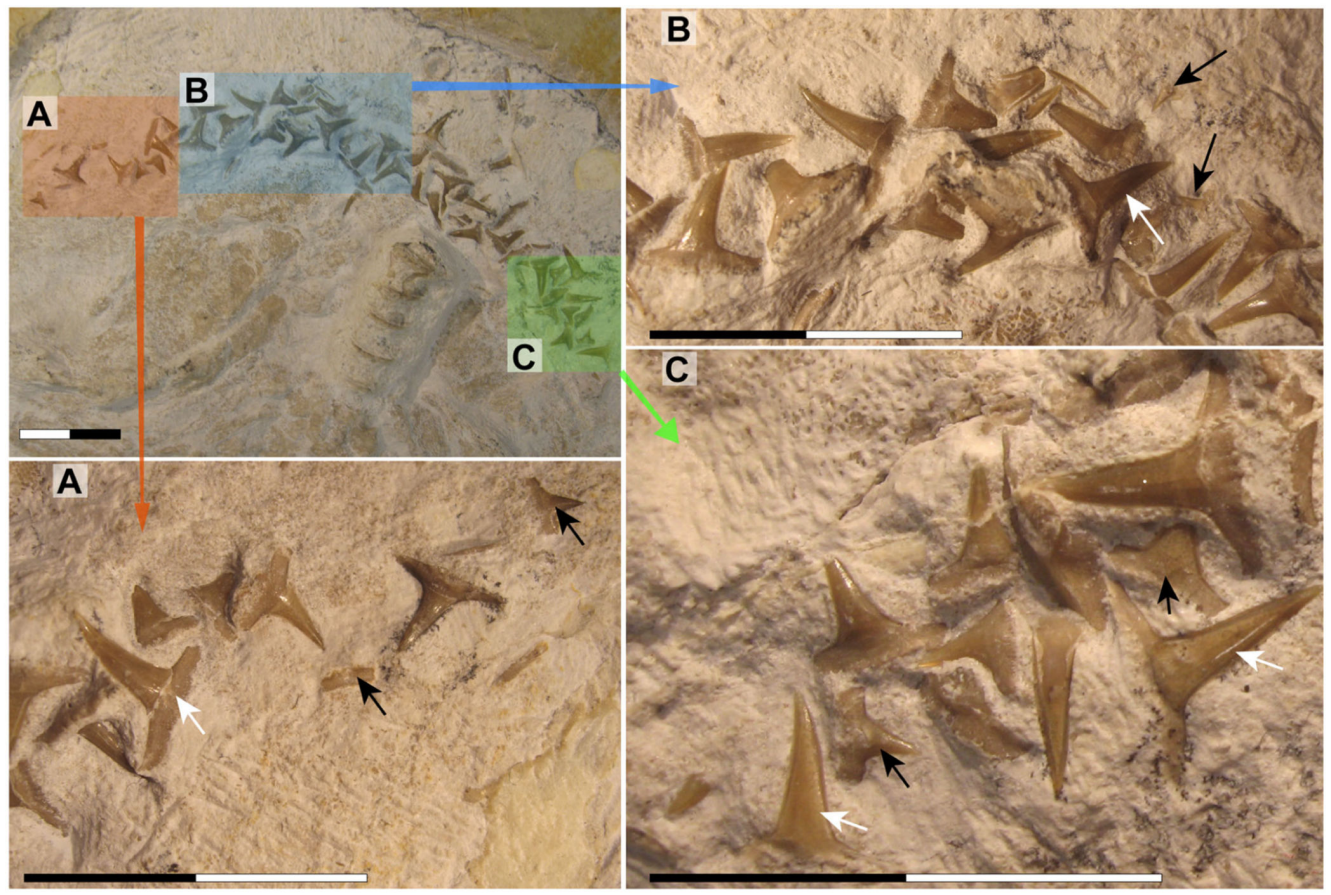
Dental regions of †*Archaeogracilidens macer*, specimen SMNS 80142/44. **A**, Right lateral posterior portion; **B**, Right anterior lateral portion; **C**, Left lateral posterior portion. Black arrows indicate teeth of smaller size. White arrows indicate teeth of larger size. Scale bars = 2 cm.

Böttcher and Duffin (2000) proposed a dental reconstruction in which differences in crown height between anterior teeth and posterior teeth could serve as a diagnostic feature to distinguish between ‘*S*.’ *macer* Quenstedt, 1851, ‘*S*.’ *nitidus* Wagner, 1862, and ‘*S*.’ *longidens* (Agassiz, 1843). This reconstruction is based on the assumption that the dentition of the specimen SMNS 80142/44 presents minimal disturbance, allowing tooth jaw origin (upper or lower) to be inferred based on the orientation of the labial and lingual tooth surfaces and position assigned based on tooth position in relation to the Meckel’s cartilages. While this approach is reasonable, the teeth in SMNS 80142/44 appear noticeably disarranged (Figs 4B and 7), with teeth of varying sizes positioned inconsistently. This leads to sections with tooth sizes that do not fully conform to the proposed reconstruction (see Böttcher & Duffin, 2000, text-fig. 9).

Moreover, the inclusion of an ‘intermediate tooth’ within the reconstruction introduces additional uncertainty, as there is no definitive evidence for its presence. In light of these uncertainties, the Böttcher and Duffin (2000) reconstruction should be considered with caution.

Considering these uncertainties, we did not pursue further dental comparisons in the present study, nor did we attempt any possible species distinctions based on dental features.

**Figure 8:**
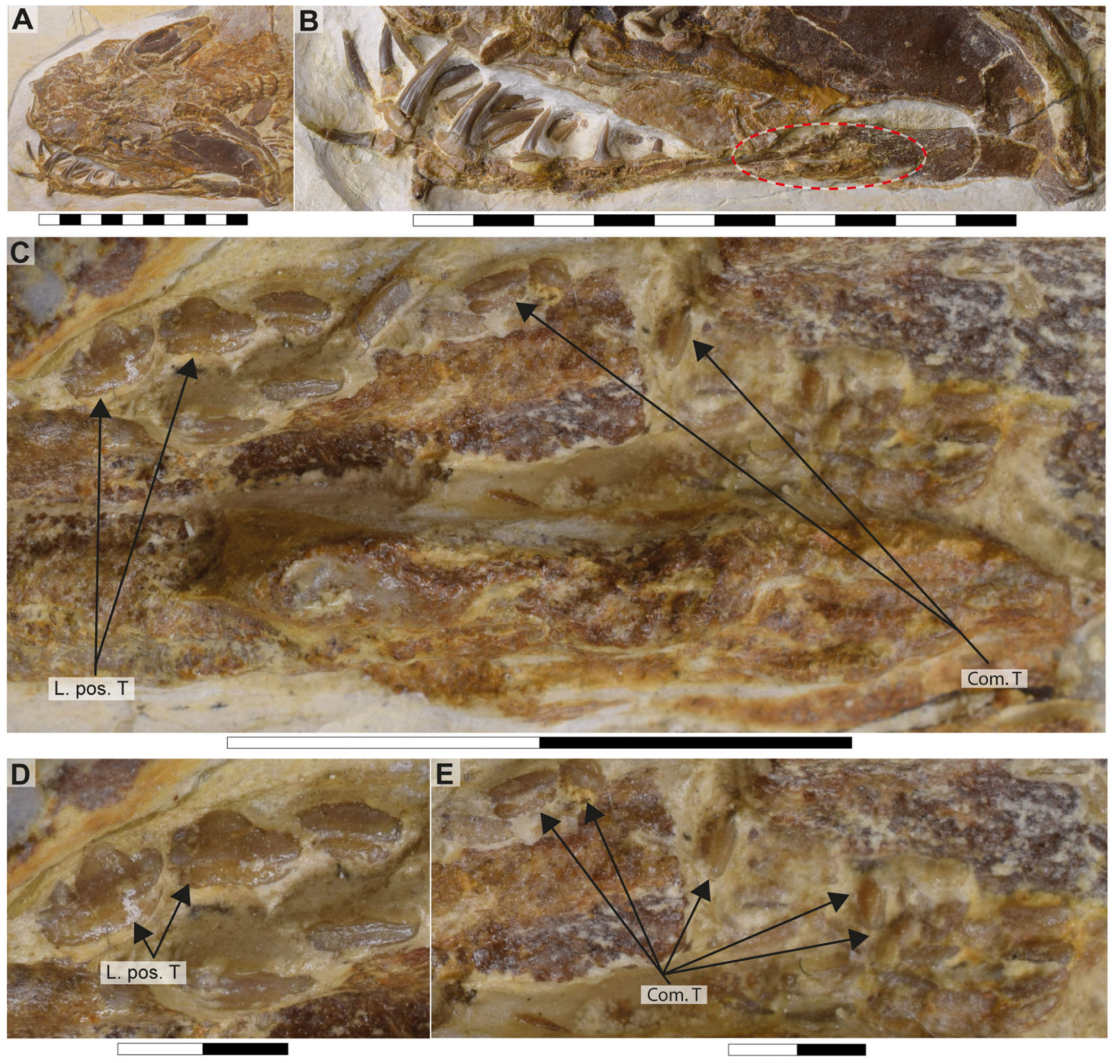
Lower jaw dentition of †*Archaeogracilidens macer*, specimen SMNS 96844/7. **A**, Dorsal surface of the cephalic region. **B**, Expanded view of the lower jaw. **C**, Close up to the area marked with a red circle, showing the posterior and commissural teeth; **D**, Posterior teeth; E, Commissural teeth. **Abbreviations: Com.T**, Commissural teeth; **L.pos.T**, lateral posterior teeth. Scale = bars 10 cm in A, B; 1 cm in C; 2 mm in D, E.

The lower jaw of †*Archaeogracilidens* exhibits distinct tooth morphologies, including anterior, lateral, posterior and commissural teeth (Fig. 8). The anterior and lateral teeth are characterized by high, slender, and sharply accentuated central cusps without lateral cusplets, but with laterally expanded heels, indicative of a tearing dentition type (Cappetta, 2012). The crown height gradually decreases distally (Fig. 8B).

**Figure 9:**
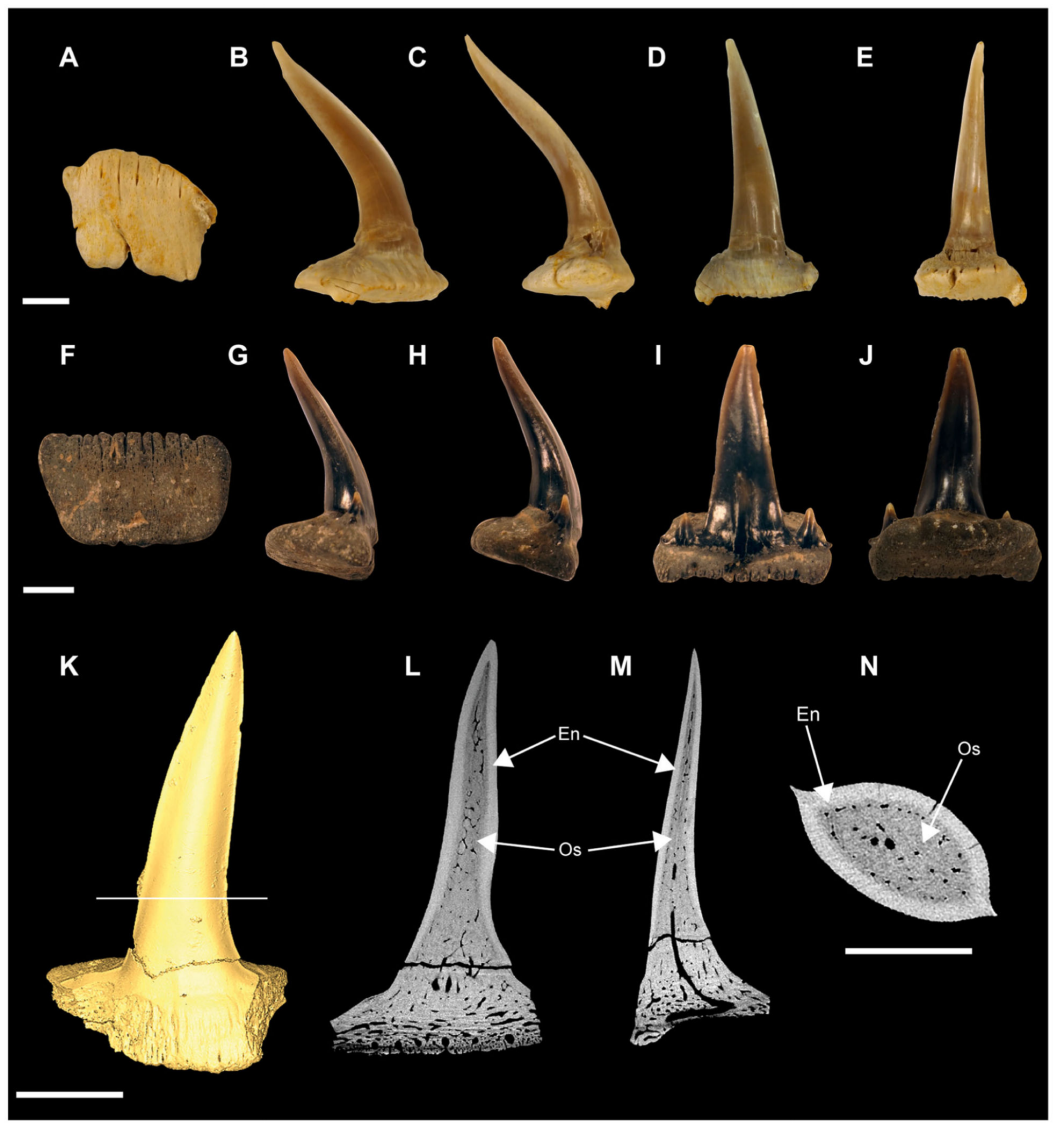
Isolated teeth of †*Archaeogracilidens macer*. **A–E**, specimen SMNS 80142/6 presenting the morphology of the anterior teeth: **A**, Basal view; **B**, Lateral view (left side); **C**, Lateral view (right side); **D**, Labial view; **E**, Lingual view. **F–J**, specimen SMNS 86244 presenting the morphology of the lateral posterior teeth: **F**, Basal view; **G**, Lateral view (left side); **H**, Lateral view (right side); **I**, Labial view; **J**, Lingual view. **K–N**, Micro-CT images of specimen EMRG-Chond-T-78: **K**, Isosurface; **L**, Section through the frontal plane; **M**, Section through the sagittal plane; **N**, Section through the axial plane. **Abbreviations: En**, enameloid; **Os**, osteodentine. Scale bars 2.5 mm in A–J; 1mm in K–N.

The anterior and anterolateral teeth display a sigmoidal curvature in profile view and distinctly asymmetric cutting edges (Fig. 9B–E). As the dentition progresses posteriorly the curvature gradually diminishes, accompanied by a reduction in cusp height. Simultaneously, the cusps become increasingly inclined distally and the cutting edges become symmetric. Additionally, the tooth root of the lateral teeth is wider than that of the anterior teeth.

Posterior teeth display a single pair of small lateral cusplets adjacent to the main cusp (Figs 8C–D, 9G–J). Commissural teeth lack a central cusp, displaying a molariform morphology with absent cusps (Fig. 8E).

The CT-scanned tooth of †*Archaeogracilidens* displays a thick layer of enameloid and a core of osteodentine, fully traversed by dentinal osteons (Fig. 9L–M) with no hollow pulp cavity. This histological structure corresponds to the ostedont histotype (Hättig et al., 2019; Jambura et al., 2019, 2020).

Tooth ornamentation is limited to short, apicobasally oriented striations along the basal margin of the lingual crown face. The root is nearly perpendicular to the crown forming an almost right basal angle. The root becomes progressively lower and lingually more expanded distally. The basal root surface is flat, wide and expands laterally and lingually. Nutritive grooves are present along the labial margin, and sometimes lingually as well (Fig. 9A, F). The root vascularization follows the non-compact pseudopolyaulacorhize-type *sensu* Cappetta (2012).

**Figure 10:**
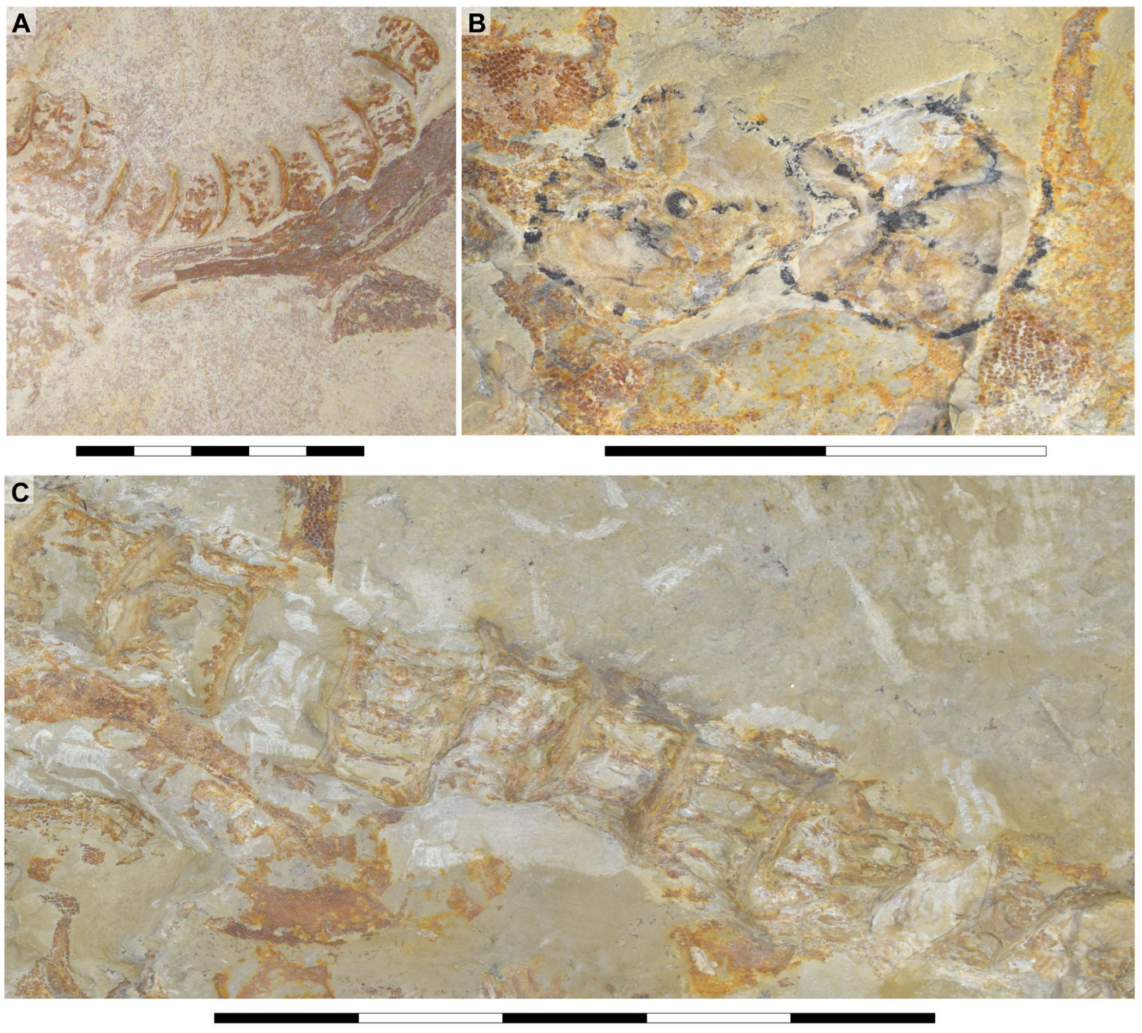
Vertebral centra of †*Archaeogracilidens macer*. **A**, Anterior portion of the vertebral column in specimen SMNS 96844/7; **B**, Broken vertebra of specimen SNSB-BSPG AS VII 647 showing the internal calcification pattern of the vertebral centra; **C**, Unbroken vertebral centra of specimen SNSB-BSPG AS VII 647. Scale bars = 5 cm in A, C; 2 cm in B.

†*Archaeogracilidens macer* presents well mineralized vertebral centra (Fig. 10), with lateral lamellae suggesting an asterospondylic mineralization pattern throughout its body (Fig. 10B).

In specimen SMNS 80142/44, 55 vertebral centra are recognizable, although portions of the axial skeleton are obscured by branchial elements and mineralized soft tissue, complicating establishing the exact count. The average anteroposterior length of the vertebral centra is 5.85 mm. Specimen SMNS 96844/7 preserves 104 recognizable centra, averaging 6.4 mm in length. Overall, the average length of the vertebral centra for the species is of 6.3 mm (see Supplemental material).

**Figure 11:**
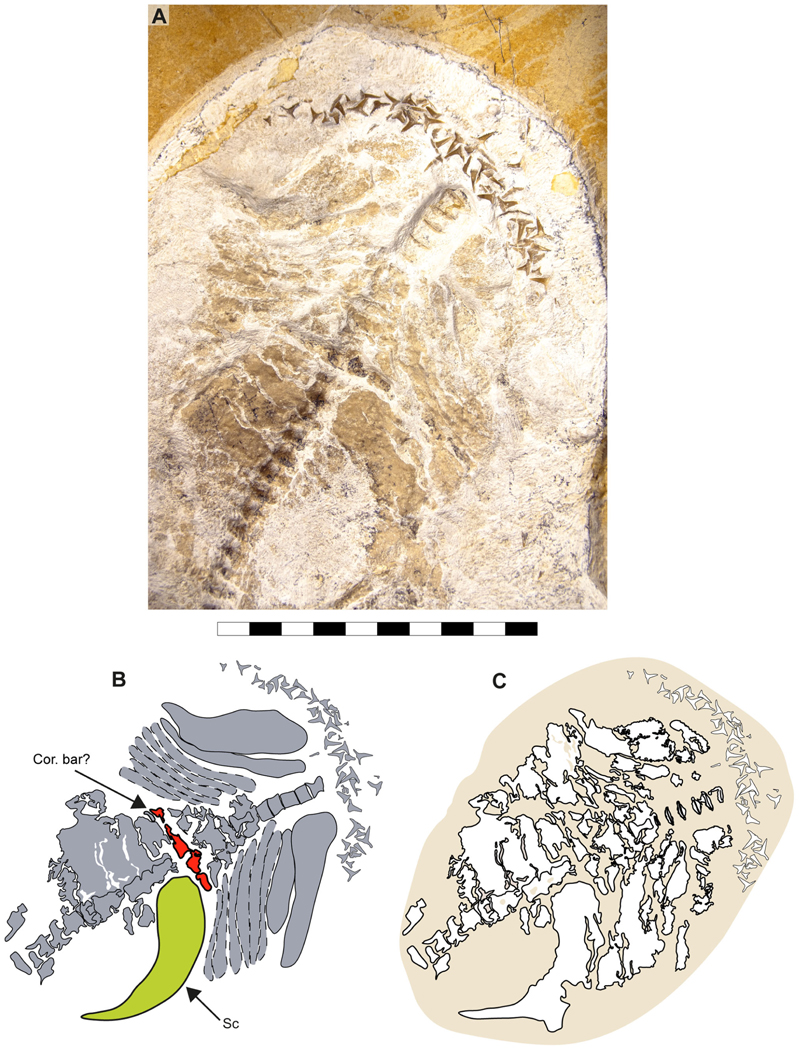
Ventral view of anterior region of †*Archaeogracilidens macer*, specimen SMNS 80142/44. **A**, Region under normal light; **B**, Interpretative drawing of the cartilage remains in the region; **C**, Line drawing of the cartilage remains in the region. **Abbreviations: Cor.bar?**, coracoid bar; **Sc**, scapulacoracoid. Scale bar = 5 cm.

**Figure 12:**
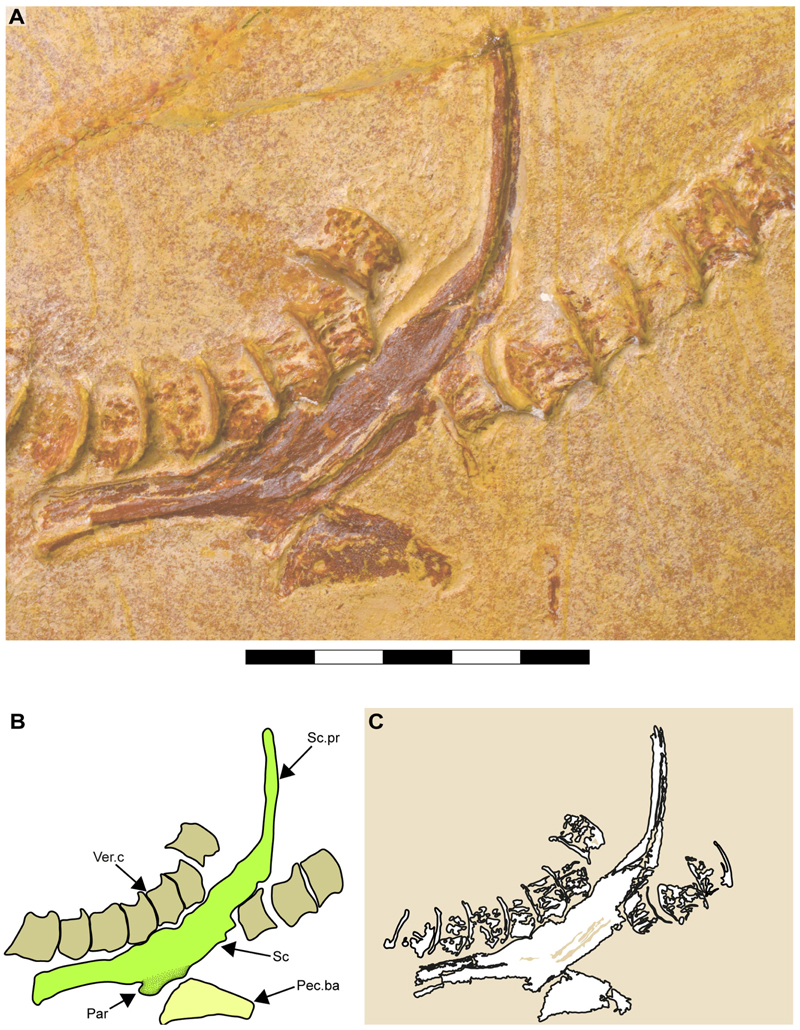
Lateral view of the pectoral region of †*Archaeogracilidens macer*, specimen SMNS 96844/7. **A**, Region under normal light; **B**, Interpretative drawing of the pectoral remains; **C**, Line drawing of the pectoral remains. Abbreviations: Par, pectoral articulation surface; **Pec.ba**, pectoral basal element; **Sc**, scapulacoracoid; **Sc.pr**, scapular process; **Ver.c**, vertebral centra. Scale bar = 5 cm.

In both specimens, only one half of the pectoral girdle is preserved (left side in SMNS 96844/7 and left side in SMNS 80142/44). Their overall morphology suggests a ‘U’-shaped scapulacoracoid. The dorsally projected scapular process is fused to the scapula, with no evidence of a scapular fossa (Figs 11, 12).

Böttcher and Duffin (2000) identified a transversal ridge with cartilage fragments and scales as part of the coracoid bar in specimen SMNS 80142/44 (Fig. 11B: Cor.bar?). However, the position and association of the scapular cartilage with this ‘coracoid bar’ is unusual. The nearly intact left scapular cartilage appears to rest on its anterior surface, with the posterior surface facing the observer. This orientation suggests a post-mortem rotation of the coracoid bar. The fusion between scapular and coracoid cartilages would also shift this element, making its anterior and posterior surfaces visible, depending on the direction of the scapular cartilage. In neoselachians with a fused pectoral girdle, the anterior and posterior faces of the coracoid bar progress smoothly into the lower part of the scapular cartilage and conserve the width of the lower portion. In specimen SMNS 80142/44, however, this crest is distinctly separated from the scapular cartilage and is considerably narrower than the lower part of the scapular cartilage. While taphonomic processes may account for the breakage of the pectoral girdle, explaining the peculiar shift of the parts, the clean separation between this crest and the pectoral element makes this scenario less likely.

In specimen SMNS 96844/7, only the left half of the pectoral girdle is preserved. Its lower part shows a clean separation, with no closely associated cartilage fragments, possibly indicating a loose connection between the halves of the pectoral girdle (Fig. 12). However, since neither specimen preserves both halves, it remains unclear whether they were fused as in most neoselachians or just articulated as in Hexanchiformes. Consequently, this character was coded as unknown for †*Archaeogracilidens macer* (ch. 117 [?]).

**Figure 13:**
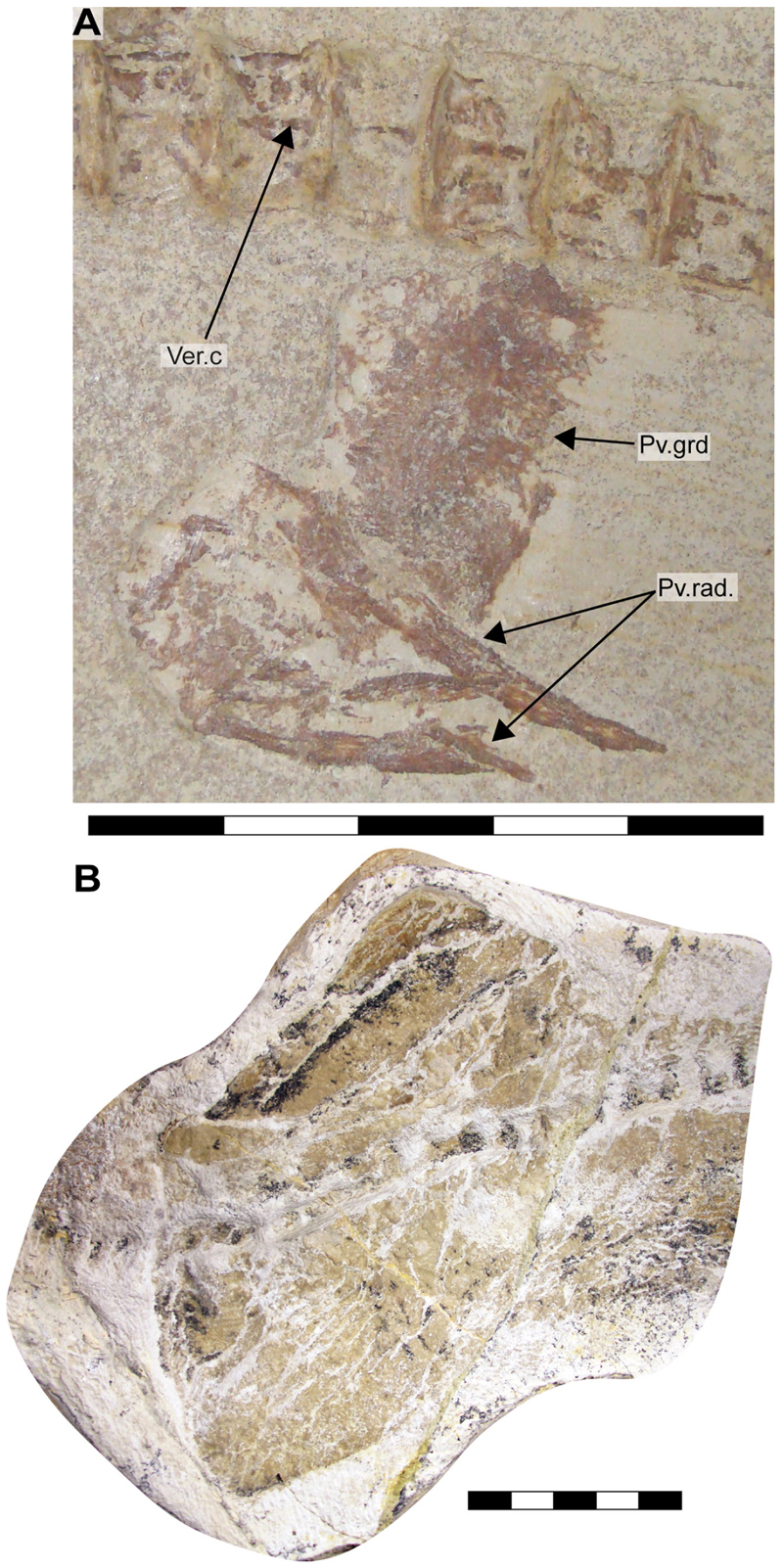
Pelvic girdle of †*Archaeogracilidens macer*. **A**, specimen SMNS 96844/7 under normal light. **B**, specimen SMNS 80142/ 44 under normal light. **Abbreviations: Pv.grd**, pelvic girdle; **Pv.rad**, pelvic radials; **Ver.c**, vertebral centra. Scale bars = 5 cm in A; 10 cm in B.

Only one of the basal pectoral cartilages is preserved in specimen SMNS 96844/7, exhibiting a trapezoidal shape (Fig. 12B, Pec. Ba). The shape of the pectoral and pelvic fins is partially visible in SMNS 80142/44. Both paired fins are roughly triangular in ventral view, with fin tips angled slightly posteriorly (Fig. 1). Skin folds and some of the underlying structures are discernible. However, it remains ambiguous whether these structures represent radials or impressions of the ceratotrichia (Fig. 13B).

In specimen SMNS 96844/7, the pelvic girdle is located anteriorly to the dorsal fin. Its crushed condition suggests displacement, making its original position difficult to determine. Despite the crushed state of the pelvic girdle, some features can still be identified, including a puboischiadic bar articulating with likely the first pelvic radial. Due the preservation and position, it is impossible to determine whether the pelvic girdle halves were fused (Fig. 13A).

In specimen SMNS 80142/44, the pelvic girdle is covered by tissue, making its exact position with respect to the dorsal or anal elements indeterminable, as these unpaired elements are additionally not preserved (Fig. 13B).

In both specimens SMNS 96844/7 and SMNS 80142/44, no evidence of intromittent organs associated with the pelvic girdles is present. This confirms that both specimens are female.

A single dorsal fin is present, with no evidence of a second one (Fig. 1A, Df). The remains of the vertebral column and the imprinting ceratotrichia obscure the presence of basal cartilages and radial elements, but overall, it seems that the dorsal fin appears aplesodic.

Böttcher and Duffin (2000, text-fig. 3) interpreted a long, scale-covered ridge extending ventrally from their estimated 52nd to 61st vertebral centra as the anal fin base in SMNS 80142/44. However, the exact nature of this ridge remains uncertain. Comparison with SMNS 96844/7 suggests it might be a basal element of an unpaired fin (dorsal or anal), or might just represent a skin fold.

### Phylogenetic results

The literature review and the morphological examination of the skeletal remains in this study resulted in the assembly of a data matrix comprising 211 morphological characters scored across 93 terminal taxa. A comprehensive sample of non-neoselachian and neoselachian chondrichthyans was included to further test the systematic position of †*Archaeogracilidens* and to provide a more complete understanding for morphological character polarization (see Supplemental material). In this analysis, †*Pucapampella* Janvier and Suárez-Riglos, 1986 was used as the tree root.

The Lewis/Mkv model was the best-fitting model for the data matrix. The topology estimated under no implied weight parsimony is the most similar to the Mkv tree, with a score of 5 SPR moves and a similarity score of 0.9444. Both topologies are presented, focusing on the relationships of †*Archaeogracilidens* (for the full phylogenetic trees and results of all the comparisons see Supplemental material).

**Figure 15:**
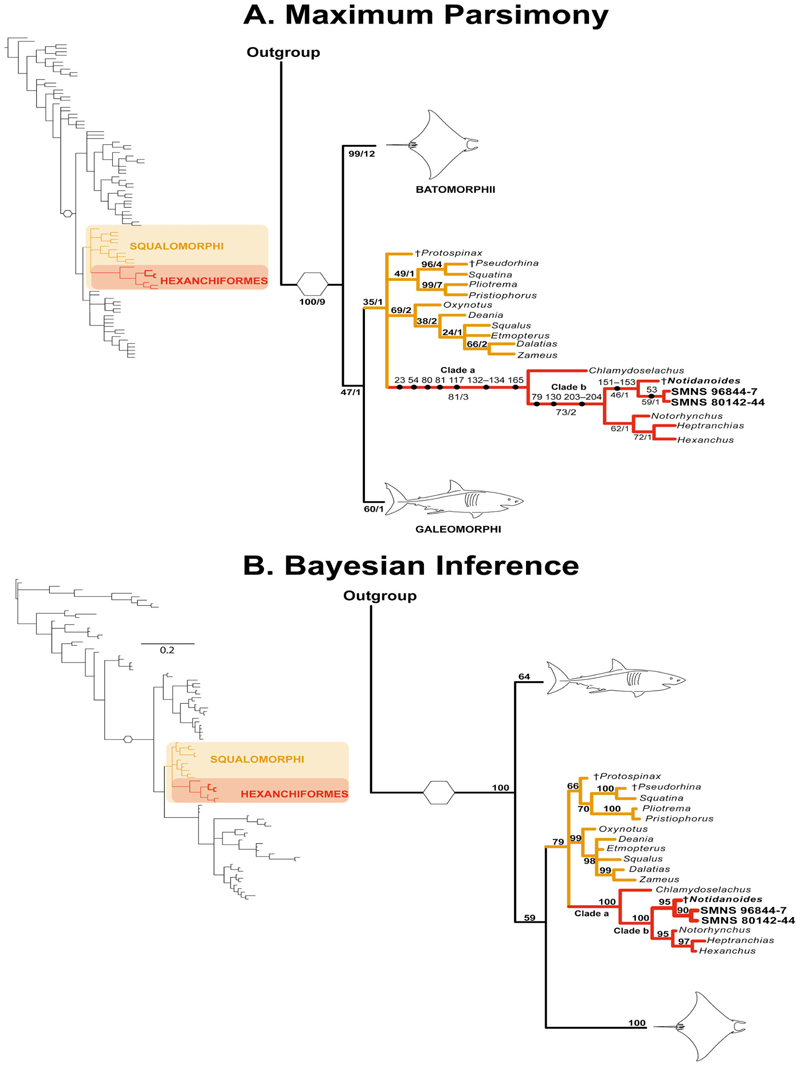
Phylogenetic trees estimated in the present analysis. **A**, Compact strict consensus tree of the 1024 MPTs. Numbers on branches accompanying the points represent the characters and character states supporting the clades of interest. Numbers below branches indicate the jackknife scores and Bremer support values for the clades (Jk/Br); **B**, Compact majority rule consensus tree estimated from the trees with the best posterior probability estimated in the Bayesian inference analysis. Numbers below branches indicate the posterior probability (PP). Clade of interest marked in red, catalogue number of both holomorphic specimen of †*Archaeogracilidens* in bold. Clade a: Hexanchiformes, Clade b: [[†*Notidanoides* + †*Archaeogracilidens*] + Hexanchidae].

The search protocol for the parsimony analyses resulted in 1024 most parsimonious trees (MPTs) of 737 evolutionary steps with a consistency index of 0.43 and retention index of 0.83. The phylogenetic hypotheses estimated from both parsimony and Bayesian inference analyses align with previous studies, placing †*Archaeogracilidens* (†*Sphenodus sensu* Quenstedt, 1865) within the Neoselachii (see Supplemental material). Despite topological discrepancies between parsimony and Bayesian frameworks, both analyses converge on the placement of †*Archaeogracilidens* within Hexanchiformes (Fig. 15). In this context, †*Archaeogracilidens* is recovered as sister group to †*Notidanoides* Maisey, 1986. This relationship is well supported by morphological characters, with the parsimony analysis yielding a jackknife score (Jk) of 81 for Hexanchiformes and 73 for the sister group relationship of [[†*Notidanoides* + †*Archaeogracilidens*] + Hexanchidae], whereas the Bayesian analysis recovered these relationships with a posterior probability (Pp) of 100% for both clades (Fig. 15, Clades a and b).

In the parsimony analysis, the Hexanchiformes clade is supported by several characters, including the mineralization of the antorbital process of the ectethmoid process (ch. 23[1]), the presence of an orbital artery foramen (ch. 54[0]), a deep groove on the quadrate portion of the palatoquadrate for the insertion of the quadratomandibularis adductor muscle (ch. 80[1]), the presence of a large otic process in the palatoquadrate (ch. 81[1]), unfused ventral antimeres of the scapulocoracoid (ch. 117[1]), a reduced but broad propterygium, lacking an anterior extension and not articulating directly with radials (chs 132[1], 133[3], 134[1]), and a single dorsal fin (ch. 165[0]) (Fig. 15A: Clade a).

The clade comprising Hexanchidae, †*Notidanoides* Maisey, 1986 and †*Archaeogracilidens* is supported by the presence of an amphistylic jaw articulation (*sensu* Maisey, 2008) (ch. 79[2]), a proximally segmented metapterygium with a broad distal end for the articulation of pectoral radials (ch. 130[5]), and the presence of a tooth root with basal furrows (ch. 203[1]). A comprehensive character list, descriptions, and character state reconstructions can be found in the Supplemental material, as certain characters were coded as missing [?] for †*Archaeogracilidens*.

Both analyses consistently place †*Archaeogracilidens* in a sister-group relationship with †*Notidanoides* Maisey, 1986 (Fig. 15). This relationship is better supported by the Bayesian analysis, which provides a Pp of 95% compared to the parsimony analysis, which estimated a Jk score of 46. In the parsimony analysis, this relationship is supported by the presence of well-mineralized vertebral centra, with primary and secondary mineralization of the entire vertebral centrum (chs 151[1], 152[1] and 153[1]).

## Discussion

### †*Archaeogracilidens* as a galeomorph

A possible galeomorph affinity, with close relationships to lamniform sharks, was suggested for †’*Sphenodus*’ in the past, mainly based on similarities in tooth crown morphologies (e.g. de Beaumont, 1960; Carroll, 1988; Glickman, 1957; Woodward, 1889). Maisey et al. (2004) supported this interpretation, mentioning features like the presence of asterospondylous vertebrae, distinct anteroposterior heterodonty in crown height, basibranchial morphology (notably, the presence of a profoundly notched fifth ceratobranchial and the possible distinct median notch on the anterior surface of the basibranchial for the articulation of a small anterior basibranchial), and the presence of an intermediate ‘eye’ tooth (based on the dental interpretation of Böttcher & Duffin, 2000).

However, several issues arise regarding these characters. The presence of asterospondylous centra is of ambiguous relevance (see also Ridewood & MacBride, 1921). The asterospondylous vertebral centrum type is based on Hasse’s (1879, p. 48) description. This figure corresponds to *Heptranchias* Rafinesque, 1810, which displays six radiating lamellae (Ridewood & MacBride, 1921, p. 348, text-fig. 5). However, Hasse (1878, p. 41 ff.) identified *Heptranchias* as belonging to the ‘diplospondylic-plagiostomi’, a group that is not included in his ‘asterospondylic-plagiostomi’. Additionally, according to Ridewood and MacBride (1921), the calcification patterns vary depending on the body region. An asterospondylous vertebral centrum pattern has been reported in various synechodontiform sharks, including †*Synechodus dubrisiensis* (Mackie, 1863) (NHMUK PVP 49032), †*Palidiplospinax ennis-killeni* (Duffin & Ward, 1993) (NHMUK PVP 3189) and †*Paraorthacodus jurensis* (Schweizer, 1964) (SMNS 88987/1) (see also Klug & Kriwet, 2008; Klug et al., 2009), as well as other taxa like †*Pseudonotidanus politus* (Thies, 1992) (see Underwood & Ward, 2004), and radial lamellae, an indicative feature of the asterospondylous-like pattern (Ridewood & MacBride, 1921, pp. 337–347; Maisey & Wolfram, 1984; Newbrey et al., 2013), have been reported in the early Late Cretaceous hexanchiform †*Hexanchus gracilis* (Davis, 1887) (see Cappetta, 1980) and in †*Notidanoides muensteri* (Agassiz, 1843) (GPIT 1210-3) (Maisey, 1986), suggesting that vertebral calcification patterns have uncertain phylogenetic significance.

The notched anterior basibranchial surface, suggested for articulation with a small anterior basibranchial, is also of questionable significance. This feature occurs in some galeomorphs (e.g. *Heterodontus* de Blainville, 1816 and *Rhizoprionodon* Whitley, 1929), but is absent in others (e.g. *Hemiscyllium* Müller & Henle, 1838). It is unclear whether the presence of this feature is plesiomorphic for Galeomorphii or results from independent evolutionary acquisitions. Similar small anterior basibranchial cartilages are found in some squalomorphs (e.g. *Squalus* Linné, 1758, *Squatina* Duméril, 1805 and hexanchiform sharks) (see Shirai, 1992a, pls 30–32, 1992b, fig. 2), complicating its utility for inferring relationships with galeomorph sharks.

The interpretation of the fifth ceratobranchial’s deep posterior notch and slender curved distal extremity in †*Archaeogracilidens* (Maisey et al., 2004, text-figs 15, 16) is based on a poorly preserved specimen (SNSB-BSPG AS VII 647) (Fig. 6). While the ceratobranchial element is identifiable in this specimen, its state of pres-ervation makes detailed characterization of the notch impossible. Better-preserved elements in specimen SMNS 96844/7 (Fig. 5) show no evidence of a deep notch.

The presence of an intermediate ‘eye’ tooth in specimen SMNS 80142/44, considered a typical feature of macrophagous lamniform sharks (Shimada, 2002), was questioned by Klug et al. (2009) who suggested it represents a parasymphysial tooth instead. Given the disarticulated state of the dentition in specimen SMNS 80142/44, this assessment seems more plausible, as teeth appear mixed post-mortem, indicating that these smaller teeth were displaced from other areas of the dentition.

The presence of an anteroposterior gradient of monognathic heterodonty, expressed mostly in the crown height, most likely results from the striking central cusp in the anterior and lateral teeth present in this species. However, revisions of the specimens suggest a more complex dental pattern, with changes in cusp height, curvature, inclination, and the development of lateral cusplets in posterior teeth, and reduced, flat crowns in commissural teeth.

The present analysis did, however, recover osteodont tooth crown histology as a shared feature of †*Archaeogracilidens* with lamniform sharks. This histotype has been proposed as a synapomorphy for lamniforms (Jambura *et al*. 2019, 2020, 2023; Moyer et al., 2015; Schnetz et al., 2016). Nevertheless, the phylogenetic analysis and revision of characters did not recover any additional traits supporting a closer relationship with lamniforms. Consequently, the reduction of orthodentine (i.e. exclusive presence of osteodentine) is interpreted here as an independent gain for both the lamniform shark taxa included in the analysis and †*Archaeogracilidens*.

The similarities of tooth microstructures between lamniforms and †*Archaeogracilidens* suggest phylogenetic uncertainty that warrants further investigation, considering not just the overall similarity between this tissue organization, but also its development and histology (e.g. Moyer et al., 2015; Jambura et al., 2018). Additionally, the distribution of the pseudo-osteodont histotype (i.e. simultaneous presence of osteodentine and orthodentine), seen in hexanchiforms is of particular interest as there seems to be a reduction of orthodentine (Jambura et al., 2020). The recovery of the osteodont pattern across two shark (Selachii) clades could potentially imply some degree of functionality on the crown histology.

### †*Archaeogracilidens* as a synechodontiform

The relationship between †*Archaeogracilidens* and synechodontiforms has been suggested repeatedly (e.g. Böttcher & Duffin, 2000; Duffin & Ward, 1993; Klug, 2010; Thies, 1983, 1993). Klug’s (2010) analysis was the first to propose this within a phylogenetic context. Re-analysing Klug’s (2010) data matrix under her study parameters revealed that this relationship is supported by a Jk of 56% (see Supplemental material). Klug’s (2010) study proposed two synapomorphies for the group, which are associated with the tooth root morphology (pseudopolyaulacorhize root vascularization and the presence of a labial root depression), but the presence of a labial depression is a character associated with a pseudopolyaulacorhize vascularization type (Cappetta, 1992, 2012). Consequently, the synechodontiform affiliation in Klug’s (2010) analysis heavily depends on how root vascularization is coded, specifically in relation to Casier’s (1947, 1961) and Cappetta’s (1987, 1992) classifications. The use of these root vascularization patterns resulted in scoring the root vascularization of squaliform teeth as anaulacorhize type. However, Casier (1947, 1961) typified the tooth vascularization in squaliform sharks as a subsequent stage of that seen in hybodontiforms. Cappetta (1987) identified the presence of variation within the tooth vascularization pattern for some squaliforms such as *Dalatias* Rafinesque, 1810, *Scymnodon* Barbosa du Bocage and de Brito Capello, 1864, and *Echinorhinus* de Blainville, 1816, suggesting that the pattern is less homogenous than previously assumed. Later, Cappetta (2012, p. 23) characterized the root vascularization of all squaliforms as holaulacorhize.

Cappetta (1987, 1992) introduced the pseudopolyaulacorhize vascularization pattern for palaeospinacid sharks *sensu* Cappetta (1992). Later, this pattern was related to additional taxa (Böttcher & Duffin, 2000; Klug et al., 2009; Klug, 2010; Thies, 1993). The inclusion of taxa like †*Welcommia cappettai* Klug and Kriwet, 2010 (see Klug & Kriwet, 2010, text-fig. 2A–E), †*Pseudonotidanus* Underwood and Ward, 2004 (see Cappetta, 2012, text-figs 91, 92; Underwood & Ward, 2004, pl. 9, 1–7) and †*Archaeogracilidens* (Fig. 4) complicates the identification of a generalized pattern of the key features (i.e. nutritive root grooves and labial root depression) as they present different degrees of development of these features.

Cappetta (2012) further complicated this by including hexanchiform sharks within the groups with a pseudopolyaulacorhize root vascularization pattern, as some Jurassic hexanchiforms such as †*Notidanoides* Maisey, 1986 and †*Crassodontidanus* Kriwet and Klug, 2011 exhibit root morphologies similar to those of †*Welcommia* Cappetta, 1990 and †*Pseudonotidanus*, and to a certain degree to †*Archaeogracilidens*. The changes in the interpretation of root vascularization patterns highlight the ambiguities in using these characters in phylogenetic studies. Additionally, the presence of sub-groups within generalized patterns, and the presence of multiple root vascularization types within taxonomic units, complicate the systematic application of these features (e.g. †*Protospinax* Woodward, 1918, *Parascyllium* (Duméril, 1853), and †*Synechodus* Woodward, 1888) (Underwood & Ward, 2004, pls 11–12; Herman et al., 1992, pl. 23; Andreev & Cuny, 2012). These issues emphasize the need to address taxonomic and systematic uncertainties when using of root vascularization data, to avoid the oversimplification and generalization of the character states.

Past studies (e.g. Klug, 2010) employed a strictly bin-ary coding scheme for root vascularization patterns, in which each pattern was included as independent binary characters (i.e. absent/present or yes/no) (see Klug, 2010, chs 165–169). This coding scheme was previously suggested to be a useful way to minimize subjective interpretations of evolutionary relationships in multistate characters by preventing an artificial increase in the number of evolutionary steps with no specified direction (Pleijel, 1995). However, this coding procedure has fallen into disuse, as separating a multistate character into multiple binary characters can lead to problems. Specifically, the illogical assignment of synapomorphies for nodes (i.e. one of the character states can be optimized as a synapomorphy supporting a node without it even being present in the terminals within the node) (Brazeau, 2011; Forey & Kitching, 2000; Hawkins, 2000; Hawkins et al., 1997; Strong & Lipscomb, 1999). This issue arises as one of the tokens (characters states) is transformed into a non-specific category, effectively serving as a catch-all for anything that simply implies different to x feature without reflecting the actual state present in the terminal (Brazeau, 2011). For example, Klug’s (2010) character 164 (anaulacorhize vascularization: yes [0]/no [1]), codes everything scored as [1] as lacking an anaulacorhize vascularization pattern, not considering the actual vascularization pattern present in the terminals coded as [1]. Similar instances of this coding method appear in Klug’s (2010) analysis (e.g. characters 145–147 and 155–161).

Binary coding additionally assumes independence across the series of characters, suggesting that the different vascularization types in neoselachians are not homologous. This would imply independent originations for each vascularization type. However, the vascularization patterns described in the literature (e.g. Casier, 1947; Cappetta, 1987, 2012), suggest instances of transition from one vascularization type to another. This observation implies some degree of homology is assumed by the classification schemes, which is not considered by earlier studies, such as Klug (2010).

The synechodontiform affiliation of †*Archaeogracilidens* is not recovered if these vascularization pattern characters, and other independent binary characters (145–147 and 155–161) are coded as unordered multistate characters (see Supplemental material: Klug matrix chs 172–174). The use of an unordered multistate coding method avoids illogical assumptions and reconstructions of ancestral states but also avoids the subjective interpretations regarding directions of character state changes. Additionally, the synechodontiform clade is lost under any other vascularization typification scheme that includes the pseudopolyaulacorhize stage (Cappetta, 1987, 2012) (see Supplemental material: Klug matrix chs 173–174). This advocates that the relationships recovered by Klug (2010) are the result of applying a nonadditive binary coding structure and the overgeneralization of characters. It is possible that the interpretation of these characters would have changed in the 2010 study with the addition of other fossil species to which the characters could be compared. This ultimately highlights the necessity of including numerous in- and outgroups in phylogenetic analyses. Additionally, the subsequent discovery of specimen SMNS 96844/7, which was not available at that time, has provided a lot of new information leading to the identification of new skeletal traits that were missing in previous studies (Böttcher & Duffin, 2000; Klug, 2010).

Thies (1983), Duffin and Ward (1993) and Böttcher and Duffin (2000) suggested a slightly different affiliation for †*Archaeogracilidens*. They placed it within Synechodontiformes, but in a sister-group relationship to Hexanchiformes, which was mainly based on dental features. Leidner and Thies (1999) proposed an analogous relationship based on similarities in dermal denticle morphology highlighting the presence of a horizontal ridge around the anterior end of the crown, which bears one or two knob- or spur-like protuberances, and a smooth neck surface below the ridge.

### †*Archaeogracilidens* as a hexanchiform shark

The present analyses (parsimony and Bayesian inference) places †*Archaeogracilidens* within Hexanchiformes, in a clade that includes the fossil hex-anchiform †*Notidanoides* Maisey, 1986. This clade is recovered in a sister group relationship to Hexanchidae (Fig. 15).

The placement of †*Archaeogracilidens* within a clade that includes †*Notidanoides* suggests that the family †Crassodontidanidae Kriwet and Klug, 2016 represents a junior synonym of †Orthacodidae Glickman, 1957. However, further revision is needed, including additional fossil hexanchiform taxa, to provide a more comprehensive view of the distribution of the vertebral and dental features within Hexanchiformes and to determine how these features should be interpreted (i.e. plesiomorphic or apomorphic). Consequently, to maintain taxonomic consistency, we retain both families, †Crassodontidanidae and †Orthacodidae, as separate entities.

Our study is not the first to suggest a possible hexanchiform affiliation for †*Archaeogracilidens* (=†*Sphenodus sensu* Quenstedt, 1865) (e.g. Blot, 1969; Guinot & Cappetta, 2011). However, it is the first one placing it among other Hexanchiform within a phylogenetic context (see also Cappetta, 2012).

The present placement necessitates a discussion on previous criticisms to a hexanchiform affiliation (e.g. Maisey et al., 2004). These critiques have been based on the general lack of derived features to support this placement. This concern remains relevant in the present study, as some relevant character states that could further clarify this relation could not be determined (?). This includes the mineralization of the antorbital process of the ectethmoid process (ch. 23) and the postorbital articulation (ch. 79). Additionally certain features like a deep groove for the insertion of the quadratomandibularis (ch. 80) and a large otic process of the palatoquadrate (ch. 81) are shared among other groups (e.g. Lamniformes or Ctenacanthiformes). Additional context on those features might provide further insight on the present results.

Addressing the first group of features (missing data) represents an inherent issue of any phylogenetic analysis and is particularly challenging for those analyses that include fossil taxa. Despite the extremely well-preserved condition of the specimens studied here, we were not able to observe certain characters such as the postorbital articulation or the ectethmoid process. In the case of the postorbital articulation, which was previously suggested for †*Archaeogracilidens* by Maisey (2008), the revision of specimen SMNS 96844/7 revealed the presence of a large otic process in the quadrate portion of the palatoquadrate, with a broad, concave surface positioned close to the postorbital process. This suggests the presence of a synovial cavity in this position, resembling the amphistylic articulation described for extant neoselachians by Maisey (2008). However, this interpretation cannot be confirmed, as potential deformation of the quadrate ridge during taphonomic processes and the lack of preserved soft tissue elements hinder unambiguous identification, resulting in a missing scoring [?] (see Supplemental material).

The second group of characters includes those that occur in more than one clade (e.g. a deep groove for the insertion of the adductor mandibular muscle and a large otic process of the palatoquadrate) (present among Hexanchidae). It is important to emphasize that synapomorphic grouping is a consequence of the parsimony optimization criterion, but not its primary goal. That main objective is to maximize the explanatory power (Farris, 1983; Goloboff, 2022), meaning that the goal is to explain most of the observed similarities among the analysed taxa in terms of common ancestry (Goloboff et al., 2021). Features can appear more than once while still providing valuable phylogenetic information. For this example, the groups that also share these features (Lamniformes and Ctenacanthiformes) are separated from Hexanchidae by several groups that lack them, suggesting the independent evolutionary acquisition of the disused features in each respective clade. Given the current data and the evidence from the material revision, the independent evolutionary acquisition of these features presents the most congruent and consistent interpretation (i.e. the one recovered in the most-parsimonious trees), and this is the primary consideration under the parsimony criterion.

Compared to previous studies proposing a galeomorph (e.g. Maisey et al., 2004) or synechodontiform (e.g. Böttcher & Duffin, 2000; Duffin & Ward, 1993; Klug, 2010) affiliation, the present placement of †*Archaeogracilidens* within Hexanchiformes reduces homoplasy (gains or losses of a feature within multiple groups). This includes diagnostic hexanchiform characters, like the amphistylic postorbital articulation pattern (see Maisey, 2004, 2008), and the presence of a single dorsal fin and vascularization patterns; but also those that are distributed among various groups (e.g. a deep groove for the insertion of the adductor mandibular muscle and a large otic process of the palatoquadrate). This ultimately maximizing the explanatory power of these features and the whole dataset among neoselachians.

## Conclusions

Böttcher and Duffin (2000) identified specific dental characters such as tooth size, distal inclination of the cusp, torsion of the cusp, width of the roots, and presence/absence of symmetrical cutting edges as diagnostic features for the species †’*Sphenodus*’ *macer* and †’*Sphenodus*’ *nitidus*, respectively. However, the revision of the holomorphic specimen considered to belong to †’*S*’. *macer* (SMNS 80142/44) (see Böttcher & Duffin, 2000) and specimen SNSB-BSPG AS VII 647 (holotype of †’*S*’. *nitidus*), indicates that these characters are not species specific, or cannot be directly observed in articulated material or compared. Consequently, we reject the definition of †’*S*.’ *macer* provided by Böttcher and Duffin (2000). While this conclusion might seem counterintuitive, given the apparent good preservation of the specimens included here (e.g. SMNS 96844/7), the damaged state or poor preservation of some structures in common among the examined specimens (e.g. length of the postorbital process) did not allow their comparison. Additionally, issues related to individual variation within a species or uncertainties regarding tooth positioning could not be satisfactorily addressed. Considering the lack of diagnostic characters between †’*S*.’ *macer* and †’*S*’. *nitidus*, along with the shared dental, cranial and postcranial features, these species are considered synonymous, with †’*S*’. *macer* taking priority. This agrees with previous assessments provided by de Beaumont (1960) and Schweizer (1964).

The taxonomic ambiguities surrounding the genus †*Sphenodus* Agassiz, 1843 result in the conclusion that the genus is a *nomen dubium*. Consequently, †*Archaeogracilidens* is introduced here as a replacement to the genus †*Sphenodus sensu* Quenstedt, 1865, based on well-preserved and diagnostically reliable specimens, thereby establishing a stable taxonomic scheme. †*Archaeogracilidens* currently is restricted to the type species †*Archaeogracilidens macer* (Quenstedt, 1851). Data from the assigned specimens, alongside an extensive literature review and phylogenetic analyses, indicate a close relationship between †*Archaeogracilidens* to other hexanchiforms.

The revision of previous hypotheses regarding the phylogenetic relationships of †*Archaeogracilidens* (Klug, 2010) suggests that †Synechodontiformes, at least within a phylogenetic context delimited in that study, is not a valid clade. However, several limitations remain in our analysis. Currently, only a single synechodontiform has been included, leaving the taxonomic composition and phylogenetic relationships of Synechodontiformes unresolved. Whether the monophyly of this group and its sister relationship to neoselachians is supported by other morphological data remains to be tested. A comprehensive revision of additional taxa traditionally included in Synechodontiformes is also needed.

Taxonomic revisions and investigations must always prioritize stability and universality (see also ICZN, 1999) to ensure accuracy and reliability. Future studies need to consider these implications and carefully evaluate the material and characters upon which the taxonomic groups are based. Likewise, when proposing new taxonomic units, special attention must be given to the proposed diagnostic features at the corresponding taxonomic level.

While the use of both cranial and postcranial skeletal features has proven to be very beneficial when evaluating palaeontological systematic hypotheses based on dental remains, this approach is often not possible for most fossil neoselachian groups, as their fossil record predominantly consists of isolated teeth. Integrating dental traits into broader phylogenetic analyses that incorporate cranial and postcranial traits thus is essential. However, as shown in the present study, significant dental variation within the jaws of an individual, as well as between individuals, can complicate the identification of taxonomic and systematic informative traits, especially when isolated teeth are considered. We therefore encourage researchers to thoroughly investigate dental morphological variation in both fossil holomorphic specimens and extant taxa. Only by doing so will it be possible to include dental traits of fossil taxa (known from isolated teeth) in robust phylogenetic analyses, ultimately clarifying their systematic position and avoiding para-taxonomic schemes for isolated tooth taxa.

## Supplementary Material

Supplemental material for this article can be accessed here: https://doi.org/10.1080/14772019.2025.2507014.

Documentary

Klug (2010) Log File

Log File

Phylogeny

Table 1

TNT Log File

## Figures and Tables

**Figure 14:**
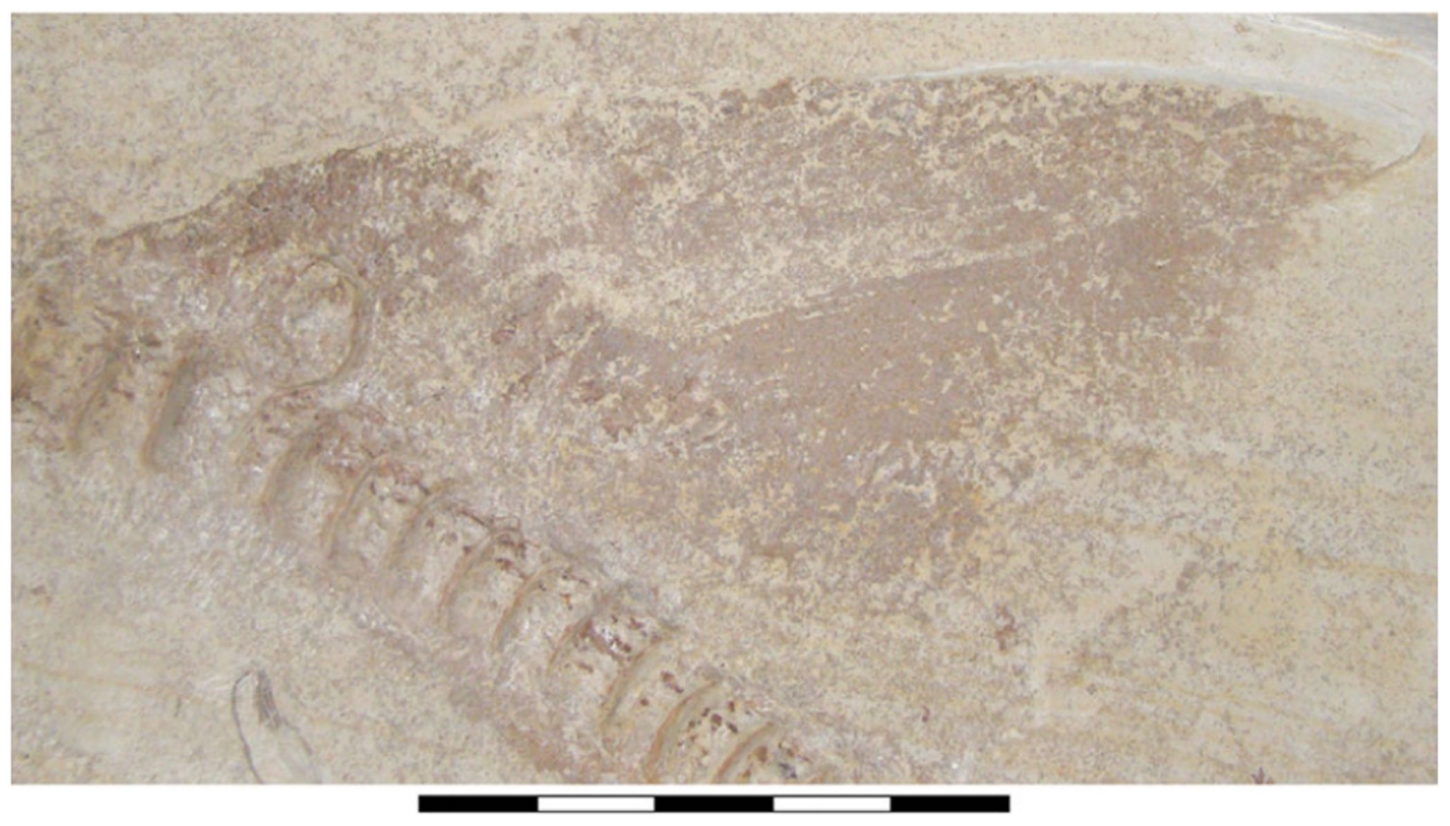
Dorsal fin of †*Archaeogracilidens macer*, specimen SMNS 96844/7 under normal light. Scale bar = 5 cm in A.

## Data Availability

The data that support the findings of this study are openly available in MorphoBank at https://doi.org/10.7934/P5764 (Villalobos-Segura et al., 2025).
