# Supplementary material for: Articulated specimens provide new insights into the iconic Mesozoic shark genus Sphenodus
Source: J Syst Palaeontol. 2025 Jun 20;23(1):2507014. doi: 10.1080/14772019.2025.2507014 (PMC7617882; doi:10.1080/14772019.2025.2507014)
Supplement: Supplemental Material [file TJSP_A_2507014_SM9186.zip › Supplemental material/Archaeogracilidens_Supmat_Revised.docx]

**﻿**

Table of Contents

[Aic scores for Model selection 2](#_Toc190099971)

[Tree comparison 2](#_Toc190099972)

[Character optimization 4](#_Toc190099973)

[Characters coding 8](#_Toc190099974)

[Additional miscellaneous modifications 32](#_Toc190099975)

[Institutional abbreviations 32](#_Toc190099976)

[Revised extant material 35](#_Toc190099977)

[Revision of Klug (2010) analysis 39](#_Toc190099978)

[References 40](#_Toc190099979)

# **Aic scores for Model selection**

| T | C | ratio | aic-gmk | aic-iwa | aic-MP |
| --- | --- | --- | --- | --- | --- |
| 93 | 211 | 0.441 | 9473.312 | 9694.962 | 9800.570 |

Best-to-Worst model

- **MK/IW/MP**

# **Tree comparison**


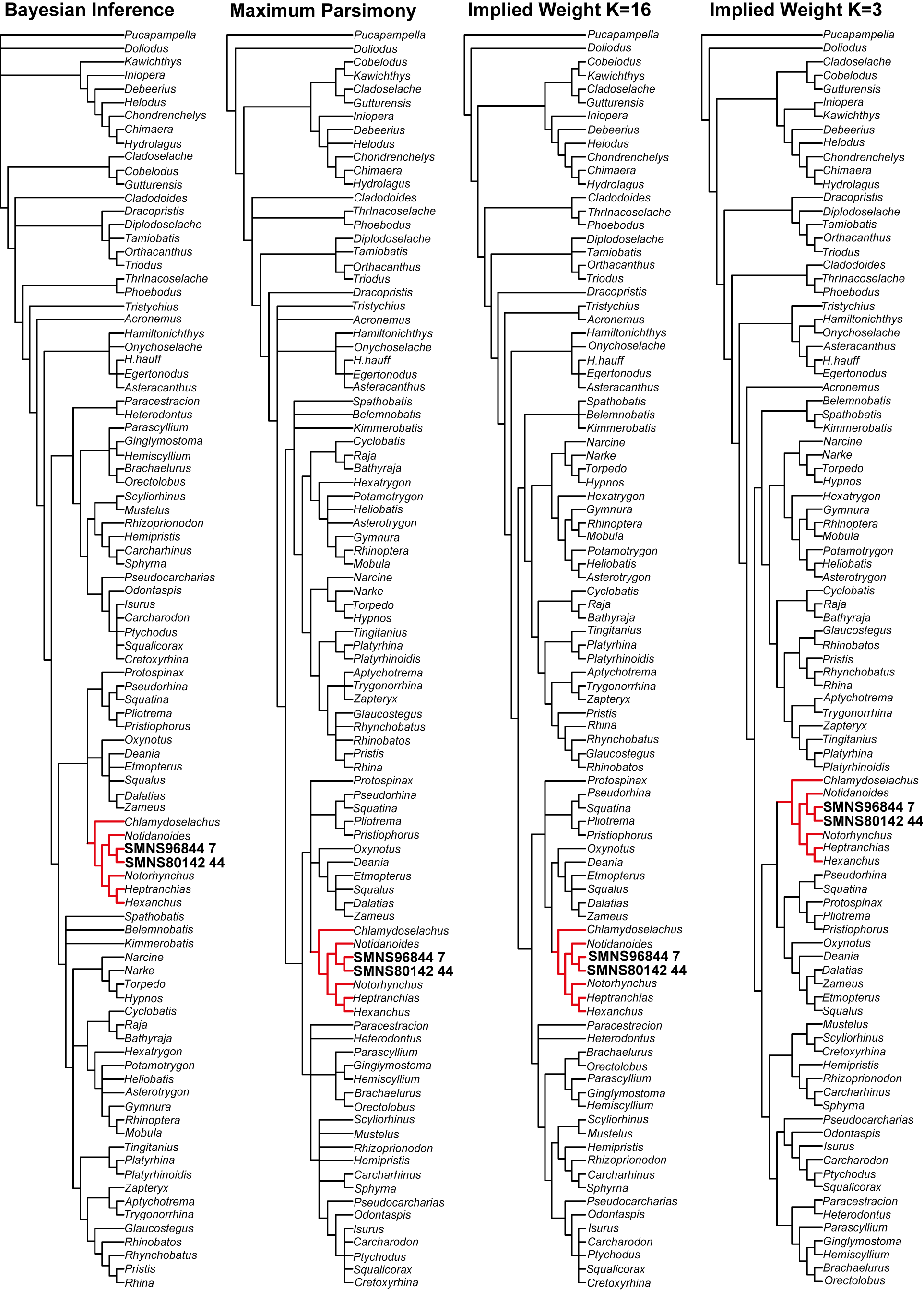


Figure S1. Majority rule Tree (Bayesian Inference) and Strict consensus trees (Parsimony) estimated in the present analyses. Marked in red is the Hexanchiformes clade.


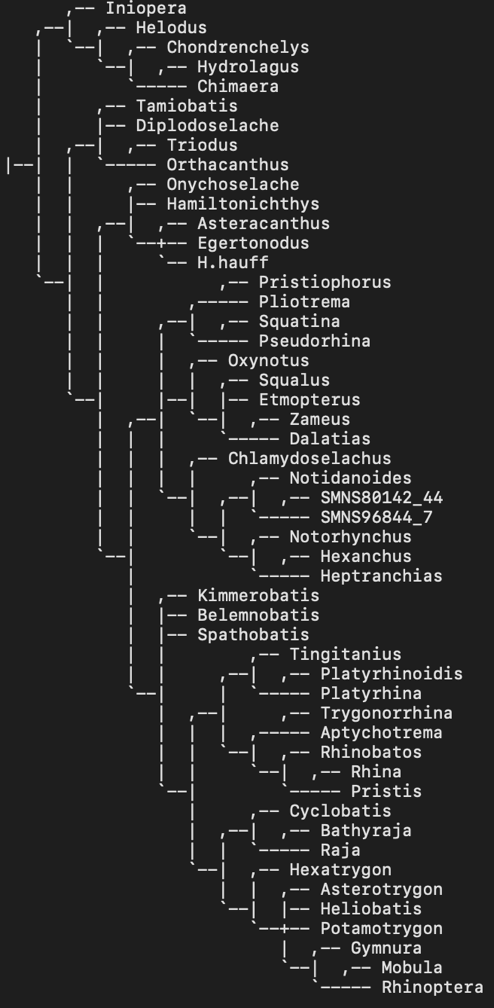


Figure S2. Agreement subtree estimated from the comparison between consensus trees of the Bayesian Inference and the Parsimony analysis under no implied weights.

| Tree | **Bay(MK)** | MP | IW K=16 | IW K=03 |
| --- | --- | --- | --- | --- |
| **Bay(MK)** | – | **5** | 13 | 16 |
| MP |  | – | 3 | 10 |
| IW K=16 |  |  | – | 15 |
| IW K=03 |  |  |  | – |

Table 1. SPR moves separating the estimated topologies in the different analyses. MK was the best fitting model and best score is marked in red.

| Tree | **Bay(MK)** | MP | IW K=16 | IW K=03 |
| --- | --- | --- | --- | --- |
| **Bay(MK)** | – | 0.9444 | 0.8556 | 0.8222 |
| MP |  | – | 0.9667 | 0.8889 |
| IW K=16 |  |  | – | 8333 |
| IW K=03 |  |  |  | – |

Table 2. Similarity score among the different topologies estimated in the different analyses. MK was the best fitting model and best score is marked in red.

# **Character optimization**


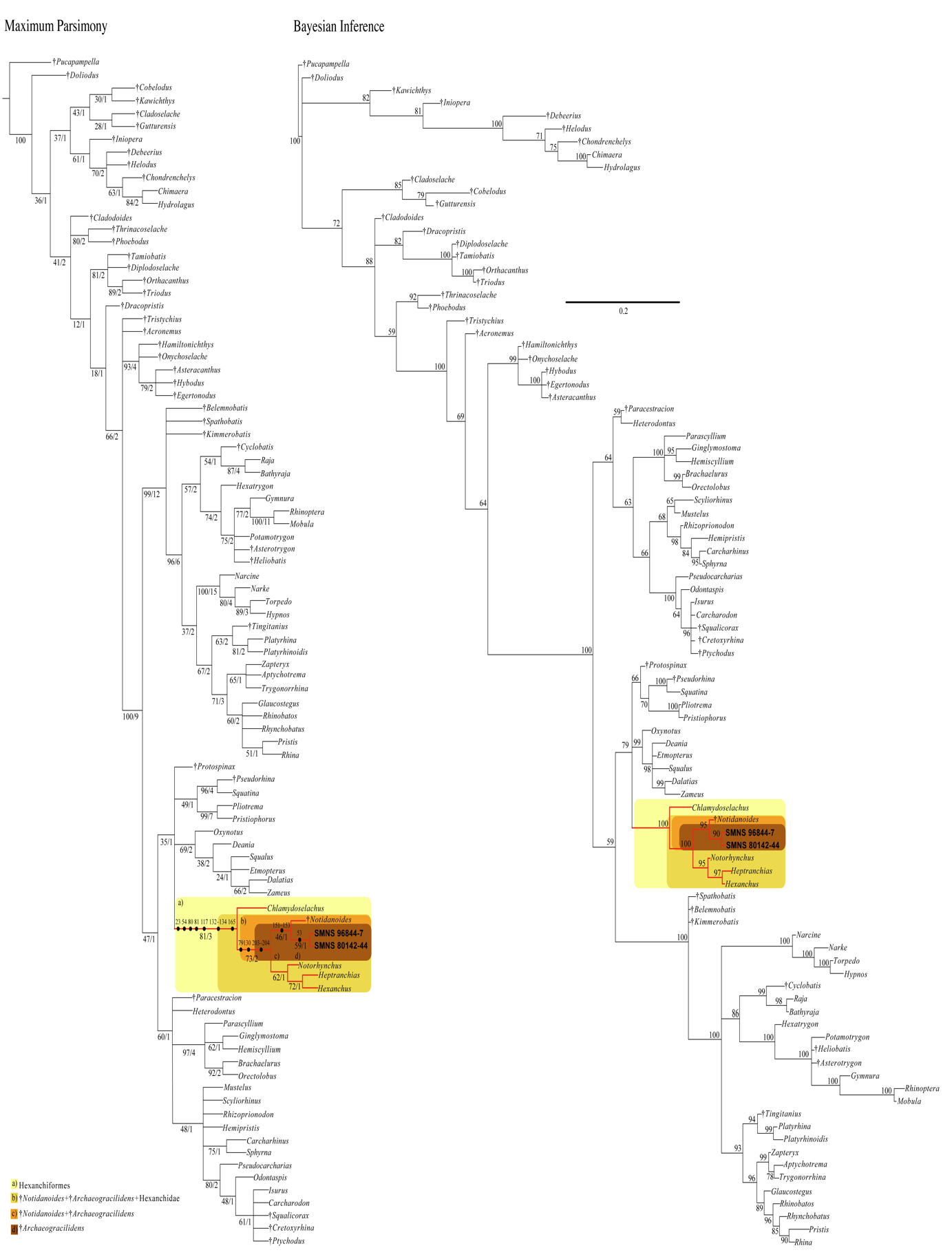


Figure S1. Strict consensus tree estimated in the present analysis. Numbers below the branches indicate the Jackknife and Bremer scores for the support of the clades (Jk/Br).

Characters supporting clades in tree. The characters marked with an asterisk are only supporting those nodes in most of the most parsimonious trees (MPt) but not in all.

Node 95: Char. 45: 0–>1

Node 96: Char. 20: 0–>1, Char. 118: 0–>1, Char. 129: 0–>1, Char. 132: 1,3,5–>0, Char. 137: 0–>1, Char. 141: 1–>0.

Node 97: Char. 47: 0–>1, Char. 53: 0–>1, Char. 56: 1–>0.

Node 98: Char. 72: 0–>1.

Node 99: Char. 117: 1–>0, Char. 132: 1–>2.

Node 100: Char. 149: 0–>1,2.

Node 101: Char. 35: 0–>1, Char. 51: 0–>1, Char. 52: 0–>1, Char. 129: 0–>2, Char. 210: 0–>1. * Char. 33: 0–>5, Char. 36: 0–>2, Char. 50: 0–>1.

Node 102: Char. 22: 1–>0, Char. 46: 0–>2, Char. 49: 1–>0, Char. 55: 1–>0, Char. 78: 0–>1, Char. 88: 1–>0. * Char. 12: 1–>0, Char. 75: 8–>4, Char. 86: 0–>1, Char. 115: 1–>0.

Node 103: Char. 40: 0 –>1, Char. 206: 0–>1.

Node 104: Char. 11: 2–>4, Char. 30: 0–>1. * Char. 41: 0–>1, Char. 58: 0–>1, Char. 116: 0–>1, Char. 118: 0–>1, Char. 120: 0–>1, Char. 165: 1–>0, Char. 168: 0–>2.

Node 105: Char. 80: 0–>1.

Node 106: Char. 40: 0–>1, Char. 73: 0–>1. * Char. 65: 0–>2, Char. 75: 8–>5.

Node 107: Char. 163: 0–>1, Char. 193: 0 –>1. * Char. 115: 1–>0, Char. 192: 2–>0.

Node 108: Char. 49: 1–>0, Char. 167: 2–>0.

Node 109: Char. 42: 0–>1, Char. 81: 1–>0, Char. 91: 0–>1, Char. 97: 1–>0, Char. 114: 0–>1, Char. 169: 1–>0.

Node 110: Char. 8: 0–>1, Char. 208: 0–>1.

Node 111: Char. 33: 0–>7, Char. 81: 0–>2, Char. 135: 1–>2, Char. 185: 0–>1 to 8. * Char. 163: 0–>1.

Node 112: Char. 206: 0–>1. * Char. 25: 0–>1, Char. 75: 5–>7, Char. 185: 1,3–>6,8.

Node 113: Char. 53: 1–>0.

Node 114: Char. 151: 5–>6.

Node 115: Char. 79: 0–>2, Char. 130: 4–>5, Char. 203: 0–>1,2.

Node 116: Char. 23: 0–>1, Char. 54: 1–>0, Char. 80: 0–>1, Char. 81: 0–>1, Char. 117: 0–>1, Char. 132: 7–>8, Char. 165: 1–>0. * Char. 45: 0–>1, Char. 85: 1–>0, Char. 110: 1–>0, Char. 160: 1–>0, Char. 192: 5–>0.

Node 117: Char. 31: 1–>2, Char. 75: 5–>6. * Char. 30: 0–>2, Char. 35: 3–>4, Char. 85: 0–>1, Char. 144: 0–>1, Char. 159: 0–>1.

Node 118: Char. 131: 1–>2, Char. 132: 5–>7.

Node 119: Char. 33: 0–>2, Char. 35: 0–>3, Char. 69: 0–>1, Char. 117: 1–>0, Char. 151: 0–> 5,7,8, Char. 154: 0–> 1, Char. 156: 0–>1, Char. 157: 0–>1, Char. 162: 0–>1. * Char. 1: 0–>1, Char. 4: 1–>0, Char. 45: 0–>1, Char. 59: 0–>1, Char. 65: 2–>1, Char. 79: 1–>0, Char. 139: 0–>1, Char. 146: 0–>1,2 Char. 167: 0–>1, Char. 192: 2,4–> 5, Char. 199: 0–>1,2.

Node 120: Char. 24: 0–>1, Char. 25: 0–>1, Char. 39: 0–>1, Char. 156: 1–>2.

Node 121: Char. 151: 5–>1.

Node 122: Char. 15: 0–>2, Char. 27: 0–>1, Char. 143: 0–>1.* Char. 30: 2–>0, Char. 53: 1–> 0.

Node 123: Char. 136: 0–>1, Char. 167: 1–>3, Char. 192: 5–>0.

Node 124: Char. 10: 0–>1, Char. 13: 1–>2, Char. 38: 0–>1, Char. 99: 1–>3, Char. 156: 1–>2, Char. 160: 1–>2, Char. 191: 0–>1.

Node 125: Char. 106: 0–>1. * Char. 7: 1–>0, Char. 53: 1–> 0, Char. 67: 1–>2.

Node 126: Char. 25: 0–>1, Char. 35: 4–>2.

Node 127: Char. 24: 0–>1. * Char. 7: 0–>1, Char. 202: 0–>2.

Node 128: Char. 205: 0–>1. * Char. 48: 0–>1, Char. 160: 1–>0, Char. 211: 1–>0.

Node 129: Char. 28: 1–>2. *Char. 3: 0–>3, Char. 17: 0–>1, Char. 37: 0–>1, Char. 107: 0–>1, Char. 160: 1–>0, Char. 202: 0–>2.

Node 130: Char. 108: 0–>1. * Char. 11: 0–>1, Char. 13: 1–>0, Char. 25: 0–>2, Char. 27: 0–>1, Char. 54: 1–>0, Char. 67: 1–>2, Char. 84: 0–>1, Char. 101: 1–>2, Char. 167: 1–>2, Char. 192: 5–>0.

Node 131: Char. 33: 2–>6, Char. 125: 0–>1. * Char. 132: 7–>0, Char. 151: 7–>6.

Node 132: Char. 53: 1–>0, Char. 85: 0–>1, Char. 176: 0–>1. * Char. 151: 6,7–>7.

Node 133: Char. 134: 1–>0. * Char. 3: 3–>2, Char. 9: 1–>0, Char. 11: 0–>1, Char. 13: 1–>0, Char. 114: 1–>0, Char. 151: 5–>7, Char. 162: 1–>2, Char. 167: 1–>2, Char. 192: 5–>0.

Node 134: Char. 130: 4 –>6. * Char. 118: 0–>1, Char. 131: 2–>3, Char. 166: 0–>1.

Node 135: Char. 80: 0–>1, Char. 81: 0–>1. * Char. 17: 0–>1, Char. 30: 0–>2, Char. 44 0,1–>1, Char. 92: 2–>5, Char. 151: 7–>6, Char. 162: 1,2–>2, Char. 202: 0,2–>1.

Node 136: Char. 53: 1–>0. * Char. 211: 1–>0.

Node 137: Char. 166: 0–>1. * Char. 118: 0–>1, Char. 168: 1–>0.

Node 138: Char. 7: 0–>1, Char. 34: 0–>1, Char. 137: 0–>1. * Char. 154: 1,2,3–>2.

Node 139: Char. 105: 0–>1, Char. 140: 0–>1, Char. 190: 0–>1.

Node 140: Char. 162: 1–>2, Char. 166: 0–>1. * Char. 142: 1–>0.

Node 141: Char. 25: 0–>1, Char. 124: 0–>1, Char. 130: 4–>6, Char. 131: 1–>3, Char. 132: 5–>9, Char. 158: 0–>1. * Char. 160: 1–>2, Char. 192: 5–>0.

Node 142: Char. 23: 0–>2, Char. 31: 1–>0, Char. 54: 1–>0, Char. 75: 5–>0, Char. 91: 1–>2, Char. 92: 2–>0, Char. 109: 0–>1, Char. 118: 0–>1, Char. 136: 0–>1, Char. 156: 1–>3, Char. 168: 1–>2. * Char. 98: 0–>1, Char. 99: 1–>4, Char. 114: 1–>0, Char. 142: 0–>1, Char. 154: 1–>1,3.

Node 143: Char. 23: 2–>3,5, Char. 28: 1–>0, Char. 33: 2–>5, Char. 89: 0–>1, Char. 96: 0–>1, Char. 111: 0–>1, Char. 112: 1–>0, Char. 117: 0–>1, Char. 121: 0–>1, Char. 127: 1,2,3–>0, Char. 130: 6–>7, Char. 140: 0–>1, Char. 158: 1–>2, Char. 184: 0–>1, Char. 189: 1–>0.

Node 144: Char. 5: 0–>3, Char. 85: 0–>1. * Char. 154: 2,3–>1,2.

Node 145: Char. 23: 3,5–>4, Char. 25: 1,2–>0, Char. 85: 1–>0.

Node 146: Char. 5: 3–>1, Char. 15: 0–>1, Char. 67: 1–>2, Char. 99: 4,5> 0, Char. 175: 0–>1, Char. 176: 0–>1.

Node 147: Char. 13: 1–>3, Char. 16: 0–>1.

Node 148: Char. 35: 3–>5, Char. 43: 0–>1, Char. 105: 0–>1, Char. 124: 1–>2, Char. 181: 0–> 2, Char. 201: 0–>1.

Node 149: Char. 114: 0–>1, Char. 158: 1–>2, Char. 164: 0–>1.

Node 150: Char. 23: 2–> 6,7, Char. 177: 1–>0, Char. 181: 2–>1.

Node 151: Char. 5: 3–>0, Char. 7: 0–>1, Char. 18: 0–>1, Char. 45: 1–>0, Char. 137: 0–>1. * Char. 154: 1,2–>4.

Node 152: Char. 25: 1–>0. * Char. 142: 1–>0, Char. 154: 4–>2, Char. 177: 0–>1.

Node 153: Char. 13: 1–>0, Char. 33: 2–>3, Char. 35: 3–>5, Char. 124: 1–>3, Char. 155: 0–>1, Char. 198: 0 –>1. * Char. 25: 1–>2.

Node 154: Char. 65: 1–>0, Char. 137: 0–>1, Char. 138: 0–>1, Char. 168: 2–>3. * Char. 7: 1–>0, Char. 119: 1,2–>0, Char. 189: 1–>0.

Node 155: Char. 15: 0–>1, Char. 165: 1–>2, * Char. 7: 0–>1, Char. 95: 0–>1, Char. 105: 0–>2, Char. 157: 1–>2.

Node 156: 67: 1–>2, Char. 99: 4–>2, Char. 164: 0–>1.

Node 157: Char. 2: 1–>2, Char. 18: 0–>1, Char. 33: 3–>6, Char. 74: 0–>1, Char. 83: 0–>1, Char. 99: 2,4,5–>0, Char. 122: 0–>1, Char. 158: 1–>3, Char. 165: 2–>0, Char. 172: 0–>1. *Char. 28: 2–>1, Char. 157: 2–>1.

# **Characters coding**

The description of characters can be found in the citations in bold. Marked with an asterisk are the citations where the character was first modified (that modification being applied in the present analysis). Goloboff et al., (2021) protocol was used for the analysis. This protocol usages the affixes Sup (superior) and Sub (subordinate) to denote the logical relation between characters. These characters will be the ones analysed with step matrices by TNT.

**Neurocranium (Skeleton)**

1. **Sup Rostral cartilages:** [0] arise from the medial area of the trabecula only, [1] medial area of the trabecula + lamina orbitonasalis. **Villalobos-Segura *et al*. (2022, Char. 3)**. Based on de Beer (1937), Miyake *et al*. (1992) and Lane (2010)**.**
2. **Sub Rostral cartilage:** [0] well-developed rostral plate with various degrees of contribution from the lamina orbitonasalis, [1] reaches the tip of the snout (carried by the growth of the pectoral fin, [2] reaches the tip of the snout (growth of lamina orbitonasalis to support the cephalic fins). **Villalobos-Segura *et al*. (2022, Char. 4).**
3. **Sub Rostral cartilage “rostrum”:** [0] trough-like rostrum, [1] tripodal rostrum, [2] greatly reduced to medial bar. Landemaine *et al*. (2018, Char. 1), Klug (2010, Char. 1), Goto (2001, Char. 21), de Carvalho (1996, Char. 1), **Shirai (1996, Char. 1)**.
4. **Internasal plate separating the two palatoquadrates:** [0] absent, [1] present. **Pradel et al. (2011, Char. 4**).
5. **Sup Rostral processes:** [0] absent, [1] present. Villalobos-Segura *et al*. (2022, Char. 7), Villalobos-Segura *et al*. (2019, Char. 32), **Aschliman *et al*. (2012,** **Char. 29)**.
6. **Sub Rostral processes (proximal articulation):** [0] articulated with nasal capsules, [1] continuous with chondrocranium, [2] articulated with ventral aspect of rostral cartilage. **Villalobos-Segura *et al*. (2022, Char. 8)**.
7. **Rostral appendix:** [0] absent, [1] present. **Aschliman *et al*. (2012, Char. 25)**.
8. **Elongate, tooth-bearing, pre-nasal ethmo-rostral region:** [0] absent, [1] present. **Frey *et al*. (2020, Char. 106)**.
9. **Ethmoidal region of neurocranium (down-curved):** [0] absent, [1] present. †*Ptychodus***[?]**. Landemaine *et al*. (2018, Char. 3), Klug (2010, Char. 3), Goto (2001, Char. 9a), **de Carvalho (1996, Char. 4)**, **Shirai (1996, Char. 2)**.
10. **Rostral passage of superficial ophthalmic nerve:** [0] covered, [1] open. **Villalobos-Segura *et al*. (2022, Char. 11)**.
11. **Trigeminofacial complex:** [0] exits neurocranium through prootic foramen, [1] separate foramen for superficial ophthalmic nerve, [2] emerged from the braincase via a single foramen and then branched to form separate dorsal (superficial ophthalmic) and ventral (bucco-maxillary) complexes. The superficial ophthalmic complex passes through a short canal in the dorsal part of the postorbital arcade and merges below the roof of the orbit. The prootic foramen and the foramen for the hyomandibular branch of facialis (hmVII) are in anteromedial position, [3] separate foramen for superficial ophthalmic nerve with prootic foramen and foramen for hmVII going through the subocular shelf, [4] The superficial ophthalmic complex passes through a short canal in the dorsal part of the postorbital arcade and merges below the roof of the orbit. The prootic foramen and the foramen for hmVII are in posteromedial position, [5] three separate foramina, foramen for hmVII in anteromedial position, [6] single foramina for the whole complex, [7] three separate foramina, foramen for hmVII in posteromedial position. Vullo et al. (2024, Char, 11)*, Landemaine *et al*. (2018, Char. 6), Klug (2010, Char. 6), de Carvalho (1996, Char. 7), Shirai (1996, Char. 9), **Shirai (1992, Char. 5)**. See also Allis (1914).
12. **Precerebral fontanelle:** [0] absent, [1] present. Villalobos-Segura *et al*. (2022, Char. 142)., Landemaine *et al*. (2018, Char. 23), Klug (2010, Char. 23(17)), de Carvalho & Maisey (1996, Char. 4), **Shirai (1992, Char. 13)**.
13. **Sup precerebral fossa:** [0] absent, [1] present. Landemaine *et al*. (2018, Char. 2 & 43), Klug (2010, Char. 2(2) & 43(31)), de Carvalho (1996, Char. 3), de Carvalho & Maisey (1996, Char. 5)*, Shirai (1996, Char. 3), **Shirai (1992, Char. 14)**.
14. **Sub precerebral fossa:** [0] circular or ovoid concavity, [1] extending anteriorly and roofed to form a tube. Landemaine *et al*. (2018, Char. 2 & 43), Klug (2010, Char. 2(2) & 43(31)), **Shirai (1996, Char. 3)**.
15. **Nasal capsules:** [0] laterally expanded, [1] ventro-laterally expanded, [2] anteriorly expanded, [3] trumpet-like. Villalobos-Segura *et al*. (2022, Char. 34)*, Villalobos-Segura *et al*. (2019, Char. 34)*, Landemaine *et al*. (2018, Char. 44), Claeson *et al*. (2013, Char. 10), Aschliman *et al*. (2012, Char. 31), McEachran & Aschliman (2004, Char. 27), Klug (2010, Char. 44(32)), McEachran *et al*. (1996, Char. 23), Shirai (1996, Char. 4), **Nishida (1990, Char. 37)**.
16. **Nasal capsule anterior margin (“horn like process”):** [0] absent, [1] present. Villalobos-Segura *et al*. (2022, Char. 35), Villalobos-Segura *et al*. (2019, Char. 83), Claeson *et al*. (2013, Char. 9), **Brito & Seret (1996, Char. 5)**.
17. **Nasal capsules separated from orbital:** [0] absent, [1] present. **Vullo *et al.* (2024, Char. 17)**.
18. **Anterior preorbital foramen:** [0] located dorsally, [1] located anteriorly. Villalobos-Segura *et al*. (2022, Char. 12), Villalobos-Segura *et al*. (2019, Char. 37), Aschliman *et al*. (2012, Char. 35), McEachran *et al*. (1996, Char. 27), **Nishida (1990, Char. 85).**
19. **Preorbital process (nasal capsule):** [0] absent, [1] present. Modified from Villalobos-Segura *et al*. (2022, Char. 13)., Aschliman *et al*. (2012, Char. 33), **de Carvalho & Maisey (1996, Char. 16)**, McEachran *et al*. (1996, Char. 25), Shirai (1992, Char. 33 & 34 & 35), Nishida (1990, Char. 17).


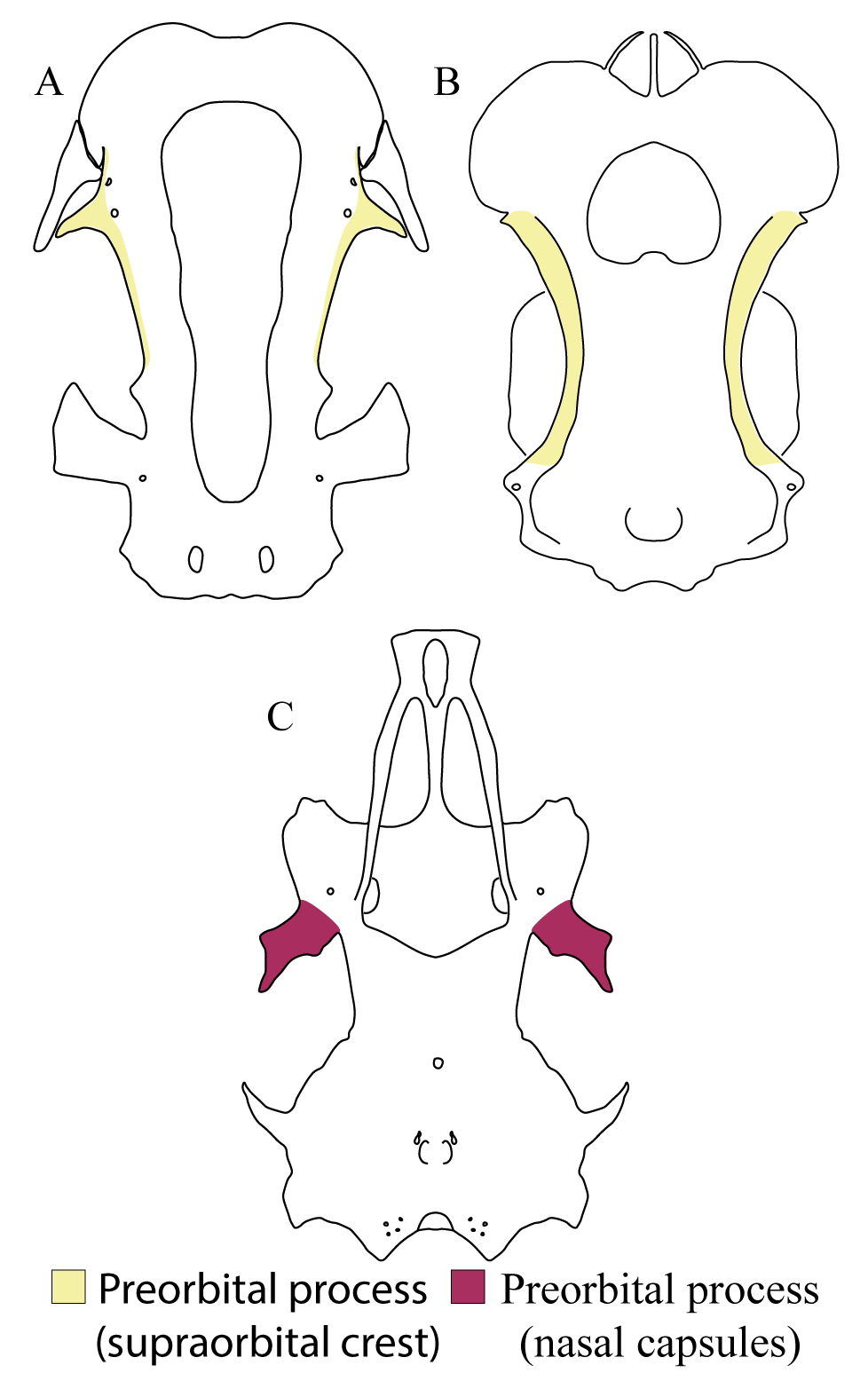


**Figure S2.** Line draws of the character states recognized for character 19. Dorsal view of the neurocranium of: A. Potamotrygon yepezi Castex & Castello, 1970 (redrawn from Nishida, 1990 text-fig. 13A). B. Scyliorhinus haeckelii (de Miranda-Ribeiro, 1907) (redrawn from Soares et al., 2016 text-fig. 17A, UERJ 1690). C. Carcharhinus falciformis (Bibron in Müller & Henle, 1841) (redrawn from de Oliviera Lana et al., 2021 text-fig 1A, AC.UERJ 1456). **State [0]**: A-B. **State [1]**. C.

1. **Sclerotic ring:** [0] absent, [1] present**. Frey *et al*. (2020, Char. 22),** Coates *et al*. (2018), Burrow *et al*. (2016), Zhu *et al*. (2016, Char. 275), Qiao *et al*. (2016, Char. 277), Giles *et al*. (2015, Char. 52).
2. **Interorbital space:** [0] broad, [1] narrow. **Frey *et al*. (2020, Char. 110)**. Based on Brazeau (2009), Davis *et al*. (2012), Zhu *et al*. (2013), Coates *et al*. (2017).
3. **Optic pedicel:** [0] absent, [1] present. **Frey *et al*. (2020, Char. 111)**. Based on Dupret *et al*. (2014), Zhu *et al*. (2009, 2013), Coates *et al*. (2017).


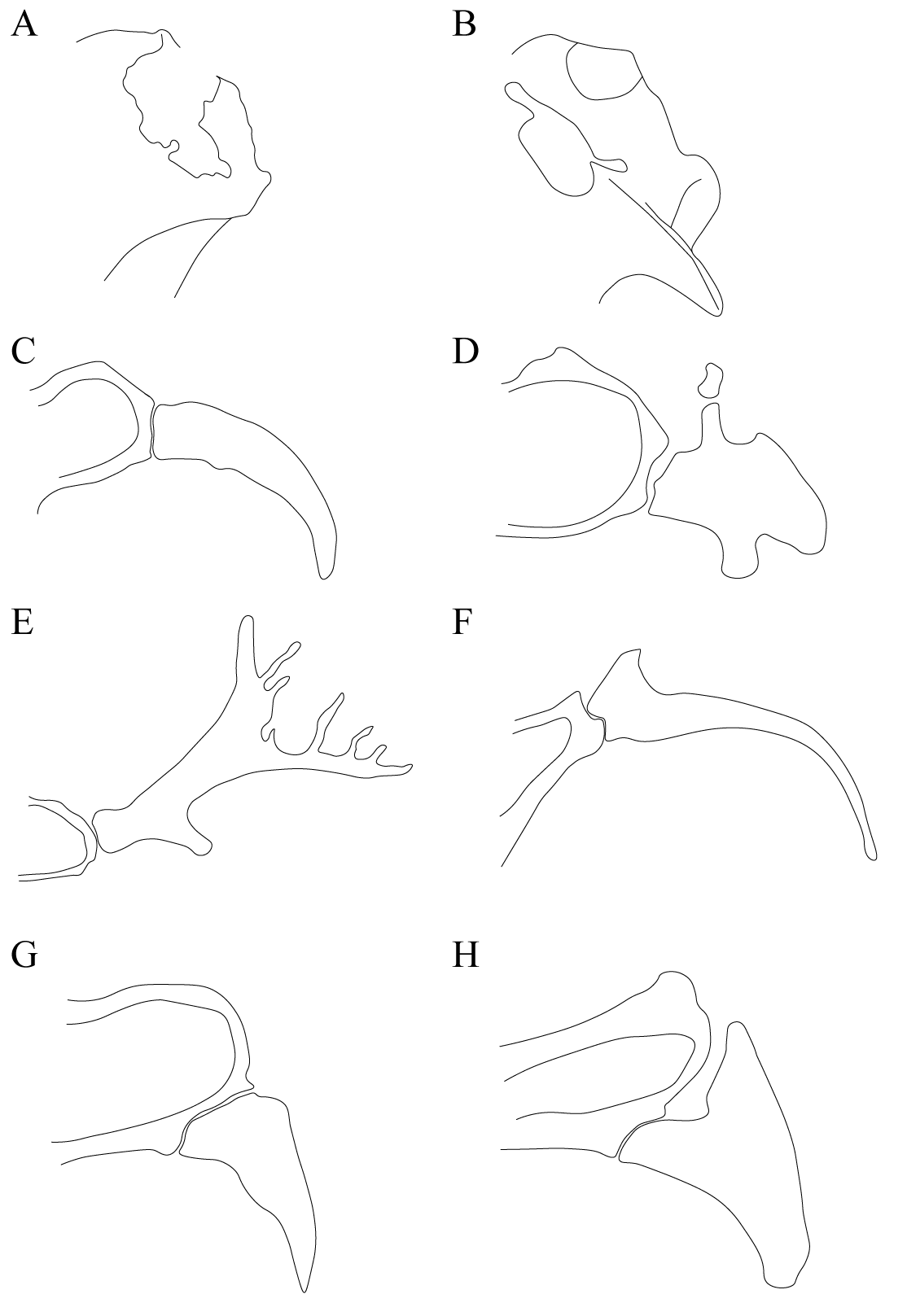


Figure S3. Line drawing illustrating the character states scored for character 23. Ventral view of the nasal capsule of: A. Dalatias licha (Bonnaterre, 1788) (redrawn and modified from Shirai, 1992, Plate 8C, HUMZ 74603). B. Chlamydoselachus anguineus Garman, 1884 (redrawn and modified from Allis, 1923, Plate. 9, Fig. 8). Ventral view of the nasal capsule and antorbital cartilage of: C. Aptychotrema vincentiana (Haacke, 1885) (CSIRO 101, <https://sharksrays.org/>, accessed on 20 Oct. 2023); D. Platyrhinoidis triseriata (Jordan & Gilbert, 1880) (MNHN 3211); E. Narcine brasiliensis (von Olfers, 1831) (AMNH 77069, <https://sharksrays.org/>, accessed on 20 Oct. 2023); F. Torpedo fuscomaculata Peters, 1855 (USNM, <https://sharksrays.org/>, accessed on 20 Oct. 2023); G. Rhinobatos productus (Ayres, 1854) (CNPE-IBUNAM 17829); H. Rhynchobatus springeri Compagno & Last, 2010 (HO 180, <https://sharksrays.org/>, accessed on 20 Oct. 2023). **State [0]**: A. **State [1]**. B. **State [2]**: C. **State [3]**. D. **State [4]**: E. **State [5]**. F. **State [6]**: G. **State [7]**. H.

1. **Antorbital process:** [0] membranous, [1] mineralization of the antorbital process in the presence of the ectethmoid process, [2] mineralized in absence of an ectethmoid process (antorbital cartilages, triangular shaped with regular outline, lateral articulation), [3] antorbital cartilages, variously shaped and with an irregular outline, lateral articulation, [4] antorbital cartilages, variously shaped and with an irregular outline, anterolateral articulation, [5] antorbital cartilages, triangular shaped with regular outline, anterolateral articulation, [6] antorbital cartilages, triangular shaped with regular outline, postero-lateral articulation, [7] antorbital cartilages, triangular shaped with regular outline, postero-lateral articulation, anterior process. Villalobos-Segura *et al*. (2022, Char. 23; Char. 24; Char. 26; Char. 110), Villalobos-Segura *et al*. (2019, Char. 8; Char. 9), Landemaine *et al*. (2018, Char. 7), Claeson *et al*. (2013, Char. 5), Aschliman *et al*. (2012, Char. 8), Klug (2010, Char. 7), de Carvalho (2004, Char. 2), Shirai (1996, Char. 10), de Carvalho (1996, Char. 8)*****, Brito & Seret (1996, Char. 2), **Nishida (1990, Char. 3)**.
2. **Subnasal fenestra:** [0] absent, [1] present. Klug (2010, Char. 5), de Carvalho (1996, Char. 6), de Carvalho & Maisey (1996, Char. 6), Shirai (1996, Char. 7), **Shirai (1992, Char. 15)**.
3. **Sup_Epiphyseal foramen:** [0] absent, [1] present. Jambura *et al*. (2023, Char. 26)*, **Goto (2001, Char. 10)**.
4. **Sub_Epiphyseal foramen:** [0] isolated, [1] fused with prefrontal fontanelle. Jambura *et al*. (2023, Char. 27)*, **Goto (2001, Char. 10)**.
5. **Supraotic shelf:** [0] narrow, [1] broad. **Frey *et al*. (2020, Char. 161)**.
6. **Sup_Supraorbital crest:** [0] absent, [1] present. Vullo et al. (2024, Char. 28-29)*, Villalobos-Segura *et al*. (2022, Char. 20), Achliman *et al*. (2012, Char. 34), McEachran *et al*. (1996, Char. 26), **Nishida (1990, Char. 32)**.
7. **Sub_Preorbital process (supraorbital crest):** [0] absent, [1] present. Vullo et al. (2024, Char. 28-29)*, Villalobos-Segura *et al*. (2022, Char. 13), Aschliman *et al*. (2012, Char. 33), de Carvalho & Maisey (1996, Char. 16), McEachran *et al*. (1996, Char. 25), Shirai (1992, Char. 33 & 34 & 35), **Nishida (1990, Char. 17)** (see Figure S3).
8. **Occipital crest:** [0] absent, [1] present separated from the brain cavity by the otic occipital fissure, [2] present but with otic occipital fissure absent. Vullo *et al.* (2024, Char. 30)
9. **Sup_Basicranial processes:** [0] absent, [1] present. Modified form Villalobos-Segura *et al*. (2022, Chars. 40–41), de Carvalho & Maisey (1996, Char. 3), Shirai (1992, Char. 44).
10. **Sub_Basicranial processes (position):** [0] trabecular, [1] polar. Villalobos-Segura *et al*. (2022, Chars. 40–41), de Carvalho & Maisey (1996, Char. 3), Shirai (1992, Char. 44).
11. **Postorbital process:** [0] forming part of the arcade, **[1]** arcade present C bout notch separated postorbital process from supraotic shelf, [2] not forming part of the arcade, [3] not forming part of the arcade C bout notch separates postorbital process from supraotic shelf, [4] narrow, [5] reduced (not developed), [6] shelflike laterally expanded, [7] shelflike antero ventrally expanded. Modified from Vullo *et al.* (2024, Char. 33), Villalobos-Segura *et al*. (2022, Char. 27)**,** Claeson *et al*. (2013, Char. 12), McEachran & Aschliman (2004, Char. 32), Brito & Seret (1996, Char. 7), **Nishida (1990, Char. 35)**.

**Remarks:** We applied a multistate scoring for this character to accommodate all the variation observed in the taxa included in the present analysis, implying homology among the different configurations of the postorbital process among taxa included in the present analysis. Characters 35 and 58 of Vullo et al. (2024) is included as states within this multistate character. Whilst this could be considered a conglomerative character, the close relation between the elements involved suggest a correlation in the development of the structures.

**
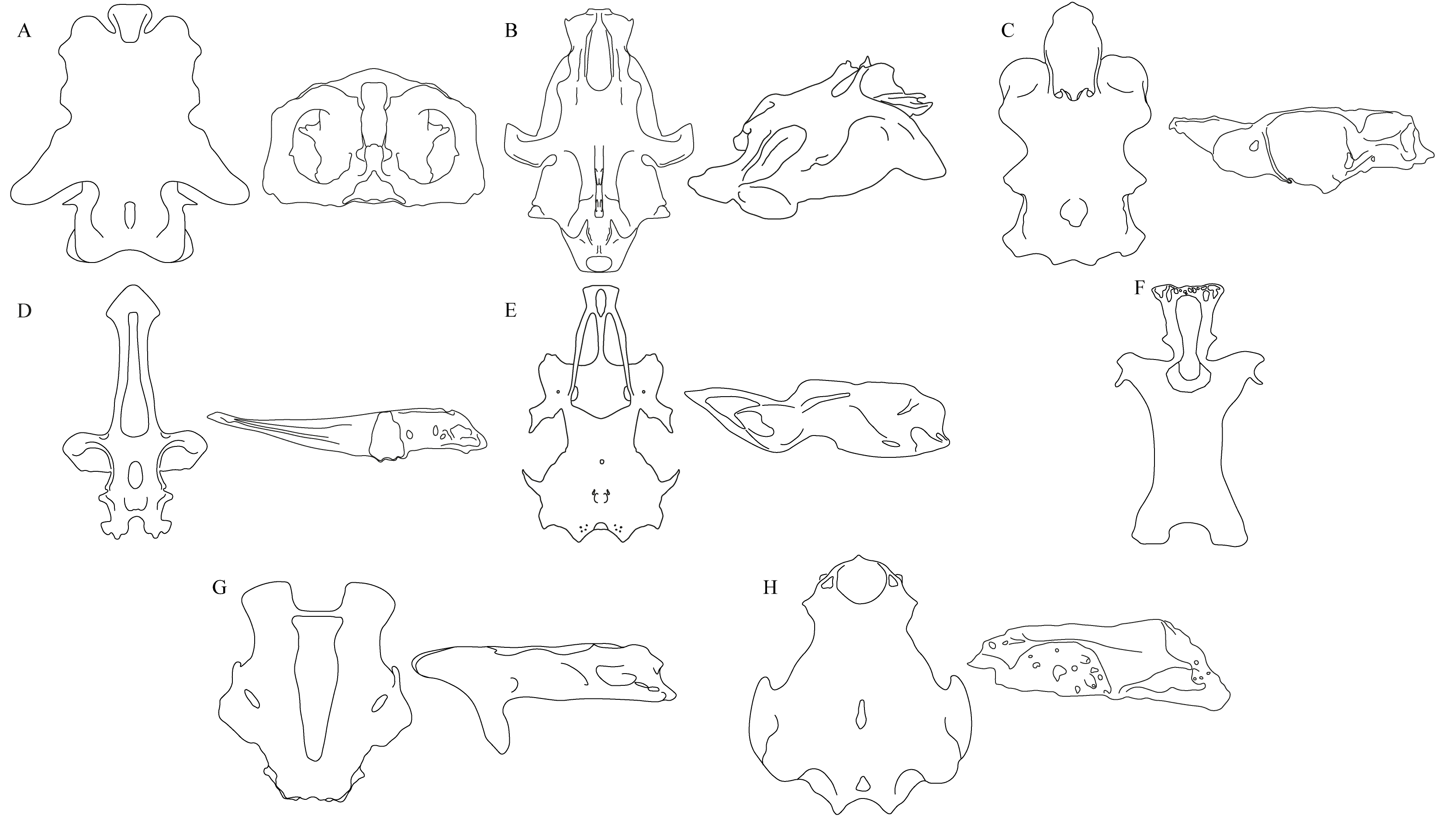
**

Figure S4. Line drawing showing the character states scored for character 33. A. Dorsal view of a neurocranium of †Cobelodus aculeatus (Cope, 1894) (redrawn from Maisey, 2008, text-figs. 39 and 48A, FMNH PF 7347), frontal view of a possible neurocranium of †Cobelodus specimen (redraw from Maisey, 2008, text-fig. 10, FMNH PF 13242). B. Dorsal and lateral reconstructions of a neurocranium of †Tristychius arcuatus Agassiz, 1837 (redrawn from Coates & Tietjen, 2018, text-fig. 3A,E, based on their observations on NMS 1972.27.461A & B, 1972.27.481D, 1974.23.6, 1974.23.19, 1974.23.30A & B, 1974.51.2A & B, 2015.30.1A & B). C. Dorsal view of a neurocranium of Squalus acanthias Linné, 1758 (redrawn from Thomas et al. 2016, text-figure 1), lateral view of a neurocranium of Squalus acanthias (redrawn and modified from Shirai, 1992, Plate 8C). D. Dorsal view of a neurocranium of Rhinobatos glaucostigma Jordan & Gilbert, 1883 (CNPE-IBUNAM 17810), lateral view of a neurocranium of Rhinobatos schlegelii Müller & Henle, 1841 (redrawn and modified from Holmgren, 1941, text-fig. 46). E. Dorsal and lateral views of a neurocranium of Carcharhinus falciformis (Bibron in Müller & Henle, 1841) (redrawn from de Oliviera Lana et al., 2021 text-fig 1A, C, AC.UERJ 1456). F. Dorsal view of a neurocranium of Torpedo ocellata (AMNH 4128). G. Dorsal and lateral view of a neurocranium of Gymnura japonica (Temminck & Schlegel, 1850) (redrawn from Nishida, 1990, text-fig 15A, 19E, HUMZ 4830). H. Dorsal reconstruction of a neurocranium of †Egertonodus (†Hybodus) basanus redrawn and modified from Maisey, 1983, text-figure 15C, based on Maisey’s observations on NHMUK 40718, 60110, 6356, 11870), lateral view of a neurocranium of †Egertonodus Maisey, 1987 (redrawn from Lane 2010, text-fig. 33, NHMUK P60110). State [0]: A. State [1]: B. State [2]: C. State [3] D. State [4]: E. State [5]: F. State [6]: G. State [7]: H.

1. **Triangular process:** [0] absent, [1] present. Modified from Villalobos-Segura *et al*. (2022, Char. 29), Aschliman *et al*. (2012, Char. 37), **McEachran *et al*. (1996, Char. 29)**.

**Remarks:** The presence of a triangular process was defined in function of the presence of a postorbital process and thus, in previous analysis (Vullo *et al.* 2024), it was considered dependent to the presence of the latter. In the present analysis in order to accommodate the variation associated to the postorbital process (Char. 33) a multistate scoring was employed and consequently this logical relation between the postorbital process and triangular process was separated and both characters were codified as independent characters.

1. **Lateral commissure:** [0] **f**orms part of the postorbital arcade, [1] absent, due to the lack of spiracular cartilages and hyoid arch with not supporting connection with the mandibular arch, [2] lateral commissure is not fully chondrified and not enclosing the foramen prooticum, **[3]** reduced, [4] continuous and strong. enclosing the foramen prooticum, [5] narrow and situated behind the foramen prooticum in close relation to the hyomandibular fossa. **Vullo *et al.* (2024, Char. 36)**.
2. **Jugular canal diameter:** [0] small, [1] large, [2] canal absent**. Frey (2020, Char. 126)**. Based on Pradel *et al.* (2011),
3. **Hyomandibular fossa:** [0] posterior part of the otic region, [1] anteriorly situated in the otic region. Goto (2001, Char. 7a), de Carvalho (1996, Char. 13), Shirai (1996, Char. 17), **Shirai (1992, Char. 1).**
4. **Hyomandibular fossa:** [0] not composed of two horizontally divided cavities, [1] fossa with cavities as such. Landemaine *et al*. (2018, Char. 13), Klug (2010, Char. 13), Shirai (1996, Char. 18), **de Carvalho (1996, Char. 14)**, de Carvalho & Maisey (1996, Char. 29), Shirai (1992, Char. 54)*.


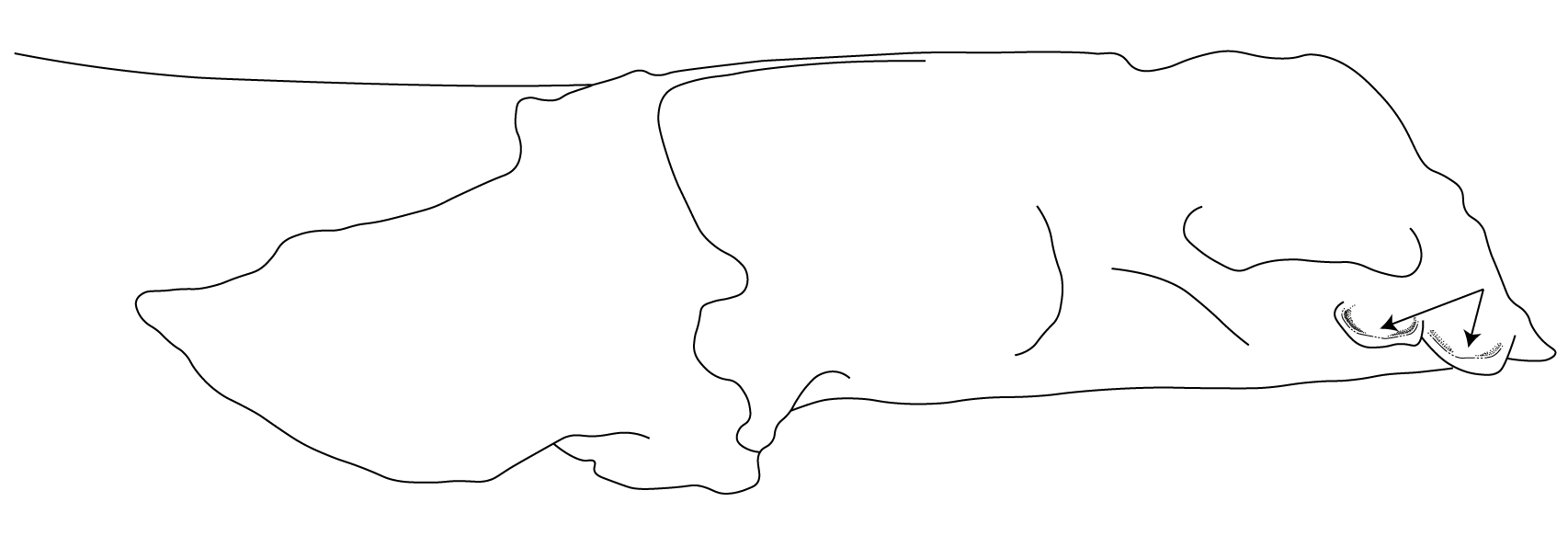


Figure S5. Line drawing illustrating character state [1] of character 38. Lateral view of the neurocranium of Rhynchobatus djiddensis (Forsskål 1775) (redrawn from Shirai 1992, plate 15D, HUMZ 6135).

1. **Concavity ventral to hyomandibular fossa:** [0] absent, [1] present. Landemaine *et al*. (2018, Char. 14), Klug (2010, Char. 14), **de Carvalho (1996, Char. 15)**, de Carvalho & Maisey (1996, Char. 26), Shirai (1996, Char. 19), Shirai (1992, Char. 51).
2. **Dorsal otic ridge:** [0] absent, [1] present. **Frey *et al*. (2020, Char. 162)**. Based on Coates and Gess (2007, Char. 4)
3. **Lateral otic process:** [0] absent, [1] present. **Frey *et al*. (2020, Char. 138)**. Based on observations by Schaeffer (1981), Coates & Sequeira (1998), Brazeau (2009), Davis *et al*. (2012) and Zhu *et al*. (2013) Bronson et al. (2004, Char. 124).
4. **Postotic process:** [0] absent, **[1]** present. Vullo et al. (2024, Char. 43)*, **Frey *et al*. (2020, Char. 141)**, Bronson et al. (2004, Char. 127).
5. **Jugal arch:** [0] absent, [1] present. Villalobos-Segura *et al*. (2022, Char. 22), Aschliman *et al*. (2012, Char. 39), **McEachran *et al*. (1996, Char. 31)**.
6. **Stapedial arch**: [0] absent, [1] present. Vullo et al. (2024, Char. 45)*, **Shimada (2005, Char. 22)**.
7. **Entrance of internal carotids:** [0] through a common opening at the central midline of the basicranium, [1] through separate openings flanking the hypophyseal opening or recess. Vullo et al. (2024, Char. 45)*, **Frey *et al*. (2020, Char 116)**.
8. **Canal for dorsal aorta and/or lateral dorsal aortae:** [0] runs inside the neurocranium, [1] runs external, no groove and enters brain, [2] runs externally and does not enter the brain directly, [3] runs externally, through a groove, and enters brain. Vullo *et al.* (2024, Char. 46)*, **Coates *et al*. (2017, Char. 117)**.
9. **Dorsal aorta divides into lateral dorsal aorta:** [0] posterior to occipital level, [1] anterior to level of the occiput. **Frey *et al*. (2020, Char 176)**. Based on Pradel *et al*. (2011), Giles *et al*. (2015) and Coates *et al*. (2017).
10. **Basal angle of the neurocranium:** [0] absent, [1] present. Landemaine *et al*. (2018, Char. 35), Klug (2010, Char. 35 (24)), Goto (2001, Char. 4a), de Carvalho & Maisey (1996, Char. 22), Shirai (1992, Char. 47).
11. **Metotic (otic-occipital) fissure:** [0] absent, [1] present. **Frey *et al*. (2020, Char. 169)**. Based on Coates and Gess (2007, Char. 7).
12. **Space for forebrain and at least proximal portion of olfactory tracts narrow and elongate extending between orbits:** [0] absent, [1] present. **Frey *et al*. (2020, Char. 169)**.
13. **Orbitonasal lamina expanded:** [0] absent, [1] present. **Frey *et al*. (2020, Char. 105)** also see Patterson (1965).
14. **Ophthalmic foramen in anterodorsal extremity of orbit communicates with enclosed cranial space:** [0] absent, [1] present. **Frey *et al*. (2020, Char. 113)**.
15. **Orbit vs otic capsule:** [0] orbit smaller than otic capsule, [1] obit larger than otic capsule. **Frey *et al*. (2020, Char. 122)**.
16. **Orbital artery foramen:** [0] absent, [1] present. Vullo *et al*. (2024, Char. 56).
17. **Canal, likely for nerve (V) mandibular ramus, passes through the postorbital process from proximal dorsal entry to distal and ventral exit:** [0] absent, [1] present**. Frey *et al*. (2020, Char. 127)**.
18. **Trigemino-facial recess:** [0] absent, [1] present. Vullo *et al.* (2024, Char. 57)*, **Frey *et al*. (2020, Char. 130)**. Based on Goodrich (1930), Gardiner (1984), Pradel (2010), Pradel *et al*. (2011), Davis *et al*. (2012)**.**
19. **Periotic process:** [0] absent, [1] present. **Frey *et al*. (2020, Char. 134)**. Based on Maisey (2007), Coates *et al*. (2017).
20. **Sub-otic occipital fossa:** [0] absent, [1] present. **Frey *et al*. (2020, Char. 140)**.
21. **Otic capsule extends posterolaterally relative to occipital arch:** [0] absent, [1] present. **Frey *et al*. (2020, Char. 144)**. Based on Maisey (1985).
22. **Endocranial roof anterior to otic capsules domelike, smoothly convex dorsally and anteriorly:** [0] absent, [1] present. **Frey *et al*. (2020, Char. 145)**.
23. **Roof of skeletal cavity for cerebellum and mesencephalon significantly higher than dorsal-most level of semicircular canals.** [0] absent, [1] present**. Frey *et al*. (2020, Char. 146)**.
24. **Labyrinth cavity separated from the main neurocranial cavity:** [0] by a cartilaginous capsular wall, [1] medial capsular wall absent. **Frey *et al*. (2020, Char. 148)**. Based on Pradel *et al*. (2011), Davis *et al*. (2012), Zhu *et al*. (2013).
25. **Sinus superior:** [0] absent or indistinguishable from union of anterior and posterior canals, [1] present, elongate and nearly vertical. Vullo *et al*. (2024, Char. 66)*, **Frey *et al*. (2020, Char. 155)**. Based on Davis *et al*. (2012), Zhu *et al*. (2013).
26. **External opening for endolymphatic ducts:** [0] posterior to crus commune, [1] anterior to crus commune. **Frey *et al*. (2020, Char. 160)**. Based on Coates *et al*. (2017).
27. **Sup Endolymphatic fossa:** [0] absent, [1] present. Vullo *et al*. (2024, Char. 68)*, **Frey *et al*. (2020, Char. 164).**
28. **Sub Endolymphatic fossa elongate (slot-shaped), dividing dorsal otic ridge along midline:** [0] absent, [1] present. Vullo *et al*. (2024, Char. 69)*, **Frey *et al*. (2020, Char. 165).**
29. **Perilymphatic fenestra:** [0] un-calcified, [1] separate opening form the endolypmphatic fenestra, [2] common opening with the endolypmphatic fenestra, [3] posterior to the anterior pair of small rounded endolymphatic foramina. **Frey *et al*. (2020, Char. 166).**
30. **Hypotic lamina:** [0] absent, **[1]** present. Schaeffer (1981); Maisey (1984, 2001); Brazeau (2009); Pradel *et al*. (2011, 2013); Davis *et al*. (2012); Zhu *et al*. (2013), Coates *et al*. (2017) and Maisey *et al*. (2019) **Frey *et al*. (2020, Char. 171)**.
31. **Glossopharyngeal nerve path:** [0] directed posteriorly and exits through metotic fissure or foramen in posteroventral wall of otic capsule, [1] exits laterally through a canal contained ventrally (floored) by the hypotic lamina, [2] exits through a foramen anterior to the posterior ampulla. Vullo *et al*. (2024, Char. 72)*, **Coates *et al*. (2017, Char. 115).**
32. **Glossopharyngeal and vagus nerves share common exit from neurocranium:** [0] absent, [1] present. **Frey *et al*. (2020, Char. 173).**
33. **Basicranial morphology:** [0] platybasic, [1] tropibasic. **Frey *et al*. (2020, Char. 77).**
34. **Ventral portion of occipital arch wedged between rear of otic capsules:** [0] absent, **[1]** present. **Frey *et al*. (2020, Char. 174).**
35. **Dorsal portion of occipital arch wedged between otic capsules:** [0] absent, [1] present. **Frey *et al*. (2020, Char. 178).**
36. **Antimeres of upper and lower jaws:** [0] separated, [1] fused. Villalobos-Segura *et al*. (2022, Char. 78), Frey *et al*. (2020, Char. 99)**,** Aschliman *et al*. (2012, Char. 40), **McEachran *et al*. (1996, Char. 32)**.
37. **Sup Craniopalatine articulation:** [0] absent, [1] present. Landemaine *et al*. (2018, Char. 47), Klug (2010, Char. 47(35), **Shirai (1996, Char. 11),** Vullo et al. (2024, Chars. 78-80)*.
38. **Sub Craniopalatine articulation (Fusion):** [0] fused, [1] articulated. **Frey *et al*. (2020, Char. 92),** Vullo *et al*. (2024, Chars. 78-80)***.**
39. **Sub Craniopalatine articulation (articulation):** [0] ethmoidal, [1] orbitostylic, [2] palatine and ethmoidal, [3] grooved in ethmoidal region. Vullo *et al*. (2024, Chars. 78-80)*, Landemaine *et al*. (2018, Char. 47), Klug (2010, Char. 47(35)), Goto (2001, Char. 2a), de Carvalho & Maisey (1996, Char. 20), **Shirai (1996, Char. 11), Shirai (1992, Char. 43).**
40. **Jaw articulation located on rearmost extremity of mandible:** [0] absent, [1] present. Davis *et al*. (2012), Zhu *et al*. (2013), **Frey *et al*. (2020, Char. 95)**.
41. **Postorbital articulation:** [0] absent, [1] lateral, [2] dorsal. **Villalobos-Segura *et al*. (2022, Char. 17)**, Klug (2010, Char. 11), de Carvalho (2004, Char. 12), **de Carvalho & Maisey (1996, Char. 15)***, Shirai (1996, Char. 14), **Shirai (1992, Char. 32),** Vullo *et al*. (2024, Char. 82)**.**
42. **Oblique ridge or groove along medial face of palatoquadrate:** [0] absent, [1] present. **Frey *et al*. (2020, Char. 88).**
43. **Large otic process of the palatoquadrate:** [0] absent, [1] present, [2] present forming a quadrate flange**.** **Villalobos-Segura *et al*. (2022, Char. 19),** Vullo *et al*. (2024, Char. 84)*.
44. **Meckel's cartilage:** [0] not expanded medially, [1] expanded medially. Villalobos-Segura *et al*. (2022, Char. 79), Aschliman *et al*. (2012, Char. 41), **McEachran *et al*. (1996, Char. 33).**
45. **Winglike process on Meckel's cartilage:** [0] absent, [1] present. Villalobos-Segura *et al*. (2022, Char. 80)**,** Aschliman *et al*. (2012, Char. 42)**,** McEachran *et al*. (1996, Char. 34), **Nishida (1990, Char. 86).**
46. **Sustentaculum:** [0] absent, [1] present. **Jambura *et al*. (2023, Char. 57),** Vullo *et al*. (2024, Char. 87).
47. **Quadratomandibularis process:** [0] absent, [1] present. **Vullo *et al*. (2024, Char. 88)**.
48. **Dental trough adjacent to oral rim on Meckel's cartilage and palatoquadrate:** [0] absent, [1] present. **Frey *et al*. (2020, Char. 97)**.
49. **Dental trough divided, scalloped tooth-bearing margin on Meckel's cartilage and palatoquadrate:** [0] absent, [1] present. **Frey *et al*. (2020, Char. 97)**.
50. **Gill skeleton position:** [0] posterior to the occipital region, [1] partly beneath otico-occipital regions. Villalobos-Segura *et al*. (2022, Char. 140), **Coates *et al*. (2017, Char. 29**).
51. **Spiracularis:** [0] undivided, [1] divided. **Villalobos-Segura *et al*. (2022, Char. 65),** Aschliman *et al*. (2012, Char. 85), McEachran *et al*. (1996, Char. 61).
52. **Coracohyomandibularis:** [0] single origin, [1] separate origins. Villalobos-Segura *et al*. (2022, Char. 68)**, Aschliman *et al*. (2012, Char. 88),** McEachran *et al*. (1996, Char. 64).
53. **Hyoid arch:** [0]: reduced, having no insertion of the dorsal constrictor muscle, hyoid arch well behind the jaw cartilages, [1] massive, holding the mandibular arch from behind, [2] composed of reduced ventral parts (ceratohyal missing) and developed hyomandibula, the latter suspending the lower jaw directly, [3] similar to State 2, but the articulation between the hyomandibula and mandible is interrupted by a ligament. [4] postorbital articulation non-suspensory, hyomadibula running dorsally to the palatoquadrate. [5] postorbital articulations present, hyomandibula suspensory (reduced) not protruding jaws. Vullo *et al*. (2024, Char. 94).
54. **Sup_Ceratohyal:** [0] absent, [1] present. **Aschliman *et al*. (2012, Char. 49).**
55. **Sub_Ceratohyal spatulate or bladed anteriorly:** [0] absent, **[1]** present. **Frey *et al*. (2020, Char. 55).**
56. **Sub Ceratohyal** [0] resembling branchial arches, [1] with broad posteroventral flange or shelf projecting laterally into recess behind Meckel's cartilage, [2] expanded posteriorly with a flange projecting laterally and posteriorly fitting the posterior edge of the Meckel’s cartilage. Vullo *et al*. (2024, Char. 97).
57. **Small cartilages associated with hyomandibular-Meckelian ligament:** [0] absent, [1] present. Villalobos-Segura *et al*. (2022, Char. 75), Aschliman *et al*. (2012, Char. 47), **McEachran *et al*. (1996, Char. 38)**.
58. **Mid part of hyomandibula:** [0] narrow, [1] expanded. Villalobos-Segura *et al*. (2022, Char. 73)**, Aschliman *et al*. (2012, Char. 44).**

**
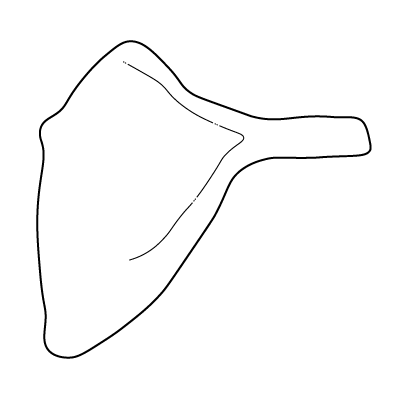
**

Figure S6. Line drawing illustrating the character state [1] of character 96. Dorsal view of the hyomandibula of Torpedo Duméril, 1806 (NHMUK 72261).

1. **Hypohyals:** [0] absent, [1] present. **Frey *et al*. (2020, Char. 56)**.
2. **Pseudohyal:** [0] absent, [1] present. Villalobos-Segura *et al*. (2022, Char. 47)**, Aschliman *et al*. (2012, Char. 3)**.
3. **Sup_Basihyal:** [0] absent, [1] present. Modified from Villalobos-Segura *et al*. (2022, Char. 44)*, Villalobos-Segura *et al*. (2019, Char. 48), **Aschliman *et al*. (2012, Char. 48)**, Jambura *et al*. (2023, Char. 70).
4. **Sub_Basihyal:** [0] as long as broad, [1] elongated, [2] basihyal very thin (connected to ceratohyal), [3] basihyal very thin (no ceratohyal), [4] reduced. (Modified from Landemaine *et al*. (2018, Char. 22 & 25), Klug (2010, Char. 22 & 25), de Carvalho (2004, Char. 65), **de Carvalho & Maisey (1996, Char. 8)**, Jambura *et al*. (2023, Char. 71)***.**
5. **Sup_Segmented basibranchial:** [0] absent, [1] present. Landemaine *et al*. (2018, Char. 131), Klug (2010, Char. 132(72)), de Carvalho (2004, Char. 22), de Carvalho & Maisey (1996, Char. 43), **Shirai (1996, Char. 32), Shirai (1992, Char. 90**), Vullo *et al*. (2024, Char. 105)*.
6. **Sub_Segmented basibranchial (Series):** [0] anterior, [1] posterior, [2] both. **Vullo *et al*. (2024, Char. 106)**.
7. **Second to last Hypobranchials direction:** [0] not directed towards midline, **[1]** midline directed. **Villalobos-Segura *et al*. (2022, Char. 38)**.
8. **Hypobranchials-basibranchial (relation):** [0] articulated, [1] fused. **Villalobos-Segura *et al*. (2022, Char. 42)**.
9. **First hypobranchial-basihyal:** [0] separated, [1] fused [2] segmented. **Villalobos-Segura *et al*. (2022, Char. 45)**.
10. **Hypobranchial bar:** [0] absent, [1] present. Landemaine *et al*. (2018, Char. 132), Klug (2010, Char. 133(73)), de Carvalho (2004, Char. 23), **Shirai (1996, Char. 33**).
11. **Pharyngobranchial blade:** [0] absent, [1] present. Landemaine *et al*. (2018, Char. 134), Klug (2010, Char. 135(75)), Goto (2001, Char. 1a), de Carvalho (2004, Char. 67), **Shirai (1996, Char. 35)**.
12. **Bifurcate ceratobranchials:** [0] absent, [1] present. Goto (2001, Char. 29).
13. **Last ceratobranchial:** [0] free from scapulocoracoid, [1] articulates with scapulocoracoid. (Villalobos-Segura *et al*. (2022, Char. 43), Landemaine *et al*. (2018, Char. 174), Aschliman *et al*. (2012, Char. 4), Shirai (1996, Char. 29), Shirai (1992, Char. 86), **Nishida (1990, Char. 5)**.
14. **Posterior most elements of dorsal gill arches:** [0] not completely fused, [1] completely fused (gill “pickaxe”). Landemaine *et al*. (2018, Char. 133), Klug (2010, Char. 134(74)), de Carvalho (2004, Char. 24), **Shirai (1996, Char. 34**).
15. **Branchial electric organs:** [0] absent, [1] present. Villalobos-Segura *et al*. (2022, Char. 21), **Aschliman *et al*. (2012, Char. 86**).
16. **Sup_Coracohyoideus:** [0] present, [1] absent. Villalobos-Segura *et al*. (2022, Char. 70), Aschliman *et al*. (2012, Char. 89), **McEachran *et al*. (1996, Char. 65)**.
17. **Sub_Coracohyoides (if present):** [0] parallel to body axis, [1] short, [2] diagonal, [3] fused. Villalobos-Segura *et al*. (2022, Char. 71)**,** Aschliman *et al*. (2012, Char. 89), McEachran *et al*. (1996, Char. 65).
18. **Labial cartilages:** [0] absent, [1] present. Modified from Landemaine *et al*. (2018, Char. 138), **Klug (2010, Char. 139)**.

**Girdles and paired fins**

1. **Fin base articulation on scapulocoracoid:** [0] stenobasal, deeper than wide; [1] eurybasal wider than deep. Lu *et al*. (2016), **Frey *et al*. (2020, Char. 197).**
2. **Biserial pectoral fin endoskeleton:** [0] absent, [1] present. Lu *et al*. (2016), **Frey *et al*. (2020, Char. 201).**
3. **Ventral antimeres of scapulocoracoid:** [0] fused, [1] not coalescent. Villalobos-Segura *et al*. 2022 (Char. 105), de Carvalho & Maisey (1996, Char. 1), **Shirai (1992, Char. 3**).
4. **Paired fin rays:** [0] aplesodic, [1] plesodic. **Aschliman *et al*. (2012, char. 68)**, Villalobos–Segura *et al*. (2019, char. 66).
5. **Radial calcification:** [0] crustal, [1] catenated (two chains), [2] catenated (four chains). Vullo *et al.* (2024, Char. 124)*, **Marramà *et al*. (2018 char. 100)**.
6. **Scapular process-scapula:** [0] fused, [1] articulated. **Villalobos-Segura *et al*. (2022, Char. 98).**
7. **Scapular process:** [0] short and dorsally directed, [1] long, U-curved, posteriorly directed, [2] short postero-dorsally directed. Villalobos-Segura *et al*. (2022, Char. 99)*, **Aschliman *et al*. (2012, Char. 86)**.
8. **Scapular process:** [0] without fossa, [1] with fossa. **Villalobos-Segura *et al*. (2022, Char. 100)**.
9. **Scapular posterior process**: [0] absent, [1] posterodorsal triangular process of scapula, [2] both dorsal and ventral triangular processes present, [3] posteroventral triangular process of scapula.

**Remarks:** This character is added based on the observations made by Silva *et al*. (2018), and Coates and Gess (2007, Char. 1) and includes Char. 179 of Vullo *et al*. (2024).

1. **Sup Suprascapulae:** [0] absent, [1] present. **Villalobos-Segura *et al*. (2022, Char. 93)**, Goto (2001, Char. 30), Aschliman *et al*. (2012, Char. 6).
2. **Suprascapular cartilages:** [0] absent, [1] present. **Vullo *et al*. (2024, Char. 129)**.
3. **Sub Suprascapulae (articulation):** [0] curved**,** [1] crenated, [2] ball socket**. Villalobos-Segura *et al*. (2022, Char. 96),** Vullo *et al*. (2024, Char. 130)***.**
4. **Sub Suprascapulae Sup Interacts with axials skeleton:** [0] absent (free from axial skeleton), [1] present. **Villalobos-Segura *et al*. (2022, Char. 94)**.
5. **Sub Interacts with axials skeleton:** [0] articulates with vertebral column, [1] fused medially to synarcual, [2] fused medially and laterally to synarcual. **Villalobos-Segura *et al*. (2022, Char. 95**).
6. **Pectoral basal elements:** [0] large basal element, [1] multiple free radials articulating directly and a metapterygium, [2] two main cartilages (propterygium and metapterygium), [3] tribasal pectoral fin, [4] all three basal elements fused, [5] propterygium and mesopterygium fused. **Villalobos-Segura *et al*. (2022, Char. 141),** Vullo *et al*. (2024, Char. 133)***.**
7. **Metapterygium:** [0] pectinated, [1] rectangular, [2] rectangular narrowing distally, articulating with a sequence of multiple axial cartilages, [3] rectangular articulated to a massive axial cartilage, [4] elongated with a broad distal end to which the pectoral radials articulate, [5] proximally segmented, with a broad distal end to which the pectoral radials articulate, [6] elongated with multiple radials articulating across its entire outer surface, [7] multiple segments. **Frey *et al*. (2020, Char. 199)**.
8. **Mesopterygium:** [0] reduced, [1] expanded of similar size as the propterygium, [2] enlarged broader and larger than the propterygium, [3] reduced propterygium and metapterygium larger. **Vullo *et al*. (2024, Char. 135)**.
9. **Sup Propterygium:** [0] absent, [1] present. **Vullo *et al*. (2024, Char. 136-138)**.
10. **Sub Propterygium:** [0] rectangular and being the point of articulation for the rest of basal elements, [1] narrow and reduced, [2] enlarged, similar size as the mesopterygium, **[3]** broad and reduced, [4] anteriorly expanded. **Vullo *et al*. (2024, Char. 136-138)**.
11. **Sub Propterygium:** [0] articulating with radials, [1] not contacting radials. Vullo *et al*. (2024, Char. 136-138)*, Landemaine *et al*. (2018, Char. 109), Klug (2010, 110(52)), Goto (2001, Char. 5a), de Carvalho (2004, Char. 40), Shirai (1996, Char. 63), **Shirai (1992, Char. 141**).
12. **Pectoral articulation:** [0] facets, [1] condyles, [2] facets and condyles. Vullo *et al*. (2024, Char. 139), taken from Silva and de Carvalho (2015).
13. **Independent metacondyle:** [0] absent, [1] present. **Vullo *et al*. (2024, Char. 140).**
14. **Pectoral fin radials:** [0] all articulate to pterygia, [1] some articulate directly with scapulocoracoid. **Villalobos-Segura *et al*. (2022, Char. 114**).
15. **Pectoral fin with interradial connections (“cross-braces”):** [0] absent, [1] present. Villalobos-Segura *et al*. (2022, Char. 111), Shirai (1996, Char. 67).
16. **Pelvic girdle:** [0] separated, [1] fused. Villalobos-Segura *et al*. (2022, Char. 124).
17. **Lateral prepelvic process:** [0] absent, [1] present. Villalobos-Segura *et al*. (2022, Char. 117)***, McEachran & Dunn (1998, Char. 36**).
18. **Pelvic basals:** [0] multiple radials articulated directly to the pelvic girdle**,** the latest fused into the baspterygium**,** [1] two large basal cartilages**,** [2] single large element. **Villalobos-Segura *et al*. (2022, Char. 60**).
19. **Postpelvic processes:** [0] absent, [1] present. **Villalobos-Segura *et al*. (2022, Char. 118), Claeson *et al*. (2013, Char. 37).**
20. **Posterior margin of puboischiadic bar:** [0] straight or anteriorly directed, [1] posteriorly directed. **Villalobos-Segura *et al*. (2022, Char. 119).**
21. **Posterior process of the coracoid:** [0] absent, [1] present. Vullo *et al*. (2024, Char. 148)*. Based on Silva & Datovo (2020).
22. **Metapterygial whip:** [0] absent, [1] present. Coates *et al*. (2017), **Frey *et al*. (2020, Char. 200).**
23. **Sup Reduced number of cartilages between pelvic basipterygium and clasper:** [0] absent, **[1]** present. Villalobos-Segura *et al*. (2022, Char. 125).
24. **Sub Reduced number of cartilages between pelvic basipterygium and clasper (T3):** [0] not spinous, [1] spinous, [2] modified into external mesorhipidion. Vullo *et al*. (2024, Char. 151).
25. **Extended dorso and Ventral terminal cartilages** [0] absent, [1] present. **(Deactivated)**.

**Axial skeleton**

1. **Sup Chordacentra:** [0] absent, [1] present. Stahl (1999), Coates and Sequeira (2001), Coates *et al*. (2017), Frey *et al*. (2020, Char. 182).
2. **Sub Chordacentra polyspondylous and consist of narrow closely packed rings:** [0] absent, [1] present. Patterson (1965), Coates *et al*. (2017), Frey *et al*. (2020, Char. 183).
3. **Sup Calcified Vertebral Centra:** [0] absent, [1]. Modified from Vullo *et al*. (2024, Char. 156-162).
4. **Sub Calcified Vertebral Centra (Primary calcification):** [0] restricted terminally, [1] well-developed across the whole body. Modified from Vullo *et al*. (2024, Char. 156-162), Landemaine *et al*. (2018, Char. 183), Shirai (1996, Char. 151 & 152)***,** **Shirai (1992, Char. 75)**.
5. **Sub Calcified Vertebra Centra (Primary calcification):** [0] absent, [1] endochordal radii radiating from the notochordal sheath, [2] with developed solid medialia and diagonal calcified lamellae, [3] compact mass. Modified from Vullo *et al*. (2024, Char. 156-162), Jambura *et al*. (2023, Char. 130), Landemaine *et al*. (2018, Char. 184), **Shirai (1996, Char. 76)**.

**Remarks:** Characters 155-160 of Vullo *et al*. (2024) were modified. The initial codification aimed to follow a regionalization of the body and the modifications that the vertebral centra could present across these regions (e.g., fusions, synarcuals or expansion of the basiventral elements). However, in order to manage the number of state of characters that TNT can handle (no more than 32) we kept the primary and secondary calcifications as separate characters (Char. 164-168) but it created a conflict as character 164 and 166 coded for the absence or presence of these types of calcifications which provided redundant information with that of character 158 which codified for the presence or absence of calcified vertebral centra in Vullo *et al.* (2024). Consequently, in the present work we tried to avoid this by using reductive coding for the types of calcifications present. We used a multistate coding for character 153 as we are uncertain of the mechanisms restricting or controlling the expression of the secondary calcification, whether the genetic mechanisms for this type of calcification are present but silenced or they are missing.

1. **Cervical vertebra:** [0] absent, [1] present, [2] fused with free vertebra reaching caudally to suprascapula, [3] fused, reaching rostrally, passing the scapula, [4] fused, free vertebra reaching rostrally to suprascapula. Modified from Vullo *et al*. (2024, Char. 156-162).

**Remarks:** With the codification changes for characters 151-153 in the present study, characters 154, 156 and 157 were considered as a separate character, this unfortunately produced an increase of weight in the absence of vertebra centra (Char. 151) as in these the absence is considered again. The use of reductive coding is perhaps the better option for these characters (151-158). However, currently this is not possible as the implementation of this coding strategy, generates more than 32 states of characters which could not be handled currently in TNT. Consequently, we opted to code them as separate characters with multiple states. Furthermore, considering them as separate character allow us to consider a multistate scoring and incorporate it using a character state tree (Chars. 154 and 156), from which we try to account for the increase of weight into the absent state.


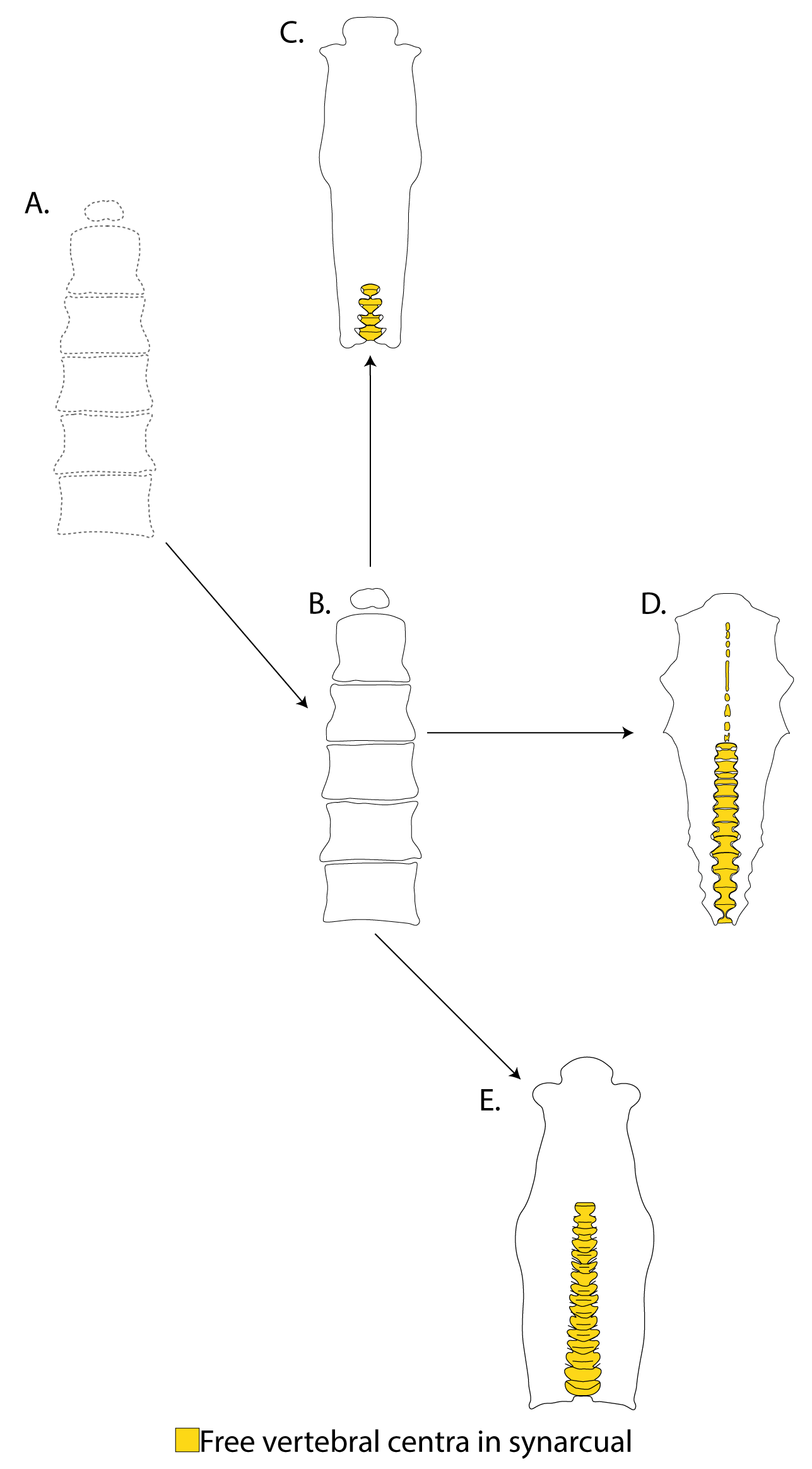


**Figure** **S7.** Enforced path using the “cstree” command on TNT for state evolution on character 154. Arrows indicate the allowed transformation path initiating for the state [0], each stop on a state increases one step. Line drawings illustrating the states scored the character. A. not mineralized vertebra centra. B. mineralized vertebra centra. C. Okamejei kenojei (Bürger, in Muller & Henle, 1841) (redrawn from Claeson, 2011, text-fig. 4C, ZMB 15512). D. Platyrhinoidis triseriata (Jordan & Gilbert, 1880). E. Glaucostegus typus (Bennett, 1830) (NHMUK 1967.2.11.3). State [0]: A; State [1]: B; State [2]: C. State [3]: D. State [4]: E

1. **Second synarcual:** [0] absent, [1] present. Villalobos-Segura *et al*. (2022, Char. 88), Aschliman *et al*. (2012, Char. 54), McEachran *et al*. (1996, Char. 43), **Nishida (1990, Char. 66**).
2. **Occipital hemicentrum:** [0] absent, **[1]** present, [2] demineralize, [3] fused (part of the synarcual). Vullo *et al*. (2024, Char. 155)*, Villalobos-Segura *et al*. (2022, Char. 50), Landemaine *et al*. (2018, Char. 16), Klug (2010, Char. 16), de Carvalho (1996, Char. 17), de Carvalho & Maisey (1996, Char. 29), Shirai (1996, Char. 21), **Shirai (1992, Char. 53).**
3. **Caudal vertebrae:** [0] absent, [1] diplospondylus, [2] fused. Villalobos-Segura *et al*. (2022, Char. 89), **Aschliman *et al*. (2012, Char. 80**).
4. **Lateral stays:** [0] absent, [1] dorsally directed and free of medial crest, [2] laterally directed and free from medial crest, [3] dorsally directed and fused to medial crest. Modified from Vullo *et al*. (2024, Char. 164), Villalobos-Segura *et al*. (2022, Char. 52)*, **Villalobos-Segura *et al*. (2019, Char. 53).**


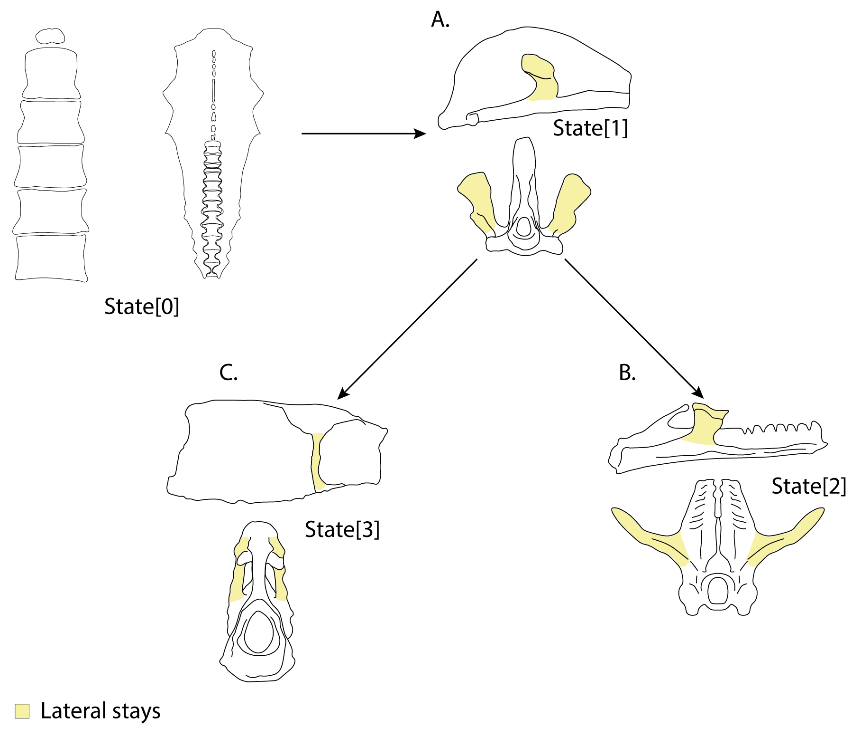


**Figure** **S8.** Enforced path using the “cstree” command on TNT for state evolution on character 158. Arrows indicate the allowed transformation path initiating for the state [0], each stop on a state increases one step. A. Rhina ancylostoma Bloch & Schneider, 1801 (LACM 38117-38, https://sharksrays.org/, accessed on 20 Oct. 2023), B. Torpedo fuscomaculata Peters, 1855 (https://sharksrays.org/, accessed on 20 Oct. 2023), C. Mobula munkiana Notarbartolo di Sciara, 1987 (SIO 85-34) (https://sharksrays.org/, accessed on 20 Oct. 2023).

1. **Hemal arch:** [0] not arched at anterior precaudal tail vertebrae, [1] almost complete in the entire region of the precaudal tail. Landemaine *et al*. (2018, Char. 96), Klug (2010, Char. 97(42)), de Carvalho (2004, Char. 47), Shirai (1996, Char. 78), **Shirai (1992, Char. 159**).
2. **Sup Supraneurals:** [0] absent [1] present. Landemaine *et al*. (2018, Char. 62), & 91(39) Klug (2010, Char. 62(38)), **de Carvalho & Maisey (1996, Char. 69), Shirai (1992, Char. 156**).
3. **Sup Supraneurals (plate-like supraneurals):** [0] enlarged at least in front of the second dorsal fin, [1] also enlarged in the abdominal region. Landemaine *et al*. (2018, Char. 62 & 90), Klug (2010, Char. 62(38) & 91(39)), de Carvalho & Maisey (1996, Char. 70)***, Shirai (1992, Char. 157**).
4. **Vertebral ribs:** [0] absent, [1] present [2] reduced. Vullo *et al*. (2024 Char. 172)*, Villalobos-Segura *et al*. (2022, Char. 90), Aschliman *et al*. (2012, Char. 44), Klug (2010, Char. 105), McEachran & Aschliman (2004, Char. 49), McEachran *et al*. (1996, Char. 44), Shirai (1996, Char. 74), **Shirai (1992, Char. 158)**. Modified from Nishida (1990, Char. 64).
5. **Pleural ribs:** [0] absent, [1] present. Vullo *et al*. (2024, Char. 173)*. Based on Maisey (1982) and Coates and Gees (2007, Char. 16).
6. **Arcualia dorsalis:** [0] absent, [1] present. Villalobos-Segura *et al*. (2022, Char. 54)*, **Brito *et al*. (2013, Char. 30)**.
7. **Number of dorsal fins:** [0] one, [1] two, [2] reduced. Landemaine *et al*. (2018, Char. 145), Klug (2010, Char. 149(89)), de Carvalho (2004, Char. 50), **de Carvalho & Maisey (1996, Char. 83)**, Shirai (1996, Char. 80).

**Remarks:** State [2] is added to include some taxa like the sting rays which do not present well-developed dorsal fins.

1. **Dorsal fin:** [0] aplesodic, [1] plesodic. Jambura *et al*. (2023, Char. 152). Modified from Shimada (2005, Char. 35) and Shirai (1996, Char. 83).
2. **Dorsal fin endoskeleton:** [0] composed of a triangular or rectangular basal cartilage and radials separated from vertebral column, [1] composed of a triangular or rectangular basal cartilage and radials close to vertebral column, [2] radials only (no basal) parallel to each other, [3] at least two large plates, [4] small radials variously arranged no basal, [5] elongate basal with radials. Vullo et al. (2024, Char. 177). Modified from Jambura *et al*. (2023, Char. 155), Shirai (1996, Char. 162-166), taken from Compagno (1990).


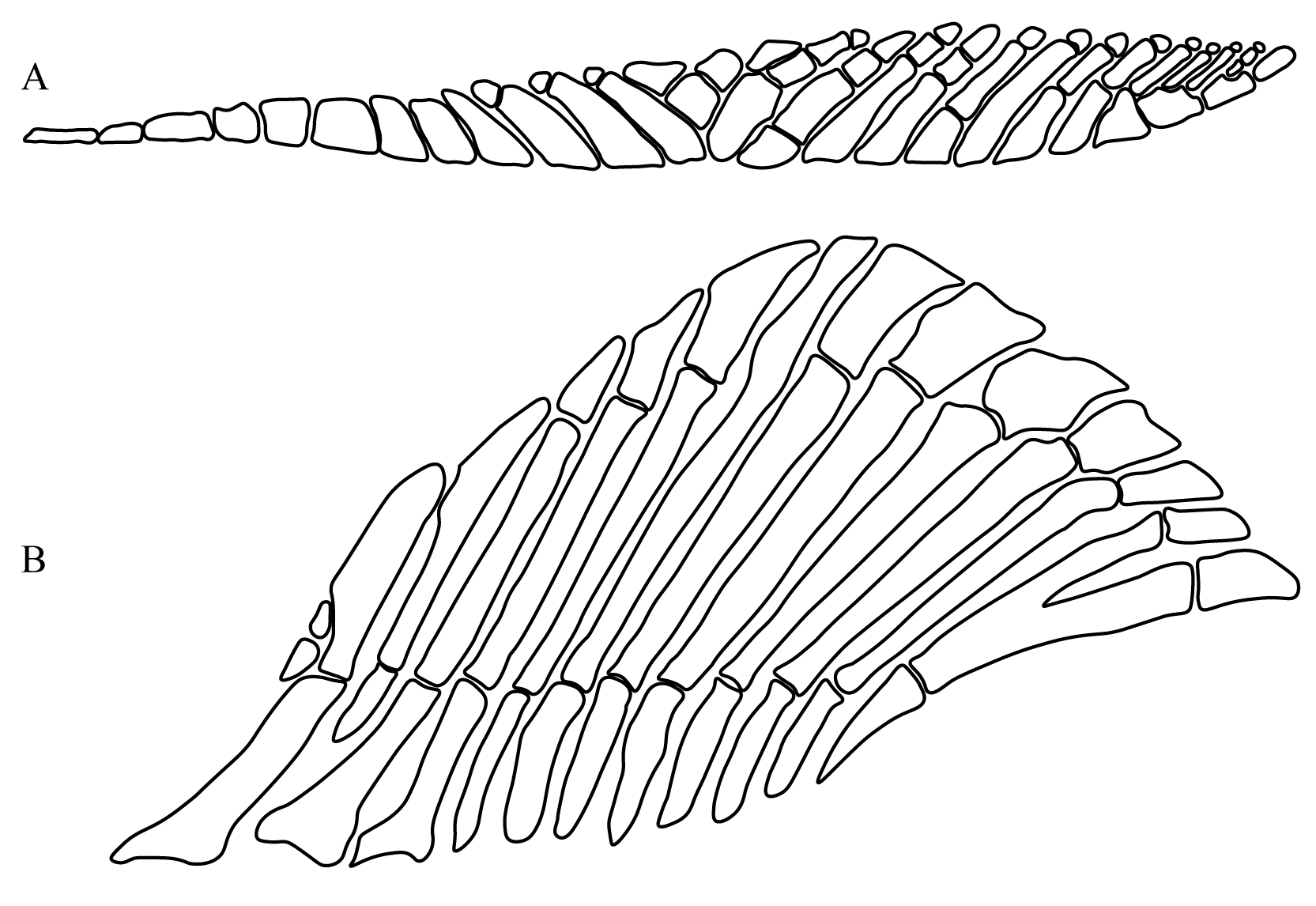


Figure S9. Line drawing of some of the character states scored for character 167. A. Chlamydoselachus anguineus Garman, 1884 (redrawn and modified from Shirai 1992, Plate. 54A, MSM-88-40). B. Heterodontus zebra (Gray, 1831) (redrawn and modified from Shirai 1992, Plate. 54A, HUMZ 37666). State[2]: B, State [4]: A.

1. **Caudal fin:** [0] with a developed lower lobe to make a "lunate" form, [1] heterocercal, [2] reduced to the plesodic or tail folds, [3] without any tail folds or finlet. **Shirai (1996, Char. 86)**. Modified from Nishida (1990, Char. 56).
2. **Caudal lower lobe:** [0] without radials, [1] with radials. **Frey *et al*. (2020, Char. 213**).
3. **Procoracoid mineralisation:** [0] absent, [1] present. **Frey *et al*. (2020, Char. 196**). Based on Davis (2002), Brazeau (2009),
4. **Posterior dorsal fin with delta-shaped cartilage:** [0] absent, [1] present. **Frey *et al*. (2020, Char. 209**). Based on Coates & Sequeira (2001).

**External features and lateral line features**

1. **Cephalic lobes:** [0] absent, [1] present. Villalobos-Segura *et al*. (2022, Char. 61), Aschliman *et al*. (2012, Char. 10), McEachran *et al*. (1996, Char. 9), **Nishida (1990, Char. 96)**.
2. **Upper eyelid:** [0] present, [1] absent. Villalobos-Segura *et al*. (2022, Char. 1), Aschliman *et al*. (2012, Char. 1), **McEachran *et al*. (1996, Char. 1)**.
3. **Nictitating eyelid:** [0] absent, [1] present. **Shimada (2005, Char. 25)**.
4. **Nostrils:** [0] separated, [1] close together. **Villalobos-Segura *et al*. (2022, Char. 36).**
5. **Anterior nasal lobe:** [0] fails to reach mouth, [1] reaches the mouth. Villalobos-Segura *et al*. (2022, Char. 31), **Aschliman *et al*. (2012, Char. 11)**.
6. **Anterior nasal lobe:** [0] fails to cover most of the medial half of the naris, [1] well-developed. Villalobos-Segura *et al*. (2022, Char. 32)*. Modified from **Aschliman *et al*. (2012, Char. 11)**.
7. **Nasal curtain fringes: [**0] absent, [1] present. Villalobos-Segura *et al*. (2022, Char. 33).
8. **Infraorbital loop of suborbital and infraorbital canals:** [0] absent, [1] present. Villalobos-Segura *et al*. (2022, Char. 81), Aschliman *et al*. (2012, Char. 21), **McEachran *et al*. (1996, Char. 15**).
9. **Subpleural loop of the hyomandibular canal:** [0] broad rounded, [1] loop forms a lateral hook, [2] lateral aspects of subpleural loop are nearly parallel. Villalobos-Segura *et al*. (2022, Char. 82), Aschliman *et al*. (2012, Char. 22), **McEachran *et al*. (1996, Char. 16).**
10. **Sup_Abdominal canal on coracoid bar:** [0] absent, [1] present. **Villalobos-Segura *et al*. (2022, Char. 83),** Aschliman *et al*. (2012, Char. 24), McEachran *et al*. (1996, Char. 18).
11. **Sub_Abdominal canal on coracoid bar (if present):** [0] groove-cephalic lateral line forms abdominal canal on coracoid bar, [1] pores. **Villalobos-Segura *et al*. (2022, Char. 84)**.
12. **Scapular loops of scapular canals:** [0] absent, [1] present. Villalobos-Segura *et al*. (2022, Char. 86), Aschliman *et al*. (2012, Char. 25), **McEachran *et al*. (1996, Char. 19)**.
13. **Cephalic lateral line canals on ventral surface:** [0] present, [1] absent. Villalobos-Segura *et al*. (2022, Char. 87), Aschliman *et al*. (2012, Char. 20), McEachran *et al*. (1996, Char. 14).
14. **Sup Cephalic spines:** [0] absent, [1] present. Vullo *et al*. (2024, Char. 195-197), Coates and Gess (2007, Char. 24), (**Maisey, 1989, Char. 14**).
15. **Sub Cephalic spines (Trilobed cephalic spine base):** [0] absent, [1] present. Vullo *et al*. (2024, Char. 195-197)*, Coates and Gess (2007, Char. 44).
16. **Sub Cephalic spines (Number of pairs):** [0] one, [1] two. Vullo *et al*. (2024, Char. 195-197)* , (**Maisey, 1989, Char. 35**).
17. **Sub Cephalic spines (Multicuspid):** [0] no multiple cuspid, [1] present and with multiple cuspids. Vullo *et al*. (2024, Char. 195-197)*, Coates and Gess (2007, Char. 15), (**Maisey, 1989, Char. 31**).
18. **Placoid scales:** [0] scarce or absent, [1] present. Villalobos-Segura *et al*. (2022, Char. 133)., Aschliman *et al*. (2012, Char. 15), **McEachran & Dunn (1998, Char. 11**).
19. **Malar and alar thorns:** [0] absent, [1] present. Villalobos-Segura *et al*. (2022, Char. 134), Aschliman *et al*. (2012, Char. 17), **McEachran & Dunn (1998, Char. 22).**
20. **Lateral rostral dermal denticles:** [0] absent, [1] present. **Villalobos-Segura *et al*. (2022, Char. 136)**.
21. **Sup Dorsal fin spines:** [0] absent, [1] present. Vullo *et al*. (2024, Chars. 202, 204-207)*, Villalobos-Segura *et al*. (2022, Char. 130), Goto (2001, Char. 12a), **de Carvalho (2004, Char. 49**).
22. **Cranial dorsal spine:** [0] absent, [1] present. Frey *et al*. (2020, Char. 216).
23. **Sub Dorsal fin spines (Vascularization):** [0] vascularized, [1] unvascularized. Vullo *et al*. (2024, Chars. 202, 204-207)*. Based on Maisey (1977).
24. **Sub Dorsal fin spines (posterior denticles):** [0] absent, [1] present. Vullo *et al*. (2024, Chars. 202, 204-207)*, **Frey *et al*. (2020, Char. 229)**.
25. **Sub Dorsal fin spines (Fin spines ornamentation):** [0] absent, [1] present. Vullo *et al*. (2024, Chars. 202, 204-207)*, **Frey *et al*. (2020, Chars. 227-228**).
26. **Dorsal fin spine apex curved posteriorly:** [0] absent, [1] present. Vullo *et al*. (2024, Chars. 202, 204-207)*, **Frey *et al*. (2020, Chars. 218)**.
27. **Serrated tail sting:** [0] absent, [1] present. Villalobos-Segura *et al*. (2022, Char. 132), Aschliman *et al*. (2012, Char. 14), **McEachran *et al*. (1996, Char. 13)**.
28. **Enameloid structure:** [0] Single layer enameloid (no PBE), [1] **Mesh paralell bundle enamelod and radial enameloid**. Modified from Vullo *et al*. (2024, Char. 209-2010)*, Jambura *et al*. (2023, Char. 181)*, Landemaine *et al*. (2018, Char. 159), Klug (2010, Char. 163(103)). Based on Reif (1973, 1977a, 1977b, 1980), Compagno (1977), Cuny *et al*. (2001), Cuny & Risnes (2005), Enault *et al*. (2013), Manzanares *et al.* (2018), Maisey *et al*. (2019). Based on Enault et al., (2015).
29. **Lingual torus:** [0] absent, [1] present. **Frey *et al*. (2020, Char. 71)**.
30. **Differentiated lateral uvulae on teeth:** [0] absent, [1] present. Villalobos-Segura *et al*. (2022, Char. 56), **Claeson *et al*. (2013, Char. 22).**
31. **Crown dentine:** [0] orthodentine, [1] osteodentine, [2] pseudoostedentine (ortho + osteodentine). **Jambura *et al*. (2023, Char. 193).**
32. **Sup Basal furrows on root:** [0] absent, [1] present. Modified from Klug (2010, Char. 166(106)). Based on Cappetta (1987, 1992, 2012) and Thies (1991, 1993).
33. **Sub Basal furrows on root (Compression):** [0] absent, [1] present. **Vullo *et al*. (2024, Char. 215)**. Based on Cappetta (1987, 1992, 2012) and Thies (1991, 1993).
34. **Teeth with distal heel** [0] absent, [1] present. Pollerspöck & Straube (2022, Char. 20), **Flammensbeck *et al*. (2018, Char. 10)**.
35. **Labial root depression:** [0] absent, [1] present. Klug, 2010 (Char. 170(110)). Based on Cappetta (1987, 1992, 2012) and Thies (1991, 1993).
36. **Root dentine:** [0] osteodentine, [1] orthodentine. Jambura *et al*. (2023, Char. 195), Villalobos-Segura *et al*. (2022, Char. 57), **Aschliman *et al*. (2012, Char. 19)**.
37. **Teeth with three slim main cusps almost equal to each other, strongly recurved:** [0] absent, [1] present. **Frey *et al*. (2020, Char. 73).**
38. **Toothplates:** [0] absent, [1] present. **Frey *et al*. (2020, Char. 74)**. Based on Patterson (1965) and Stahl (1999).
39. **Tubular dentine:** [0] absent, [1] present. **Frey *et al*. (2020, Char. 5)**. Based Stahl (1999) and Patterson (1965).
40. **Scapular process tip:** [0] wide, [1] narrow. (New).

**Remarks:** This character refers to the tip of the scapular process and whether this element finishing on an acute or a wide tip.

# **Additional miscellaneous modifications**

**Characters 60 and 99** of Vullo *et al*. (2024) repeated information and were difficult to determinate in most fossil species. Consequently, both characters were removed.

# **Institutional abbreviations**

**AC.UERJ**: Fish Collection of the Departamento de Zoologia at the Universidade do Estado do Rio de Janeiro, Brazil. A**MNH**: American Museum of Natural History, USA. **BHN:** Musèe d'Histoire Naturelle de Broulogne-sur-Mcr, France. **BRC:** Birkbeck Reference Collection, UK. **CAS**: California Academy of Sciencies, San Francisco, California, USA **CNPE-IBUNAM**: Colección Nacional de Peces del Instituto de Biología, Universidad Nacional Autónoma de México, México**. CSIRO**: Commonwealth Scientific and Industrial Research Organization, Australia. **FMNH**: Field Museum of Natural History, USA. **GPIT**: ﻿Geologisch-Paläontologisches Institut, Universität Tübingen, Germany**. GMBL**: College of Charleston, Grice Marine Biological Laboratory, Charleston, South Carolina, USA. **HUMZ**: Hokkaido University Laboratory of Marine Zoology, Japan. **JME-SOS:** Jura Museum Eichtätt, Eichtätt, Germany**. LACM**: Los Angeles County Museum of Natural History, USA. **MCZ**: Museum for Comparative Zoology, Cambridge, USA. ﻿**MJML**: Museum of Jurassic Marine Life Kimmeridge, UK. **MHNL**: Musée des Confluences, Lyon, France. **MNHN** Muséum National d’Histoire Naturelle, Paris. **MSM**: Marine Science Museum, Tokai University, Japan. **NMMB(HO)**: National Museum of Marine Biology and Aquarium, Taiwan**. NHMUK** Natural History Museum United Kingdom, UK. **NMS:** National Museum of Scotland, Edinburgh, Scotland**. ROM**: Royal Ontario Museum, Ontario, Canada. **USNM**: National Museum of Natural History, USA. Field Museum of Natural History, USA. **SIO**: Scripps Institution of Oceanography, USA. **SMNS**: Staatliches Museum für Naturkunde Stuttgart, Germany. **SNSB-BSPGM**: ﻿Bayerische Staatssammlung für Paläontologie und Geologie, Munich, Germany. **UF**: University of Florida, Florida State Museum, USA. **USNM**: National Museum of Natural History, USA. **ZMB**: Museum für Naturkunde zu Berlin, Germany.

**﻿Revised fossil material**

***Archaeogracilidens macer* (**SMNS 96844-7, SMNS 80142-44).

***Acronemus ﻿tuberculatus*** (﻿Maisey, 2011; Rieppel, 1982).

***﻿Asteracanthus ornatissimus*** (Stumpft *et al*., 2021a).

***﻿Asterotrygon maloneyi*** (de Carvalho et al., 2004).

***Belemnobatis sismondae*** (﻿MHNL15.263, 15.264, 15.753).

﻿***Cladoselache*** (Maisey 1989b, 2007; ﻿Tomita, 2015; Coates *et al*., 2017)***.***

***Cladodoides*** (﻿Coates **et al**., 2017; Maisey 2005).

***Chondrenchelys problematica*** (﻿Finarelli & Coates, 2014)**.**

***Cobelodus aculeatus*** (Schaeffer, 1981; Maisey, 2007)**.**

**﻿*Cretoxyrhina mantelli*** (Shimada, 1997; Newbrey et al., 2015).

***﻿Cyclobatis major*** (MNHN 1939- 13-334A, MNHN HAK555. NHMUK PV P 4010, 4011, 49514 63175). ***Cyclobatis radians*** (NHMUK P 61243). ***Cyclobatis tuberculatos*** (NHMUK PV P 10436). ***Cyclobatis oligodactylus*** (NHMUK PV P 601). ***Cyclobatis* sp.** (AMNH 10946).

**﻿*Debeerius ellefseni*** ﻿(ROM PV 43173*﻿*: Grogan & Lund, 2000)*.*

***﻿Diplodoselache woodi*** (﻿Dick, 1981).

***Doliodus ﻿latispinosus*** (﻿Milller et al., 2003; Maisey *et al*., 2014, 2017, 2019; Coates et al., 2017; 2018; Frey et al., 2019, 2020).

***﻿Dracopristis hoffmanorum*** (Hodnett *et al*., 2021).

***Egertonodus basanus*** (﻿NHMUK PV P 40718, 2082, 60110, 6356: ﻿Maisey 1982, 1983; Maisey & Lane, 2010; Lane, 2010).

***Gutturensis neilsoni*** (Cast ROM PV 86720: ﻿Sequeira & Coates 2000).

**﻿*Hamiltonichthys mapesi*** (﻿Maisey, 1989a).

***﻿Helodus simplex*** (﻿Patterson, 1965; Stahl, 1999).

﻿***Heliobatis radians*** (de Carvalho et al., 2014).

***Hybodus hauffianus*** (SMNS 54048, ﻿15150; Stumpf et al., 2021b). **﻿*Hybodus reticulatus*** (Maisey, 1987).

***Iniopera*** (﻿Zangerl & Case 1973; Stahl, 1980; Pradel *et al*., 2011; Pradel 2010; Dearden et al., 2023).

***Kawichthys moodiei*﻿** (Pradel et al., 2011).

***Kimmerobatis etchesi*** (MJML K874, K1894; Underwood & Claeson, 2019)**.** ***﻿***

***﻿﻿Notidanoides muensteri*** (GPIT Pi 1210/3 1210/3, Maisey, 1986).

***Onychoselache traquairi*** (﻿Dick & Maisey 1980; Coates & Gess 2007).

***Orthacanthus*** (﻿Hampe 2003; Heidtke, 1999; ﻿Soler-Gijon, 1997).

***﻿Paracestracion falcifer*** (﻿SNSB-BSPGM AS VI 505)

***Phoebodus saidselachus*** (Frey et al., 2019).

***Protospinax annectans*** (JME-SOS 3386; NHMUK PV P 8775a,b,c, 37014; SNSB-BSPG 1963-I-19: Jambura *et al*., 2023)**.**

***Pseudorhina alifera*** (﻿NHMUK PV P8535, JME-SOS 438**).**

**﻿﻿*Pucapampella rodrigae*** (﻿Janvier & Suárez-Riglos, 1986; Maisey et al., 2019; Frey et al., 2020).

***Ptychodus*** (Vullo *et al.*, 2024).

***Squalicorax falcatus*** (Shimada & Cicimurri, 2005), ***Squalicorax pristodontus*** (Shimada & Cicimurri, 2006)

***Spathobatis bugesicus* (**NHMUK PV P 6010, 2099 (2), 12067, **SNSB-BSPGM** 1952-I-82, AS-I-505**). *Spathobatis moorbergensis*** (BHN 2P1. 2.4**).**

***﻿Tamiobatis vetustus*﻿ (﻿**Schaeffer 1981**). *Tamiobatis* sp. (﻿**Schaeffer 1981**).**

**﻿*Tingitanius tenuimandibulus* (**NHMUK PV P 66857, Claeson et al., 2013),

***Thrinacoselache﻿ gracia*** (﻿Grogan & Lund 2008).

***﻿*** ***﻿Triodus aeduorum*** (﻿Luccisano et al., 2021).

**﻿*Tristychius arcuatus*** (﻿Dick, 1978; Coates & Gess, 2007; Coates & Tietjen, 2017; Coates et al., 2019).

# Revised extant material

**﻿** **﻿*Aptychotrema vincentiana*** (CSIRO 101 https://sharksrays.org/).

***Bathyraja leucomelanos*** (MNHN 2005-2740). ﻿***Bathyraja schroederi*** (Stehmann & Pompert, 2014). ***Bathyraja*** (McEachran, 1984; ﻿Dolganov, 2020).

***Brachaelurus waddi*** (USNM 39998 https://sharksrays.org/)

***Carcharhinus plumbeus*** (GMBL 79-60 https://sharksrays.org/). ***Carcharhinus falciformis*** (de Olivera Lana *et al*., 2021).

**Carcharodon carcharias** (MCZ 171013 https://sharksrays.org/).

***Chimaera cubana*** (USNM 400700 https://sharksrays.org/). **﻿*Chimaera monstrosa*** (Dean *et al*., 2011).

﻿***Chlamydoselachus anguineus*** (MSM-88-40, UF 44302 <https://sharksrays.org/>, da Silva & de Carvalho, 2015). ***Chlamydoselachus africana*** (Ebert & Compagno, 2009).

***﻿Etmopterus lucifer*** (CAS-SU(ICH) 6863, Shirai, 1992).﻿ ***Etmopterus pusillu*** (da Silva & de Carvalho, 2015). ***Etmopterus splendidus*** (AMNH 258170 <https://sharksrays.org/>)

***Glaucostegus typus*** (NHMUK 1967.2.11.3, 2012.2.8.54, 1926.5.26.5).

***Ginglymostoma cirratum*** (USNM 127110 <https://sharksrays.org/>). ***Gymnura altavela*** (GMBL 81-86 <https://sharksrays.org/>). ***Gymnura japonica*** (CAS ICH 42165, Nishida, 1990). ***Gymnura marmorata*** (CAS SU1158). ***Gymnura micrura*** (FMNH 89990).

**﻿*Dalatias licha*** (AMNH 4582 <https://sharksrays.org>, Shirai, 1992).

***Deania calceus*** (AMNH MB85-015114 <https://sharksrays.org>, Shirai, 1992).

***Hemipristis elongata* (**LACM 37712-1 <https://sharksrays.org>).

***Hemiscyllium ocellatum*** (AMNH 44128 <https://sharksrays.org>).

***Heptranchias perlo*** (GMBL 96-12 <https://sharksrays.org>).

***Heterodontus francisci*** (AMNH 96795 <https://sharksrays.org>**).**

***Hexanchus nakamurai*** (UF 165855 <https://sharksrays.org>**).**

***Hexatrygon bickelli*** (CAS ICH 233779). ***Hexatrygon*** (﻿Heemstra & Smith, 1980).

***﻿Hydrolagus affinis*** (BRC—Hydrolagus),

***Hypnos monopterygius*** (USNM 84374 <https://sharksrays.org/>).

***Isurus oxyrinchus*** (GMBL 8446 <https://sharksrays.org/>).

***Mobula munkiana*** (SIO 85-34 https:// sharksrays.org).

***Mustelus manazo*** (AMNH 258162 <https://sharksrays.org/>).

**﻿*Narcine brasiliensis*** (CNPE-IBUNAM 9280, AMNH 77069 <https://sharksrays.org/>).

***Narcine entemedor* (**CNPE-IBUNAM 5807).

***Narcine tasmaniensis*** (NHMUK 1961).

**﻿Narke japonica** (Nishida, 1990).

**Notorynchus cepedianus** (Maisey, 2004).

**﻿*Odontaspis noronhai*** (Stone & Shimada, 2019).

***Orectolobus japonicus*** (NHMUK 1862.11.1.18). ﻿***Orectolobus maculatus*** (Goto, 2001).

***Oxynotus centrina* (USNM 206065** [**https://sharksrays.org/**](https://sharksrays.org/)**,** Shirai, 1992).

***Parascyllium collare*** (Goto, 2001).

***Platyrhinoidis triseriata*** (MNHN 4329, 3211, USNM 26275 https://sharksrays.org/).

***Platyrhina sinensis*** (MNHN 1307, AMNH 44055)**, *Platyrhina* sp** (BRC—*Platyrhina*, CT scan)

**﻿*Pliotrema warreni*** (NHMUK 1905.6.8.9).

**Potamotrygon limai** (﻿Fontenelle *et al*., 2014). ***Potamotrygon motoro*** (AMNH 97428 <https://sharksrays.org/>).

**Pristiophorus japonicus** (da Silva & de Carvalho, 2015). ***Pristiophorus lanae*** (CAS 34930). ***Pristiophorus nudipinnis*** (Mollen et al., 2016).  **﻿**

***Pseudocarcharias kamoharai*** (LACM 45857 https://sharksrays.org/).

**﻿*Pristis pectinata*** (FMNH 1939)**. *Pristis* sp*.* (**BRC—*Pristis***)*.* *Pristis zijsron*** (da Silva & de Carvalho, 2015; da Silva & Datovo, 2020).

***Raja eglanteria*** (GMBL 02-155, also GMBL 5557, ﻿https:// sharksrays.org).

***﻿﻿Raja clavata*** (BRC—Raja, CT scan)**.**

***Rhina ancylostoma*** (LACM 38117-38, <https://sharksrays.org/>).

***Rhinobatos lentiginosus*** (GMBL 74-37, <https://sharksrays.org/>).

***Rhinoptera bonasus* (**BRC—*Rhinoptera***,** GMBL73 <https://sharksrays.org>). ***Rhinoptera javanica*** (Nishida, 1990). ***Rhinoptera jayakari*** (Pradeep et al., 2018).

***Rhizoprionodon terraenovae*** (GMBL uncatalogued <https://sharksrays.org>).

***Rhynchobatus springeri*** (AMNH 258310 <https://sharksrays.org>).

***Scyliorhinus meadi*** (GMBL 8312 <https://sharksrays.org>).

***Sphyrna lewini*** (USNM 203101 <https://sharksrays.org>). ***Sphyrna media*** (USNM 205377 <https://sharksrays.org>). ***Sphyrna tiburo* (**AMNH uncatalogued <https://sharksrays.org>**). *Sphyrna tudes*** (USNM 159197 <https://sharksrays.org>). ***Sphyrna zygaena* (**USNM 325631 <https://sharksrays.org>).

***Squalus acanthias*** (GMBL 7313, <https://sharksrays.org>). ***Squalus brevirostris* (AMNH 258171,** <https://sharksrays.org>**).**

***Squatina africana*** (Mollen *et al*., 2016). ***Squatina nebulosa*** (AMNH 258172 <https://sharksrays.org>).

***Scyliorhinus haeckelii*** (Soares *et al*., 2016).

***Torpedo fuscomaculata*** (USNM 320677 <https://sharksrays.org>).

***Zameus squamulosus*** (USNM 400734, CT scan).

**﻿*Zapteryx exasperata*** (CNPE-IBUNAM 17822, 17823, 17824, 17826, 17825, 20528). ***Zapteryx xyster* (**CNPE-IBUNAM 16661, 19790).

***Trygonorrhina fasciata*** (McEachran *et al*., 1996; Aschliman *et al*., 2012).

# **Revision of Klug (2010) analysis**


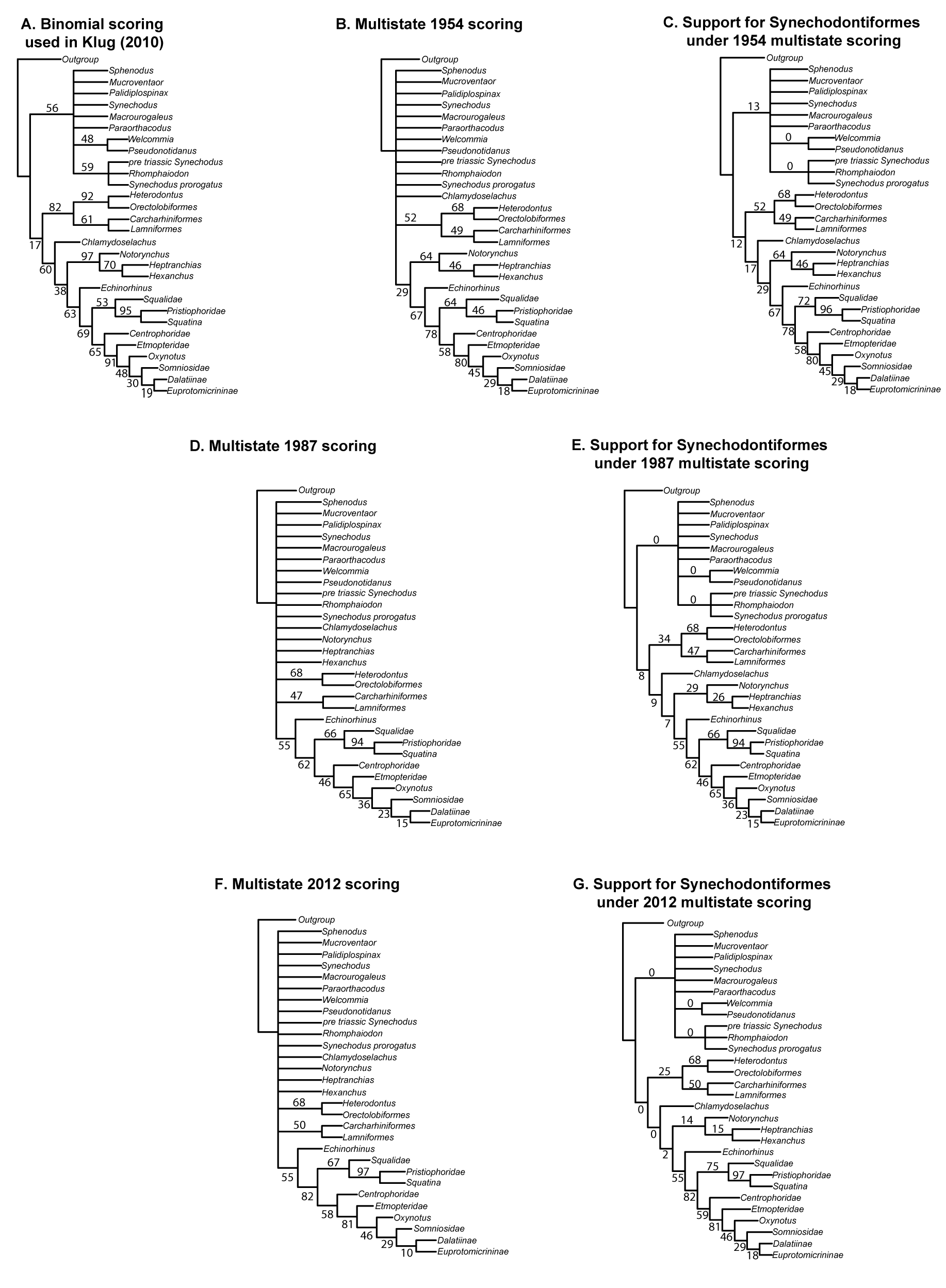


Figure. S10. Results on the analyses carried with Klug (2010) data matrix and character state different scoring for the root vascularization types and other independent binary characters.

Klug (2010) employed a strictly binary scoring scheme for root vascularization patterns, in which each pattern was included as independent binary characters (i.e., absent/present or yes/no). This scoring is not recommended to use as separating a multistate character into a series of absent/present characters produces an illogical assignment of synapomorphies for nodes, as one of the character states can be optimized as a synapomorphy supporting a node without even presenting it between the terminals in the node. This happens because one of the tokens (characters states) is transformed into a non-specific variable, (i.e., a waste-basket or catch-all for anything that simply implies differences to x features) without any consideration of the actual state displayed by the terminals. In Klug (2010) present several instances of this type of coding (see Supplementary Materials Klug matrix Chars 145-147, 155-161 and 165–169).This type of coding affected the results of Klug (2010) analysis, resulting in the formation of a Synechodontiformes clade. The synechodontiform clade is recovered if these characters, are coded as unordered multistate characters. The use of unordered multistate coding method avoids illogical assumptions and reconstructions of ancestral states, but also avoids the subjective interpretations reading directions of character state changes, this coding proposed to avoid the issues risen by the nonadditive binary coding structure. When an unordered multistate coding is implemented in Klug (2010) the analysis fails to recover the Synechodontiformes clade (Figure S10), suggesting that this clade is the result of the application of a problematic coding strategy.

# References

Agassiz LJR. 1833-1844 *Recherches sur les Poissons Fossiles*; Petitpierre: Neuchâtel, Switzerland; Volumes 1–5.

Allis, E. P. (1914). The pituitary fossa and trigemino-facialis chamber in selachians. *Anatomischer Anzeiger.* (46), 225–253.

Allis, E. P. (1923). The cranial anatomy of *Chlamydoselachus anguineus. Acta Zoologica*, (4), 123–221.

Aschliman, N. C., Claeson, K. M., & McEachran, J. D. (2012). Phylogeny of Batoidea. In J. C. Carrier, J.A. Musick, & M. R. Heithaus (Eds.), *Biology of Sharks and Their Relatives,* (pp. 57–97). CRC Press.

Ayres, W. O. (1854). Description of new fishes from California. *Proceedings. California Academy of Sciences,* 1, 3–22.

de Beer, G. R. (1937). *The development of the vertebrate skull*. Oxford: Clarendon Press.

Bennett, E. T. (1830). Class Pisces. In Lady Stamford Raffles (Ed.)*, Memoir of the Life and Public Services of Sir Thomas Stamford Raffles,* (pp. 686–694)*.* John Murray*.*

Bloch, M. E., & Schneider, J. G. (1801). *M.E. Blochii Systema Ichthyologiae iconibus ex illustratum. Post obitum auctoris opus inchoatum absolvit, correxit, interpolavit*. J.G. Schneider, Saxo.

Bonnaterre, J. P. (1788). *Ichthyologie. Tableau encyclopédique et méthodique des trois règnes de la nature*. Chez PANCKOUCKE, Libraire, Hotel de Thou, rue des. Poitevins.

Brito, P. M., Leal, M. E. C., & Gallo, V. (2013). A new lower Cretaceous guitarfish (Chondrichthyes, Batoidea) from the Santana formation, Northeastern Brazil. Boletim do Museu Nacional, Geologia, 75, 1-13.

Brito, P. M., & Seret, B. (1996). The new genus *Iansan* (Chondrichthyes, Rhinobatoidea) from the Early Cretaceous of Brazil and its phylogenetic relationships. In G. Arritia, & V. Gunther (Eds.), *Mesozoic fishes - Systematics and Paleoecology,* (pp. 47–63). Verlag Dr. Friedrich Pfeil.

Brazeau, M. D. (2009). The braincase and jaws of a Devonian ‘acanthodian’and modern gnathostome origins. Nature, 457(7227), 305–308.

Bronson, A. W., Pradel, A., Denton, J. S., & Maisey, J. G. (2024). A new operculate symmoriiform chondrichthyan from the Late Mississippian Fayetteville Shale (Arkansas, United States). *Geodiversitas*, *46*(4), 101–117.

Burrow, C. J., den Blaauwen, J., Newman, M., & Davidson, R. (2016). The diplacanthid fishes (Acanthodii, Diplacanthiformes, Diplacanthidae) from the Middle Devonian of Scotland. *Palaeontologia Electronica,* (19), 1-83.

Cappetta, H. (1987). Chondrichthyes II: Mesozoic and Cenozoic Elasmobranchii. Verlag Dr. Friedrich Pfeil.

Cappetta, H. (1992). New observations on the palaeospinacid dentition (Neoselachii, Palaeospinacidae). *Neues Jahrbuch für Geologie und Paläontologie. Monatshefte*, (9), 565–570.

Cappetta H. (2012). Chondrichthyes. Mesozoic and Cenozoic Elasmobranchii: Teeth. Verlag Dr. Friedrich Pfeil.

de Carvalho, M. R. (1996). Higher-Level Elasmobranch Phylogeny, Basal Squaleans and Paraphyly. In, M. L. J. Stiassny, L. R. Parenti, & D. G Johnson (Eds.), *Interrelationships of fishes*, (pp. 35–60). Academic Press.

de Carvalho MR. 2004 Late Cretaceous Thornback Ray from Southern Italy, with a Phylogenetic Reappraisal of the Platyrhinidae (Chondrichthyes: Batoidea). In G. Arratia, & A. Tintori (Eds.), *Mesozoic Fishes 3 - Systematics, Paleoenvironments and Biodiversity*, (pp. 75–101). Verlag Dr. Friedrich Pfeil.

de Carvalho, M. R., & Maisey, J. G. (1996). Phylogenetic Relationships of the Late Jurassic Shark *Protospinax Woodward* 1919 (Chondrichthyes: Elasmobranchii). In G. Arritia & V. Gunther (Eds.), *Mesozoic fishes - Systematics and Paleoecology,* (pp. 9–47). Verlag Dr. Friedrich Pfeil.

de Carvalho, M. R., Maisey, J. G., & Grande, L. (2004). Freshwater stingrays of the Green River Formation of Wyoming (Early Eocene), with the description of a new genus and species and an analysis of its phylogenetic relationships (Chondrichthyes: Myliobatiformes). *Bulletin of the American Museum of Natural History*, 2004(284), 1–136.

Castex, M. N, & Castello, H. P. (1970). *Potamotrygon yepezi*, n. sp. (Condrichthyes, (sic) Potamotrygonidae), a new species of freshwater sting-ray from Venezuelan rivers. *Acta Scientifica, Universidad del Salvador*, (8), 15–39.

Claeson, K. M. (2011). The synarcual cartilage of batoids with emphasis on the synarcual of Rajidae. *Journal of morphology*, *272*(12), 1444–1463.

Claeson, K. M., Underwood, C. J., & Ward, D. J. (2013). †*Tingitanius tenuimandibulus*, a New Platyrhinid Batoid from the Turonian (Cretaceous) of Morocco and the Cretaceous Radiation of the Platyrhinidae. *Journal of vertebrate paleontology*, (33), 1019–1036.

Coates, M. I., & Gess, R. W. (2007). A new reconstruction of *Onychoselache traquairi*, comments on early chondrichthyan pectoral girdles and hybodontiform phylogeny. *Palaeontology*, 50(6), 1421–1446.

Coates, M. I., Gess, R. W., Finarelli, J. A., Criswell, K. E., & Tietjen, K. (2017). A symmoriiform chondrichthyan braincase and the origin of chimaeroid fishes. *Nature*, *541*(7636), 208–211.

Coates, M. I., Finarelli, J. A., Sansom, I. J., Andreev, P. S., Criswell, K. E, Tietjen, K., Rivers, M. L., & La Riviere, P. J. (2018). An early chondrichthyan and the evolutionary assembly of a shark body plan. *Proceedings of the Royal Society B: Biological Sciences,* (285), 20172418.

Coates, M. I., & Sequeira, S. E. K. (1998). The braincase of a primitive shark. *Earth and Environmental Science Transactions of the Royal Society of Edinburgh*, 89(2), 63–85.

Coates, M. I., & Sequeira, S. E. K. (2001). A new stethacanthid chondrichthyan from the Lower Carboniferous of Bearsden, Scotland. Journal of Vertebrate Paleontology, 21(3), 438–459.

Coates, M. I., & Tietjen, K. (2017). The neurocranium of the Lower Carboniferous shark *Tristychius arcuatus* (Agassiz, 1837). *Earth and Environmental Science Transactions of the Royal Society of Edinburgh*, 108(1), 19–35.

Compagno, L. J. (1977). Phyletic relationships of living sharks and rays. *American zoologist*, 17(2), 303–322.

Compagno, L. J. V. (1990). Relationships of the megamouth shark, *Megachasma pelagios* (Lamniformes: Megachasmidae), with comments on its feeding habits. *NOAA technical report NMFS*, 90, 357–379.

Compagno, L. J., & Last, P. R. (2010). A new species of wedgefish, Rhynchobatus springeri (Rhynchobatoidei, Rhynchobatidae), from the Western Pacific. Descriptions of new sharks and rays from Borneo. *CSIRO Marine and Atmospheric Research Paper*, 32, 77-88.

Cope, E. D. (1894). New and little known Paleozoic and Mesozoic fishes. *Journal of the Academy of Natural Sciences of Philadelphia*, 2, 427–448.

Cuny, G., & Risnes, S. (2005). The enameloid microstructure of the teeth of synechodontiform sharks (Chondrichthyes: Neoselachii). *PalArch’s Journal of Vertebrate Palaeontology*, 3(2), 1–19.

Cuny, C., Rieppel, O., & Sanders, P.M. (2001). The shark fauna from the Middle Triassic (Anisian) of north-western Nevada. *Zoological Journal of the Linnean Society*, *133*(3), 285–301.

Davis, S. P. (2002). *Comparative anatomy and relationship of the acanthodian fishes*. PhD thesis, University College London.

Davis, S. P., Finarelli, J. A., & Coates, M. I. (2012). Acanthodes and shark-like conditions in the last common ancestor of modern gnathostomes. Nature, 486(7402), 247–250.

Dean, M. N., Summers, A. P., & Ferry, L. A. (2012). Very low pressures drive ventilatory flow in chimaeroid fishes. *Journal of Morphology*, 273(5), 461-479.

Dearden, R. P., Herrel, A., & Pradel, A. (2023). Evidence for high-performance suction feeding in the Pennsylvanian stem-group holocephalan *Iniopera*. *Proceedings of the National Academy of Sciences*, 120(4), e2207854119.

Dick, J. R. (1978). On the carboniferous shark *Tristychius arcuatus* Agassiz from Scotland. *Earth and Environmental Science Transactions of The Royal Society of Edinburgh*, 70(4), 63-108.

Dick, J. R. (1981). *Diplodoselache woodi* gen. et sp. nov., an early Carboniferous shark from the Midland Valley of Scotland. *Earth and Environmental Science Transactions of The Royal Society of Edinburgh*, 72(2), 99-113.

Dick, J. R. & Maisey, J. G. (1980). The Scottish Lower Carboniferous shark *Onychoselache traquairi*. *Palaeontology*, (23), 363–374.

Dolganov, V. N. (2020). A Morphological description and the biology of poorly known deep-sea skates of the genus *Bathyraja* Ishiyama, 1958 (Rajidae) from the northwestern Pacific Ocean. *Russian Journal of Marine Biology*, 46, 73-80.

Dupret, V., Sanchez, S., Goujet, D., Tafforeau, P., & Ahlberg, P. E. (2014). A primitive placoderm sheds light on the origin of the jawed vertebrate face. *Nature*, 507(7493), 500–503.

Duméril, C. (1806). Zoologie Analytique, ou Méthode Naturelle de Classification des Animaux, Rendue Plus Facile à l’aide de Tableaux Synoptiques. Allais, Librairie, Quai des Augustins.

Ebert, D. A., & Compagno, L. J. (2009). *Chlamydoselachus africana*, a new species of frilled shark from southern Africa (Chondrichthyes, Hexanchiformes, Chlamydoselachidae). *Zootaxa*, 2173(1), 1-18.

Enault, S., Cappetta, H., & Adnet, S. (2013). Simplification of the enameloid microstructure of large stingrays (Chondrichthyes: Myliobatiformes): a functional approach. *Zoological Journal of the Linnean Society*, 169 (1), 144–155.

Enault, S., Guinot, G., Koot, M. B., & Cuny, G. (2015). Chondrichthyan tooth enameloid: past, present, and future. *Zoological Journal of the Linnean Society*, 174 (3), 549–570.

Finarelli, J. A., & Coates, M. I. (2014). Chondrenchelys problematica (Traquair, 1888) redescribed: a Lower Carboniferous, eel-like holocephalan from Scotland. *Earth and Environmental Science Transactions of the Royal Society of Edinburgh*, 105(1), 35-59.

Flammensbeck, C. K., Pollerspöck, J., Schedel, F. D., Matzke, N. J., & Straube, N. (2018). Of teeth and trees: A fossil tip‐dating approach to infer divergence times of extinct and extant squaliform sharks. Zoologica Scripta, 47(5), 539-557.

Fontenelle, J. P., Silva, J. P. C. B., & Carvalho, M. D. (2014). *Potamotrygon limai*, sp. nov., a new species of freshwater stingray from the upper Madeira River system, Amazon basin (Chondrichthyes: Potamotrygonidae). *Zootaxa*, 3765(3), 249-268.

Forskål, P. (1775). *Descriptiones animalium, avium, amphibiorum, piscium, insectorum, vermium: quae in itinere orientali observavit*. ex officina Mölleri.

Frey, L., Coates, M., Ginter, M., Hairapetian, V., Rücklin, M., Jerjen, I., & Klug, C. (2019). The early elasmobranch *Phoebodus*: phylogenetic relationships, ecomorphology and a new time-scale for shark evolution. *Proceedings of the Royal Society B*, 286(1912), 20191336.

Frey, L, Coates, M. I., Tietjen, K., Rücklin, M., & Klug, C. (2020). A Symmoriiform from the Late Devonian of Morocco Demonstrates a Derived Jaw Function in Ancient Chondrichthyans. *Communications biology*, (3), 1–10.

Gardiner, B. G. (1984). The relationships of the palaeoniscid fishes, a review based on new specimens of *Mimia* and *Moythomasia* from the Upper Devonian of Western Australia. Bulletin of the British Museum (Natural History), *Geology Series*, 37(4), 173–428.

Garman, S. (1884). An extraordinary shark. *Bulletin of the Essex Institute*, (16), 47–55.

Giles, S., Friedman, M., & Brazeau, M. D. (2015). Osteichthyan-like cranial conditions in an Early Devonian stem gnathostome. Nature, 520 (7545), 82–85.

Goloboff, P. A., De Laet, J., Ríos-Tamayo, D., & Szumik, C. A. (2021). A reconsideration of inapplicable characters, and an approximation with step‐matrix recoding. *Cladistics*, 37(5), 596–629.

Goodrich ES. 1930. Studies on the Structure and Development of Vertebrates. Macmillan and Company, Limited.

Goto T. (2001) Comparative Anatomy, Phylogeny and Cladistic Classification of the Order Orectolobiformes (Chondrichthyes, Elasmobranchii). *Memoirs of the Graduate School of Fisheries Sciences Hokkaido University*. (48), 1–100.

Gray, J. E. (1831). Description of three new species of fish, including two undescribed genera, discovered by John Reeves, Esq. China. *Zoological Miscellany*, 1(6), 4-5.

Grogan, E. D., & Lund, R. (2000). *Debeerius ellefseni* (fam. nov., gen. nov., spec. nov.), an autodiastylic chondrichthyan from the Mississippian Bear Gulch Limestone of Montana (USA), the relationships of the Chondrichthyes, and comments on gnathostome evolution. *Journal of Morphology*, 243(3), 219-245.

Grogan, E. D., & Lund, R. (2008). A basal elasmobranch, *Thrinacoselache gracia* n. gen and sp., (Thrinacodontidae, new family) from the Bear Gulch Limestone, Serpukhovian of Montana, USA. *Journal of Vertebrate Paleontology*, 28(4), 970-988.

Haacke, W. (1885). Diagnosen zweier bemerkenswerther südaustralischer Fische. *Zoologischer Anzeiger,* (8), 508–509.

Hampe, O. (2002). Revision of the Xenacanthida (Chondrichthyes: Elasmobranchii) from the Carboniferous of the British Isles. *Earth and Environmental Science Transactions of the Royal Society of Edinburgh*, 93(3), 191-237.

Heemstra, P. C., & MM, S. (1980). Hexatrygonidae, a new family of stingrays (Myliobatiformes: Batoidea) from South Africa, with comments on the classification of batoid fishes.

Heidtke, U. H. J. (1999). *Orthacanthus* (*Lebachacanthus*) *senckenbergianus* Fritsch 1889 (Xenacanthida: Chondrichthyes): revision, organisation und phylogenie. *Freiberger Forschungsheft C*, 481, 63-106.

Hodnett, J. P. M., Grogan, E. D., Lund, R., Lucas, S. G., Suazo, T., Elliott, D. K., & Pruitt, J. (2021). Ctenacanthiform sharks from the Late Pennsylvanian (Missourian) Tinajas member of the Atrasado formation, central New Mexico. *New Mexico Museum of Natural History and Science Bulletin*, 84, 391-424.

Holmgren, N. (1941). Studies on the head in fishes. Part 2. Comparative anatomy of the adult selachian skull with remarks on the dorsal fins in sharks. *Acta Zoológica*, 22: 1–100.

Jambura, P. L., Villalobos-Segura, E., Türtscher, J., Begat, A., Staggl, M. A., Stumpf, S., Kindlimann, R., Klug, S., Lacombat, F., Pohl, B., Maisey, G. J., Naylor, G. J. P., & Kriwet, J. (2023). Systematics and phylogenetic interrelationships of the enigmatic late Jurassic shark *Protospinax annectans* Woodward, 1918 with comments on the shark–ray sister group relationship. *Diversity*, 15(3), 311.

Janvier, P., & Suárez Riglos, M. (1986). The Silurian and Devonian vertebrates of Bolivia. *Bulletin de l’Institut français d’Études andines*, 15(3), 73-114.

Jordan, D. S., & Gilbert, C. H. (1880). Description of a new ray (*Platyrhina triseriata*), from the coast of California. *Proceedings of the United States National Museum*. (3), 36–38.

Jordan, D. S. Gilbert, C. H. (1883). Description of a new species of *Rhinobatus* *(Rhinobatus glaucostigma*) from Mazatlan, Mexico. Proceedings of the United States National Museum. 6, 210–211.

Klug, S. (2010). Monophyly, phylogeny and systematic position of the †Synechodontiformes (Chondrichthyes, Neoselachii). *Zoologica scripta*, 39(1), 37–49.

Lane J. A. (2010) Morphology of the braincase in the Cretaceous hybodont shark *Tribodus limae* (Chondrichthyes: Elasmobranchii), based on CT scanning. *American Museum Novitates*, (2010**)**, 1–70.

Landemaine O, Thies D, Waschkewitz J. (2018) The Late Jurassic Shark *Palaeocarcharias* (Elasmobranchii, Selachimorpha) – Functional Morphology of Teeth, Dermal Cephalic Lobes and Phylogenetic Position. *Palaeontographica, Abteilung A*. (312), 103–165.

Linné, C. (1758). *Systema Naturae per regna tria naturae, regnum animale, secundum classes, ordines, genera, species, cum characteribus differentiis synonymis, locis*. *Laurentii Salvii.*

Lu, J., Giles, S., Friedman, M., den Blaauwen, J. L., & Zhu, M. (2016). The oldest actinopterygian highlights the cryptic early history of the hyperdiverse ray-finned fishes. *Current Biology*, 26(12), 1602–1608.

Luccisano, V., Pradel, A., Amiot, R., Gand, G., Steyer, J. S., & Cuny, G. (2021). A new *Triodus* shark species (Xenacanthidae, Xenacanthiformes) from the lowermost Permian of France and its paleobiogeographic implications. *Journal of Vertebrate Paleontology*, 41(2), e1926470.

Maisey, J. G. (1977). The fossil selachian fishes *Palaeospinax* Egerton, 1872 and *Nemacanthus* Agassiz, 1837. *Zoological Journal of the Linnean Society*, 60(3), 259–273.

Maisey, J. G. (1982). The Anatomy and Interrelationships of Mesozoic Hybodont Sharks. *American Museum Novitates*. 2724, 1–48.

Maisey, J. G. (1983). Cranial anatomy of *Hybodus basanus* Egerton from the Lower Cretaceous of England. *American Museum Novitates*, 2758, 1–64.

Maisey, J. G. (1984). Higher elasmobranch phylogeny and biostratigraphy. Zoological *Journal of the Linnean Society*, 82(1-2), 33-54.

Maisey, J. G. (1985) Cranial Morphology of the Fossil Elasmobranch *Synechodus dubrisiensis*. *American Museum Novitates*. 2804, 1–28.

Maisey, J. G. (1986). The Upper Jurassic hexanchoid elasmobranch *Notidanoides* n. g. *Neues Jahrbuch für Geologie und Paläontologie Abhandlungen*, 172(1), 83–106.

Maisey, J. G. (1987). Cranial anatomy of the Lower Jurassic shark *Hybodus reticulatus* (Chondrichthyes, Elasmobranchii): with comments on hybodontid systematics. *American Museum Novitates*. 2878. 1–39.

Maisey, J. G. (1989a). *Hamiltonichthys mapesi*, g. & sp. nov.(Chondrichthyes, Elasmobranchii), from the Upper Pennsylvanian of Kansas. *American Museum Novitates*, 2931, 1–42.

Maisey, J. G. (1989b). Visceral skeleton and musculature of a Late Devonian shark. Journal of Vertebrate Paleontology, 9(2), 174-190.

Maisey, J. G. (2001). A primitive chondrichthyan braincase from the Middle Devonian of Bolivia. In P.E. Ahlberg (Ed.), *Major events in early vertebrate evolution*, (pp. 263–288). Taylor & Francis.

Maisey, J. G. (2005). Braincase of the Upper Devonian shark Cladodoides wildungensis (Chondrichthyes, Elasmobranchii), with observations on the braincase in early chondrichthyans. *Bulletin of the American Museum of Natural History,* 2005(288), 1–103.

Maisey, J. G. (2007). The braincase in Paleozoic symmoriiform and cladoselachian sharks. *Bulletin of the American Museum of Natural History*, 2007(307), 1-122.

Maisey, J. G. (2008). The postorbital palatoquadrate articulation in elasmobranchs. *Journal of Morphology*, 269 (8), 1022-1040.

Maisey, J. G. (2011). The braincase of the Middle Triassic shark *Acronemus tuberculatus* (Bassani, 1886). *Palaeontology*, 54(2), 417-428.

Maisey, J. G., Janvier, P., Pradel, A., Denton, J. S., Bronson, A., Miller, R., & Burrow, C. J. (2019). *Doliodus* and pucapampellids: contrasting perspectives on stem chondrichthyan morphology. In Z. Johanson, C. Underwood, & M. Richter (Eds.), *Evolution and development of fishes*, (pp. 87-109). Cambridge University Press.

Maisey, J. G., & Lane, J. A. (2010). Labyrinth morphology and the evolution of low-frequency phonoreception in elasmobranchs. *Comptes Rendus Palevol*, 9(6-7), 289-309.

Maisey, J. G., Miller, R., Pradel, A., Denton, J. S., Bronson, A., & Janvier, P. (2017). Pectoral morphology in *Doliodus*: bridging the ‘acanthodian’-chondrichthyan divide. *American Museum Novitates*, 2017(3875), 1-15.

Maisey, J. G., Turner, S., Naylor, G. J., & Miller, R. F. (2014). Dental patterning in the earliest sharks: implications for tooth evolution. *Journal of morphology*, 275(5), 586-596.

Marramà, G., Klug, S., De Vos, J., & Kriwet, J. (2018). Anatomy, relationships and palaeobiogeographic implications of the first Neogene holomorphic stingray (Myliobatiformes: Dasyatidae) from the early Miocene of Sulawesi, Indonesia, SE Asia. Zoological Journal of the Linnean Society, 184(4), 1142-1168.

Manzanares, E., Botella, H., & Delsate, D. (2018). On the enameloid microstructure of Archaeobatidae (Neoselachii, Chondrichthyes). *Journal of Iberian Geology*, 44, 67–74.

McEachran, J. D. (1984). Anatomical investigations of the New Zealand skates *Bathyraja asperula* and *B*. *spinifera*, with an evaluation of their classification within the Rajoidei (Chondrichthyes). *Copeia*, 45-58.

McEachran, J. D., & Aschliman N. (2004). Phylogeny of Batoidea. In J.C. Carrier, J. A. Musick, & M. R. Heithaus (Eds.), *Biology of Sharks and their Relatives* (pp. 79–109). CRC Press.

McEachran, J. D., & Dunn, K. A. (1998). Phylogenetic analysis of skates, a morphologically conservative clade of elasmobranchs (Chondrichthyes: Rajidae). *Copeia*, 271-290.

McEachran, J. D., Dunn, K. A., & Miyake, T. (1996) Interrelationships of the batoid fishes (Chondrichthyes: Batoidea). In M. L. Stiassny, L. R. Parenti, & G. D. Johnson (Eds.), *Interrelationships of fishes* (pp. 63–83). Atlantic Press.

Miller, R. F., Cloutier, R., & Turner, S. (2003). The oldest articulated chondrichthyan from the Early Devonian period. *Nature*, 425(6957), 501-504.

de Miranda Ribeiro, A. (1907). Fauna Braziliense. Peixes. II. Desmobranchios. Arquivos do Museu Nacional do Rio de Janeiro, (14), 131–217

Miyake, T., McEachran, J. D., Walton, P. J., & Hall, B. K. (1992). Development and morphology of rostral cartilages in batoid fishes (Chondrichthyes: Batoidea), with comments on homology within vertebrates. *Biological Journal of the Linnean Society*, 46(3), 259–298.

Mollen, F. H., van Bakel, B. W., & Jagt, J. W. (2016). A partial braincase and other skeletal remains of Oligocene angel sharks (Chondrichthyes, Squatiniformes) from northwest Belgium, with comments on squatinoid taxonomy. *Contributions to Zoology*, 85(2), 147-171.

Müller, J., & Henle, F. G. J. (1837). Gattungen der Haifische und Rochen nach einer von ihm mit Hrn. Henle unternommenen gemeinschaftlichen Arbeit über die Naturgeschichte der Knorpelfische. *Ber.Akad.Wiss.Berlin*. **1837**, 111–118.

Müller, J., & Henle, J. (1841). *Systematische beschreibung der Plagiostomen*. *Verlag Von Veit und Comp*.

Newbrey, M. G., Siversson, M., Cook, T. D., Fotheringham, A. M., & Sanchez, R. L. (2013). Vertebral morphology, dentition, age, growth, and ecology of the large lamniform shark *Cardabiodon rick*i. *Acta Palaeontologica Polonica*, 60(4), 877-897.

Nishida K. (1990). Phylogeny of the Suborder Myliobatidoidei. *Memoirs of the Graduate School of Fisheries Sciences Hokkaido University.* (37**)**, 1–108.

Notarbartolo-di-Sciara, G. (1987). A revisionary study of the genus *Mobula* Rafinesque, 1810 (Chondrichthyes: Mobulidae) with the description of a new species. *Zoological Journal of the Linnean Society*, *91*(1), 1–91.

von Olfers, I. F. M. (1831). Die Gattung Torpedo in ihren naturhistorischen und antiquarischen Beziehungen erläutert. Gedruckt in der Druckerei der Königlichen Akademie der Wissenschaften.

de Oliveira Lana, F., Soares, K. D., Hazin, F. H. V., & Gomes, U. L. (2021). Description of the chondrocranium of the silky shark *Carcharhinus falciformis* with comments on the cranial terminology and phylogenetic implications in carcharhinids (Chondrichthyes, Carcharhiniformes, Carcharhinidae). *Journal of morphology*. (282), 685–700.

Peters, W. (1855). Ubersicht der in Mossambique beobachteten Seefische. *Monatsschr Akad Wiss Berlin*, 1855, 428–466.

Patterson, C. (1965). The phylogeny of the chimaeroids. *Philosophical Transactions of the Royal Society B,* 249, 101-219.

Pollerspöck, J., & Straube, N. (2021). Phylogenetic placement and description of an extinct genus and species of kitefin shark based on tooth fossils (Squaliformes: Dalatiidae). *Journal of Systematic Palaeontology*,19(15), 1083-1096.

Pradel, A. (2010). Skull and brain anatomy of late Carboniferous Sibyrhynchidae (Chondrichthyes, Iniopterygia) from Kansas and Oklahoma (USA). Geodiversitas, 32(4), 595-661.

Pradel, A., Tafforeau, P., Maisey, J. G., & Janvier P. (2011). A new Paleozoic Symmoriiformes (Chondrichthyes) from the Late Carboniferous of Kansas (USA) and cladistic analysis of early chondrichthyans. *PLoS One*, (6), e24938.

Pradel, A., Didier, D., Casane, D., Tafforeau, P., & Maisey, J. G. (2013). Holocephalan embryo provides new information on the evolution of the glossopharyngeal nerve, metotic fissure and parachordal plate in gnathostomes. *PLoS One*, 8(6), e66988.

Pradeep, H. D., Swapnil, S. S., Nashad, M., Venu, S., Ranjan, K. R., Sumitha, G., ﻿Monalisha D. S., & Farejiya, M. K. (2018). First record and DNA barcoding of Oman cownose ray, *Rhinoptera jayakari* Boulenger, 1895 from Andaman Sea, India. *Zoosystema*, 40(1), 67-74.

Qiao, T., King, B., Long, J. A., Ahlberg, P. E., & Zhu, M. (2016). Early gnathostome phylogeny revisited: multiple method consensus. *PloS one*, *11*(9), e0163157.

Reif, W. E, (1973). Morphologie und Ultrastruktur des Hai‐“Schmelzes”. *Zoologica scripta*, 2 (5‐6), 231–250.

Reif, W. E. (1977a). Tooth enameloid as a taxonomic criterion: 1. A new eusalachian shark from the Rhaetic-Liassic boundary. *Neues Jahrbuch für Geologie und Paläontologie, Monatshefte*, 1977 (9): 565–576.

Reif, W. E. (1977b). Tooth enameloid as a taxonomic criterion. 2. Is "*Dalatias*" barnstonensis sykes, 1971 (Triassic, England) a squalomorphic shark?. *Neues Jahrbuch für Geologie und Paläontologie*. 1978(1). 42–58.

Reif, W. E. (1980). Tooth enameloid as a taxonomic criterion: 3. A new primitive shark family from the lower Keuper. *Neues Jahrbuch für Geologie und Paläontologie, Abhandlungen*, 160, 61–72.

Rippel O. (1982). A new genus of shark from the Middle Triassic of Monte San Giorgio Switzerland. *Palaeontology*. (25). 399–412.

Schaeffer B. (1981). The xenacanth shark neurocranium, with comments on elasmobranch monophyly. *Bulletin of the American Museum of Natural History*. 169, 1–72.

Sequeira, S. E. K., & Coates, M. I. (2000). Reassessment of ‘*Cladodus’* *neilsoni* Traquair: a primitive shark from the Lower Carboniferous of East Kilbridge, Scotland. *Palaeontology*, 43(1), 153-172.

Shimada, K. (1997). Paleoecological relationships of the Late Cretaceous lamniform shark, *Cretoxyrhina mantelli* (Agassiz). *Journal of Paleontology*, 71(5), 926-933.

Shimada, K. (2005). Phylogeny of lamniform sharks (Chondrichthyes: Elasmobranchii) and the contribution of dental characters to lamniform systematics. *Paleontological Research*, 9 (1), 55–72.

Shimada, K., & Cicimurri, D. J. (2005). Skeletal anatomy of the Late Cretaceous shark, *Squalicorax* (Neoselachii: Anacoracidae). *Palaeontologische Zeitschrift*, 79, 241-261.

Shimada, K., & Cicimurri, D. J. (2006). ﻿The oldest record of the Late Cretaceous Anacoracid Shark, *Squalicorax pristodontus* (Agassiz), from the Western Interior, with comments on *Squalicorax* Phylogeny. Late Cretaceous vertebrates from the Western Interior. ﻿New Mexico Museum of Natural History and Science Bulletin, (35), 177–184.

Shirai, S. (1992). *Squalean phylogeny: a new framework of" squaloid" sharks and related taxa*. Hokkaido University Press.

Shirai S. (1996). Phylogenetic Interrelationships of Neoselachians (Chondrichthyes: Euselachii). In In, M. L. J. Stiassny, L. R. Parenti, & D. G Johnson (Eds.), *Interrelationships of fishes*, (pp. 9–34). Academic Press.

da Silva, J. P. C. B., & Datovo, A. (2020). The coracoid bar and its phylogenetic importance for elasmobranchs (Chondrichthyes). Zoologischer Anzeiger, 287, 167-177.

da Silva, J. P. C., & de Carvalho, M. R. (2015). Morphology and phylogenetic significance of the pectoral articular region in elasmobranchs (Chondrichthyes). *Zoological Journal of the Linnean Society*, 175(3), 525–568.

da Silva, J. P. C., Vaz, D. F., & de Carvalho, M. R. (2018). Phylogenetic inferences on the systematics of squaliform sharks based on elasmobranch scapular morphology (Chondrichthyes: Elasmobranchii). Zoological Journal of the Linnean Society, 182(3), 614–630.

Soares, K. D., Gomes, U. L, & Carvalho, M. R. (2016) Taxonomic review of catsharks of the *Scyliorhinus haeckelii* group, with the description of a new species (Chondrichthyes: Carcharhiniformes: Scyliorhinidae). *Zootaxa*, (4066), 501-534.

Soler-Gijón, R. (1997). New discoveries of xenacanth sharks from the Late Carboniferous of Spain (Puertollano Basin) and Early Permian of Germany (Saar-Nahe Basin): implications for the phylogeny of xenacanthiform and anacanthous sharks. *Neues Jahrbuch für Geologie und Paläontologie-Abhandlungen*, 1-31.

Stahl, B. J. (1999), Handbook of Paleoichthyology: Chondrichthyes III: Mesozoic and Cenozoic Elasmobrachii: Teeth. Verlag Dr. Friedrich Pfeil.

Stehmann, M. F., & Pompert, J. H. (2014). First mature male record of *Bathyraja schroederi* (Elasmobranchii, Arhynchobatidae) from the South Atlantic, with descriptions of its clasper and skeletal characters. *Zootaxa*, 3838(4), 401-422.

Stone, N. R., & Shimada, K. (2019). Skeletal anatomy of the bigeye sand tiger shark, *Odontaspis noronhai* (Lamniformes: Odontaspididae), and its implications for lamniform phylogeny, taxonomy, and conservation biology. *Copeia*, 107(4), 632-652.

Stumpf, S., López‐Romero, F. A., Kindlimann, R., Lacombat, F., Pohl, B., & Kriwet, J. (2021a). A unique hybodontiform skeleton provides novel insights into Mesozoic chondrichthyan life. *Papers in Palaeontolog*y, 7(3), 1479-1505.

Stumpf, S., Etches, S., Underwood, C. J., & Kriwet, J. (2021b). *Durnonovariaodus maiseyi* gen. et sp. nov., a new hybodontiform shark-like chondrichthyan from the Upper Jurassic Kimmeridge Clay Formation of England. *PeerJ*, 9, e11362.

Stahl, B. J. (1980). Non-autostylic Pennsylvanian iniopterygian fishes. Palaeontology, (23). 315–324.

Temminck CJ, Schlegel H. 1850 Pisces. In P. F de Siebold (Ed.), *Fauna Japonica, sive descriptio animalium quae in itinere per Japoniam suscepto annis 1823–30 collegit, notis observationibus et adumbrationibus illustravit.* (pp. 1–323). Lugduni Batavorum, Apud Auctorem.

Thies, D. (1991). *Palaeospinax*, *Synechodus* and/or *Paraorthacodus*?. The problem of palaeospinacid genera (Pisces, Neoselachii, Palaeospinacidae). Neues Jahrbuch für Geologie und Paläontologie. Monatshefte, (9), 549–552.

Thies, D. (1993). *Palaeospinax*, *Synechodus* and/or *Paraorthacodus*. Is the problem of palaeospinacid genera (Pisces, Neoselachii) solved?. Neues Jahrbuch für Geologie und Paläontologie. Monatshefte, 1993(12), 724–732.

Thomas, D. B., Hiscox, J. D., Dixon, B. J. and Potgieter, J. (2016). 3D scanning and printing skeletal tissues for anatomy education. Journal of anatomy, 229 (3), 473-481.

Tomita, T. (2015). Pectoral fin of the Paleozoic shark, Cladoselache: new reconstruction based on a near-complete specimen. *Journal of Vertebrate Paleontology*, 35(5), e973029.

Underwood, C. J., & Claeson, K. M. (2019). The Late Jurassic ray *Kimmerobatis etchesi* gen. et sp. nov. and the Jurassic radiation of the Batoidea. *Proceedings of the Geologists' Association*, 130(3-4), 345-354.

Villalobos-Segura, E., Marramà, G., Carnevale, G., Claeson, K. M., Underwood, C. J., Naylor, G. J. P., Kriwet J. (2022). The Phylogeny of Rays and Skates (Chondrichthyes: Elasmobranchii) Based on Morphological Characters Revisited. *Diversity,* (14), 1–65.

Villalobos-Segura, E., Underwood, C. J., Ward, D. J., & Claeson, K. M. (2019) The First Three-Dimensional Fossils of Cretaceous Sclerorhynchid Sawfish: *Asflapristis cristadentis* gen. et sp. nov., and Implications for the Phylogenetic Relations of the Sclerorhynchoidei (Chondrichthyes). *Journal of Systematic Palaeontology,* (17), 1847–1870.

Vullo, R., Villalobos-Segura, E., Amadori, M., Kriwet, J., Frey, E., González-González, M. A., Padilla-Gutiérrez, J. M., Ifrim, C., Stinnesbeck, E. S., Stinnesbeck, W. (2024). Exceptionally preserved shark fossils from Mexico elucidate the long-standing enigma of the Cretaceous elasmobranch *Ptychodus*. *Proceedings of the Royal Society B: Biological Sciences*, (291), 20240262.

Zangerl, R., & Case, G. R. (1973). Iniopterygia: a new order of Chondrichthyan fishes from the Pennsylvanian of North America. Field Museum Press.

Zhu, M., Ahlberg, P., Pan, Z., Zhu, Y., Qiao, T., Zhao, W., Jia, L., & Lu, J. (2016). A Silurian maxillate placoderm illuminates jaw evolution. *Science* (354), 334–336.

Zhu, M., Yu, X., Ahlberg, P. E., Choo, B., Lu, J., Qiao, T., Zhao, L.J., Blom, H., & Zhu, Y. (2013). A Silurian placoderm with osteichthyan-like marginal jaw bones. *Nature*, *502* (7470), 188–193.

Zhu, M., Zhao, W., Jia, L., Lu, J., Qiao, T., & Qu, Q. (2009). The oldest articulated osteichthyan reveals mosaic gnathostome characters. Nature, 458 (7237), 469–474.
